# Supplementary material for: Neoadjuvant sintilimab plus chemotherapy in EGFR-mutant NSCLC: Phase 2 trial interim results (NEOTIDE/CTONG2104)
Source: Cell Rep Med. 2024 Jun 18;5(7):101615. doi: 10.1016/j.xcrm.2024.101615 (PMC11293361; doi:10.1016/j.xcrm.2024.101615)

Cell Reports Medicine, Volume *5*

Supplemental information

Neoadjuvant sintilimab plus chemotherapy in EGFR-mutant NSCLC: Phase 2 trial interim results (NEOTIDE/CTONG2104)

Chao Zhang, Yu-Xuan Sun, Ding-Cheng Yi, Ben-Yuan Jiang, Li-Xu Yan, Ze-Dao Liu, Li-Shan Peng, Wen-Jie Zhang, Hao Sun, Zhi-Yong Chen, Dan-Hua Wang, Di Peng, Song-An Chen, Si-Qi Li, Ze Zhang, Xiao-Yue Tan, Jie Yang, Zhang-Yi Zhao, Wan-Ting Zhang, Jian Su, Yang-Si Li, Ri-Qiang Liao, Song Dong, Chong-Rui Xu, Qing Zhou, Xue-Ning Yang, Yi-Long Wu, Ze-Min Zhang, and Wen-Zhao Zhong

Supplementary Figure

Supplementary Fig.1 Clinical and multi-omics exploratory study design of CTONG2104 (NEOTIDE), related to Figure 1 and STAR Methods. Tx, treatment; EGFR, epidermal growth factor receptor; ECOG, Eastern Cooperative Oncology Group; MPR, major pathological response; pCR, pathological complete response; ORR, objective response rate; EFS, event-free survival; OS, overall survival; CN, chemotherapy; WES, whole-exome sequencing; WBC, whole blood cells; MRD, minimal residual disease.

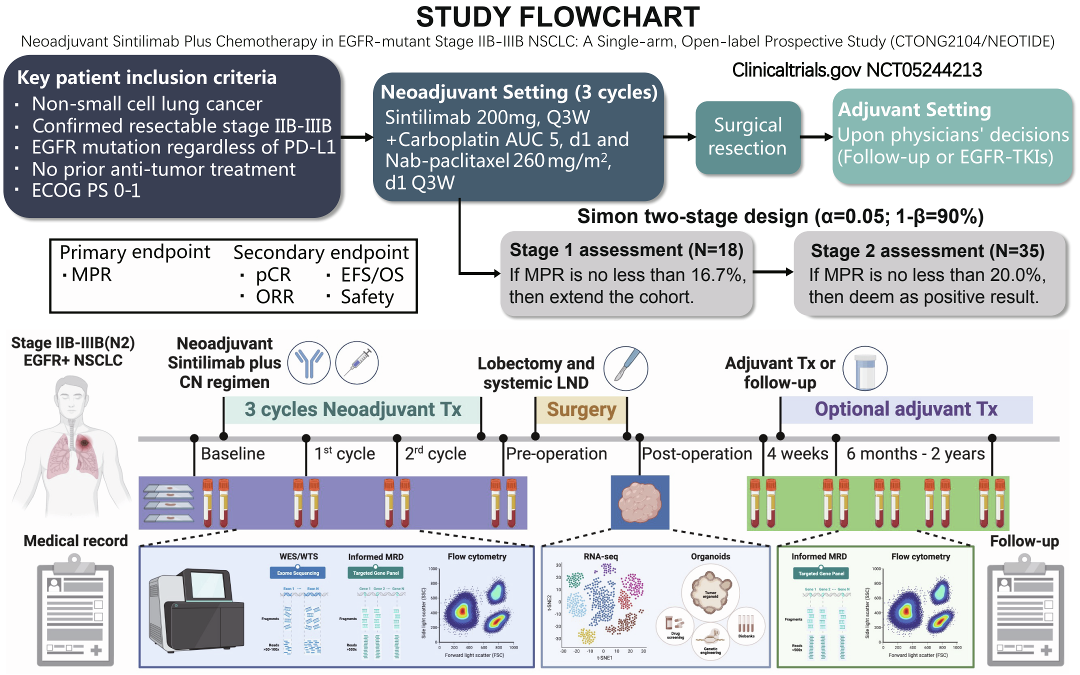

Supplementary Fig.2 Pathological response evaluation procedures, related to Figure 2 and STAR Methods. a Process of tumor sampling, preparation and assessment. **b** Schematic of counted components during pathological assessment including viable tumor, necrosis and stroma. **c** Specific pathological evaluation across different slides in a representative case. Red circles indicated viable tumor area. PLC, primary lung cancer; DLN, draining lymph nodes; RVT, residual viable tumor.

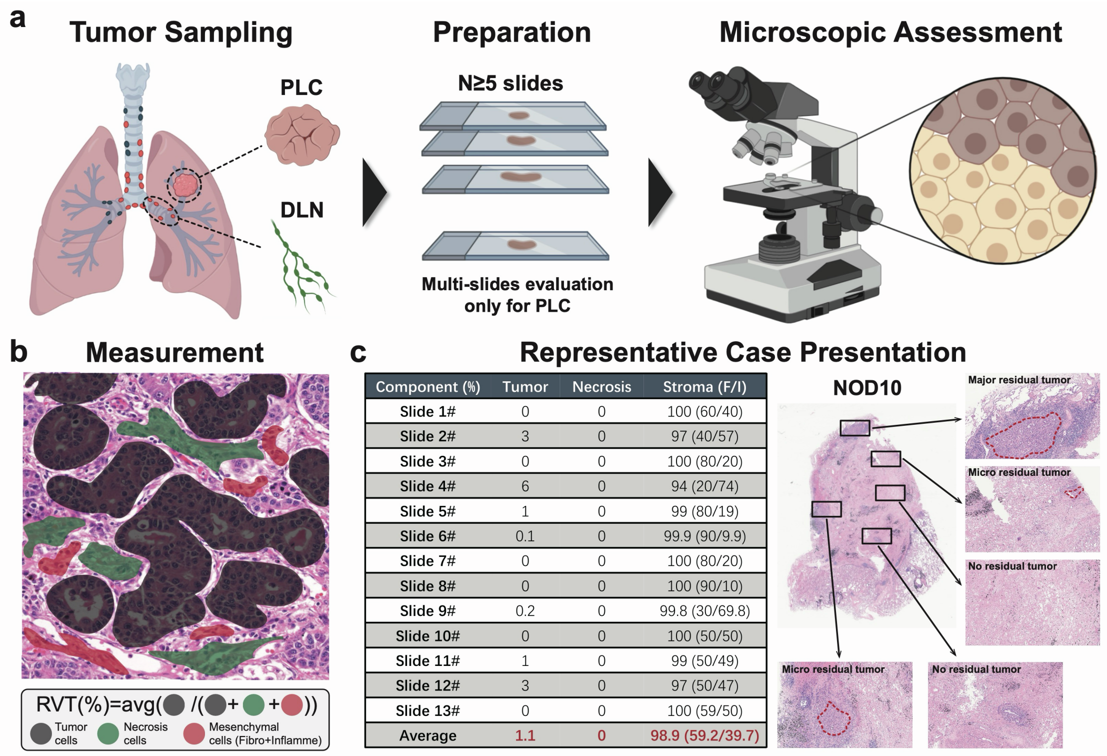

Component (%)	Tumor	Necrosis	Stroma (F/I)
Slide 1#	0	0	100 (60/40)
Slide 2#	3	0	97 (40/57)
Slide 3#	0	0	100 (80/20)
Slide 4#	6	0	94 (20/74)
Slide 5#	1	0	99 (80/19)
Slide 6#	0.1	0	99.9 (90/9.9)
Slide 7#	0	0	100 (80/20)
Slide 8#	0	0	100 (90/10)
Slide 9#	0.2	0	99.8 (30/69.8)
Slide 10#	0	0	100 (50/50)
Slide 11#	1	0	99 (50/49)
Slide 12#	3	0	97 (50/47)
Slide 13#	0	0	100 (59/50)
Average	1.1	0	98.9 (59.2/39.7)

Supplementary Fig.3 Correlation of MPR status with clinicopathological features and peripheral blood test, related to Figure 2 and STAR Methods. a Associations between clinicopathological characteristics (smoking, staging, EGFR subtypes and PD-L1 levels) and pathological response. Wilcoxon-rank sum test was used to measure the significance. **b** Comparison between relevant clinical factors and MPR status where no significant difference was found. **c** Flowcytometry analysis of PBMC regarding major cell lineage before and after neoadjuvant treatment. T test was used to measure the significance. $^{**}p<0.01$, $^{***}p<0.001$. **d** Dynamic changes of $CD3^{+}CD4^{+}$T cells and $CD19^{+}$B cells across different time points. Two-way ANOVA was used to calculate the significance of each time point between MPR and non-MPR, and Wilcoxon signed rank test was used to measure the significance between baseline and pre-surgery within each group. **e** Comparison of plasma IL6/IL8 before and after neoadjuvant immunochemotherapy. Wilcoxon signed rank test was used to measure the significance. **f** Difference of plasma IL8 before and after neoadjuvant treatment in MPR and non-MPR cohort, respectively. PRR, pathological regression rate; NLR, neutrophil/lymphocyte ratio; LMR, lymphocyte/monocyte ratio; MPR, major pathological response; PBMC, peripheral blood mononuclear cell.

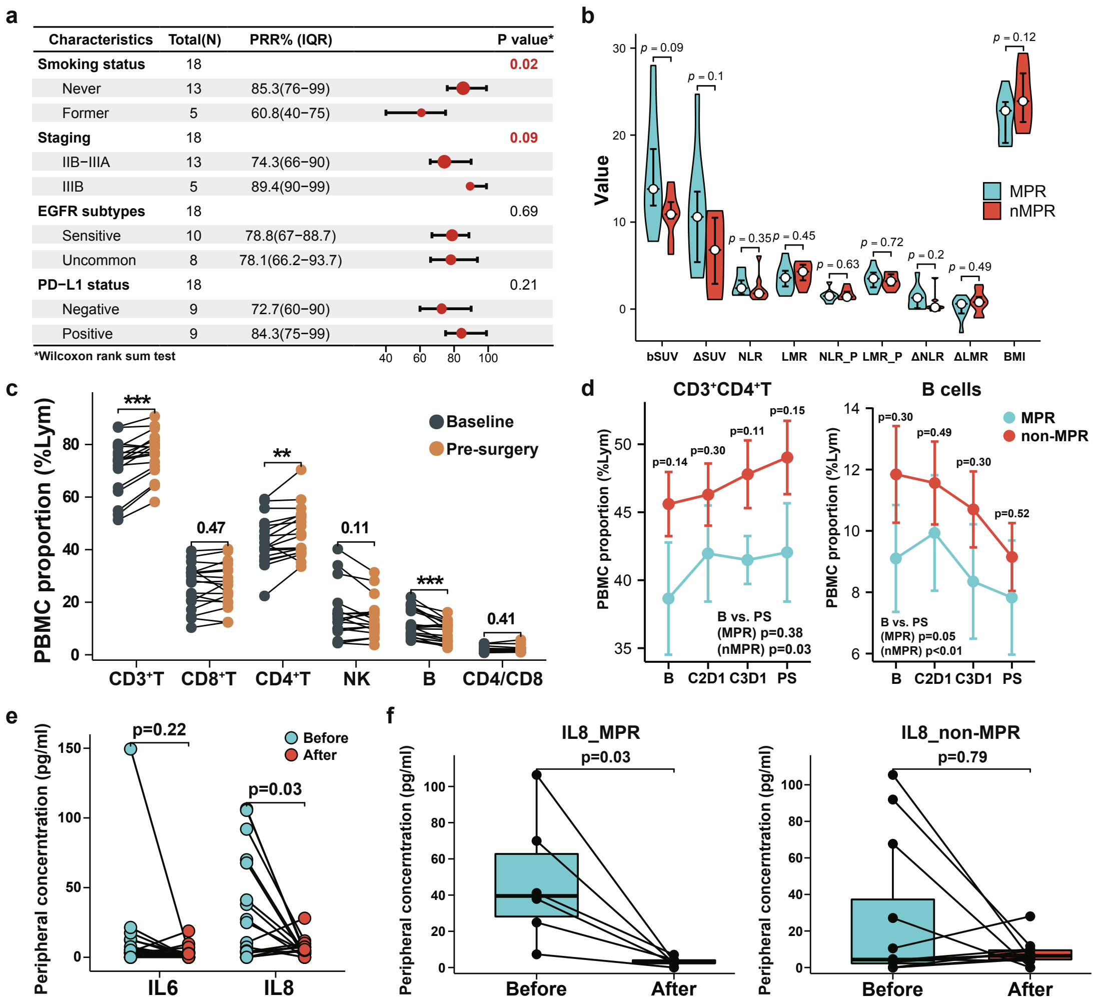

Supplementary Fig.4 Correlation of genomic features and pathological response, related to Figure 3 and STAR Methods. a Comparison of TP53 subtypes between MPR and non-MPR patients. Fisher exact test was used to measure the significance. **b** Relative abundance of preferable altered mutations in MPR or non-MPR patients with annotated genes correlated with TKIs (red) or immune response (green). **c** Pathway enrichment of MPR and non-MPR patients through differential genes. MPR, major pathological response; TKI, tyrosine kinase inhibitor.

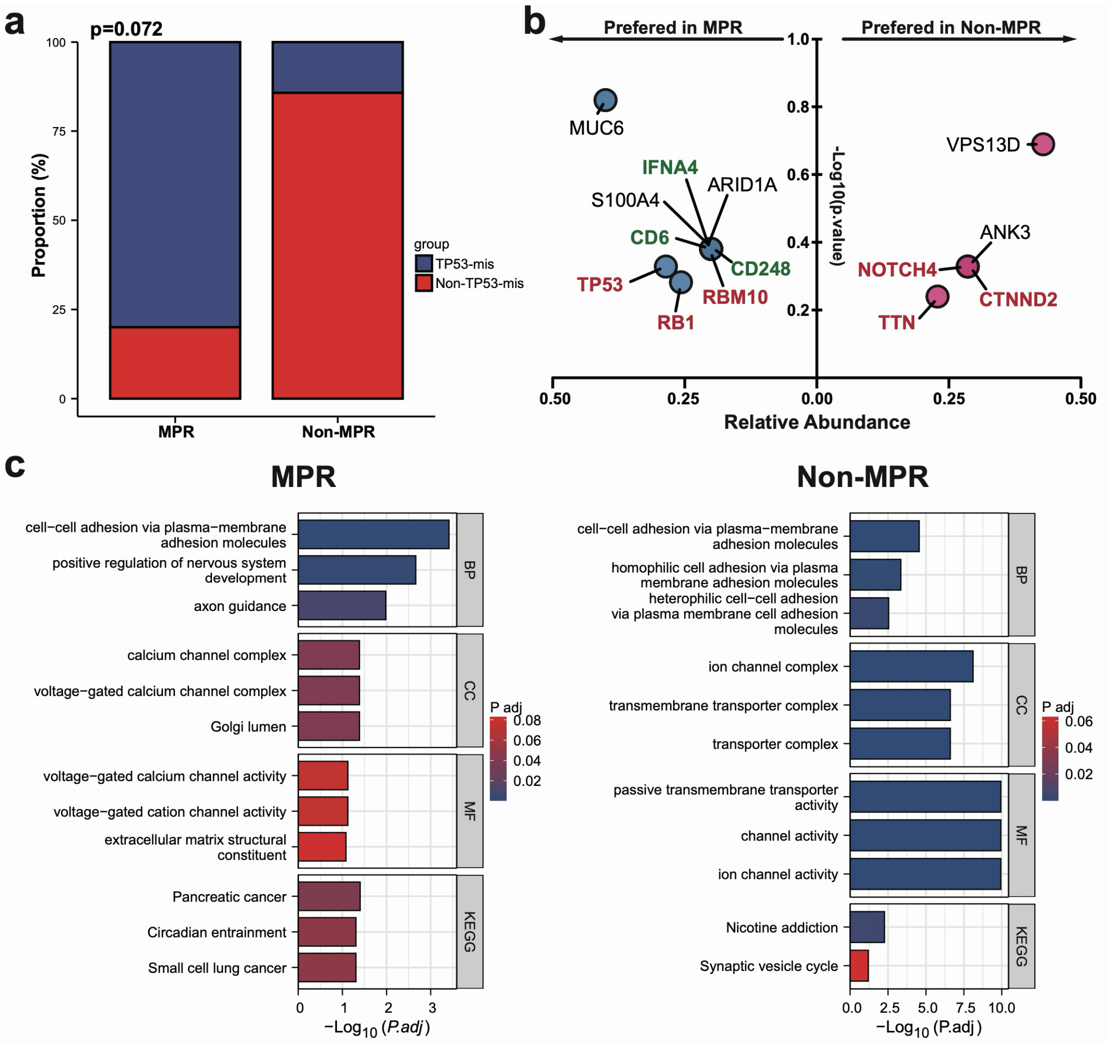

Supplementary Fig.5 Establishment of PDOs for residual tumor and susceptibility test, related to Figure 3 and STAR Methods. a Verification of PDOs through HE and IHC staining for two patients. Staining from both origin tumor and PDOs were matched. **b** Comparison of genomic features showed relatively similar CNVs between origin tumor and PDOs as well as specific EGFR mutation subtypes. **c** Anti-tumor susceptibility test suggested superior efficacy of second-generation EGFR-TKIs followed by the third generation EGFR-TKI. IC50 of each drug was presented along with AUC value. PDOs, patient-derived organoids; HE, hematoxylin-eosin; IHC, immunohistochemistry; IC50, half maximal inhibitory concentration.

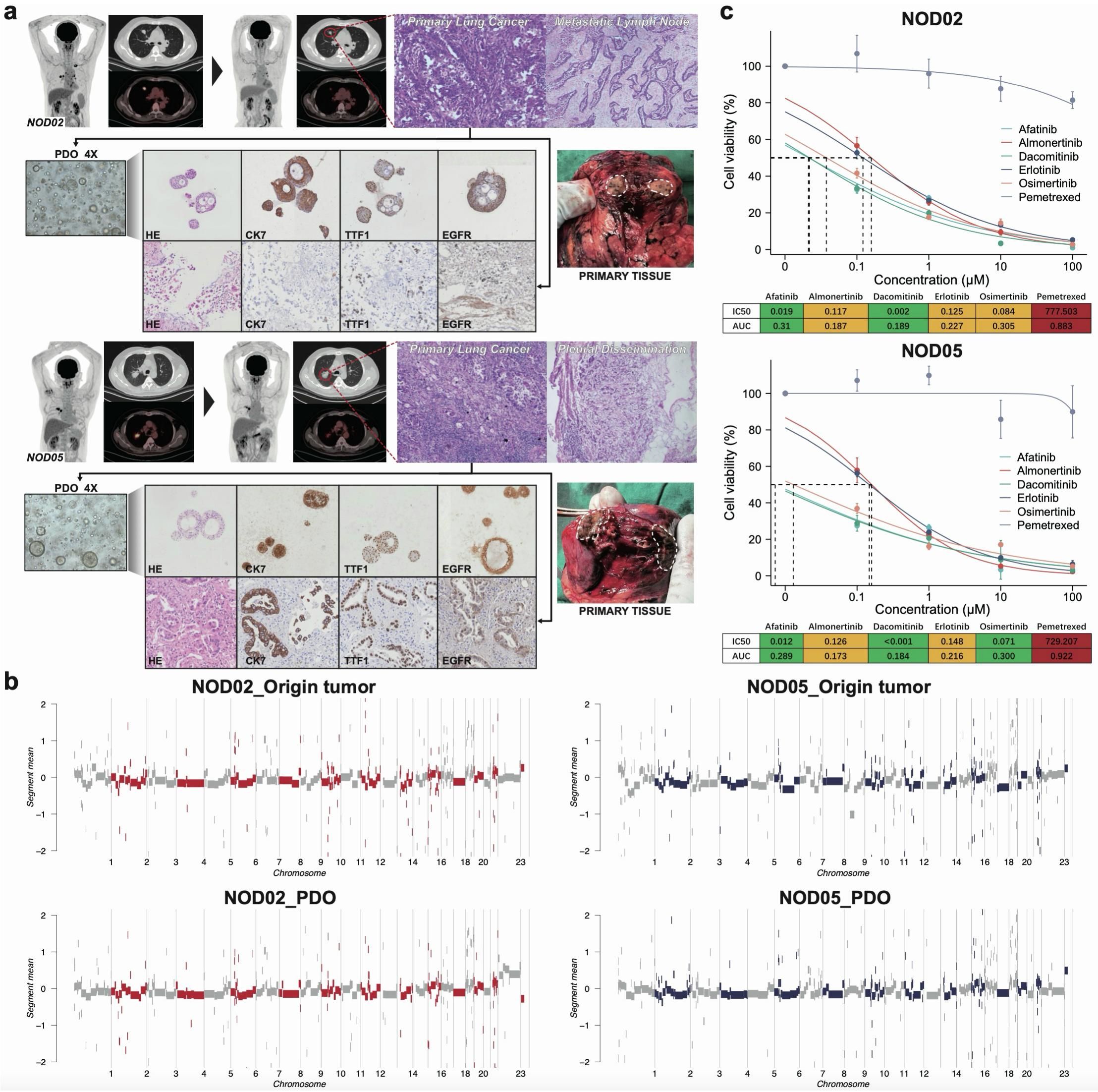

Supplementary Fig.6 Other immune-relevant genomic features regarding WES, related to Figure 3 and STAR Methods. Comparison of WGD(**a**), Ploidy(**b**), HLA-LOH(**c**), TMB(**d**), TNB(**e**) and CIN(**f**) between MPR and non-MPR patients (N=12). Fisher test was used to calculate the significance for categorical variables and T test was used for continuous variable. **g** Immune selection measured through SOPRANO algorithm among different individuals. No apparent difference was found between groups. WGD, whole genome doubling; HLA-LOH, human leukocyte antigen loss of heterozygosity; TMB, tumor mutation burden; TNB. Tumor neoantigen burden; CIN, chromosomal instability; dN/dS, nonsynonymous to synonymous mutations ratio.

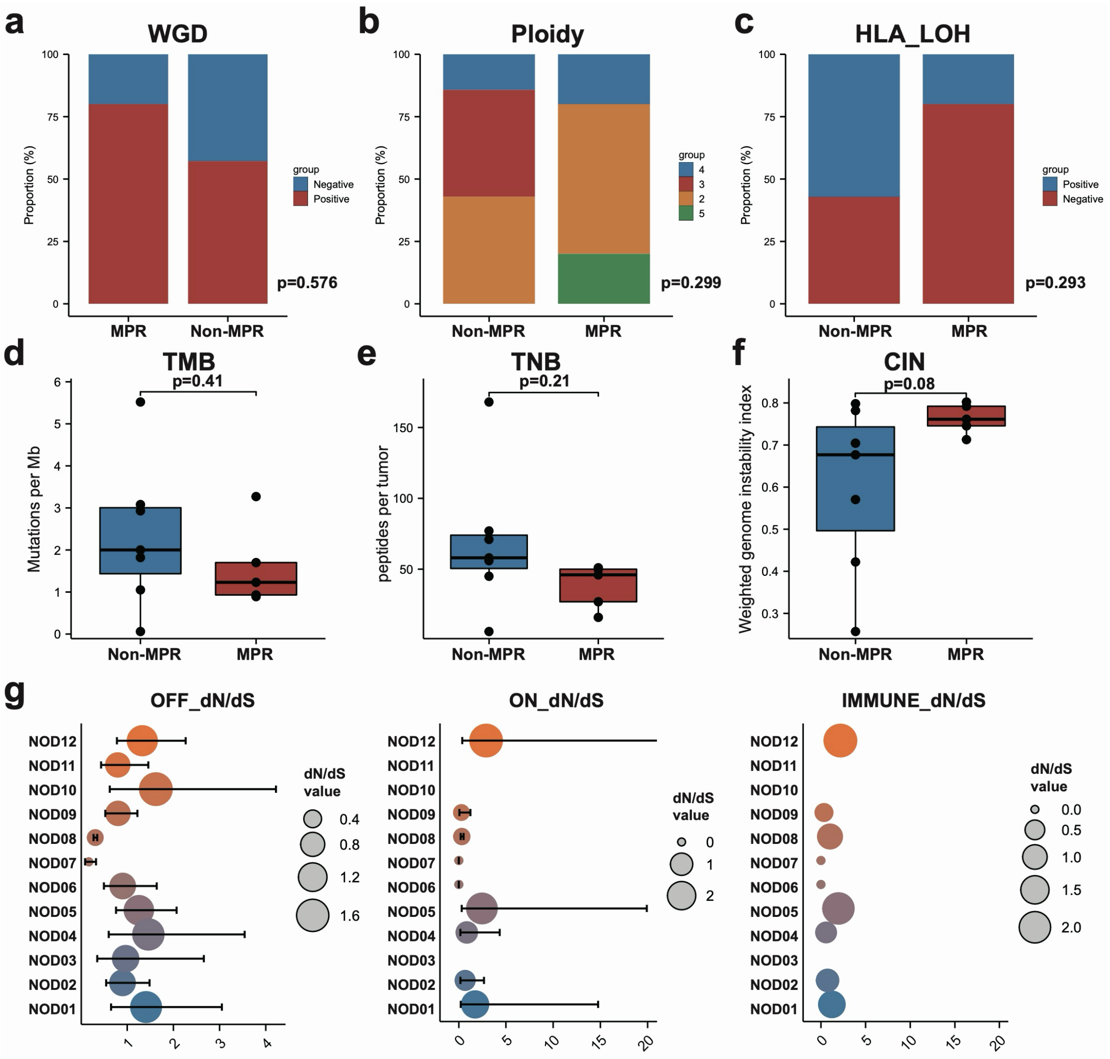

Supplementary Fig.7 Flowchart of ctDNA acquisition and dynamic changes, related to Figure 3 and STAR Methods. a Acquisition of ctDNA in first 12 patients. 2 patients did not have adequate tumor for WES and used resected specimens to generate the customized ctDNA panel. **b** Positivity of all customized ctDNA levels and EGFR mutation only at baseline detected through tumor-informed MRD. EGFR KDD mutation derived from one patient could not be captured by WES technically, but large panel NGS confirmed EGFR KDD mutation in resected specimens after neoadjuvant treatment. **c** Percentage of ctDNA positivity across different time points. **d** Dynamic changes of ctDNA status and response outcome. **e** Comparison of MTM between MPR and non-MPR patients across different time points. Welch t' test was used to calculate the significance.

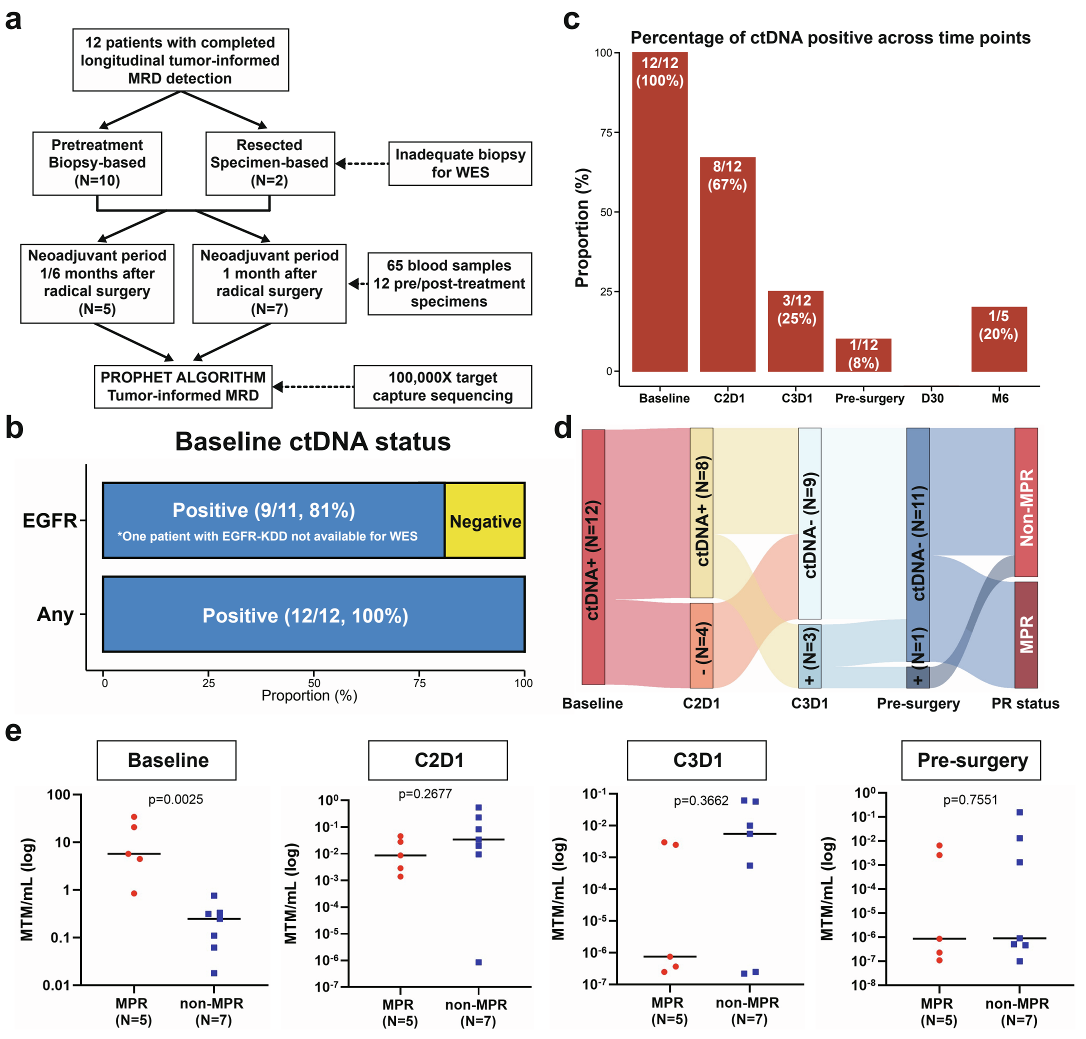

Supplementary Fig.8 Longitudinal monitoring of informed MRD detection, related to Figure 3 and STAR Methods. Each plot represented longitudinal changes of MRD individually along with their genomics features. LUAD, lung adenocarcinoma; LUASC, lung adeno-squamous carcinoma; EGFR, epidermal growth factor receptor; MRD, minimal residual disease; WGD, whole genome doubling; PL, ploidy; IMS, immune status; HLA-LOH, human leukocyte antigen loss of heterozygosity; TMB, tumor mutation burden; TNB, tumor neoantigen burden.

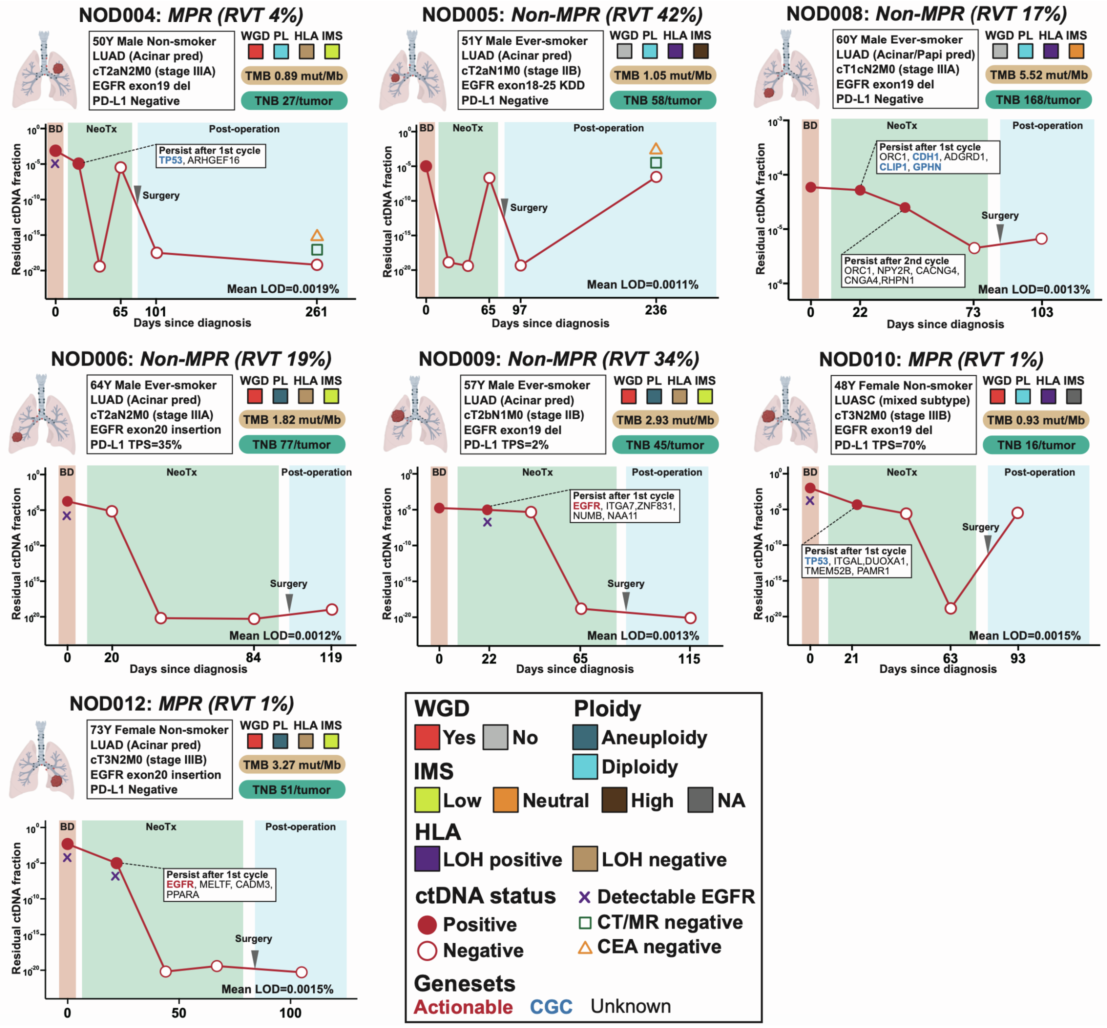

Supplementary Fig.9 Patients' characteristics of included cohorts and quality control of single-cell sequencing, related to Figure 4 and STAR Methods. a Baseline clinical features of NEOTIDE and real-world cohort. Only one patient from NEOTIDE cohort did not have qualified resected sample for single-cell sequencing while others all have available samples for single-cell RNA sequencing. **b** Quality control of single-cell RNA data in NEOTIDE and real-world cohort including cell numbers and total counts per cell. **c** Clustering of major cell type lineage and comparison of different cell types regarding MPR status. T test was used to measure the significance.

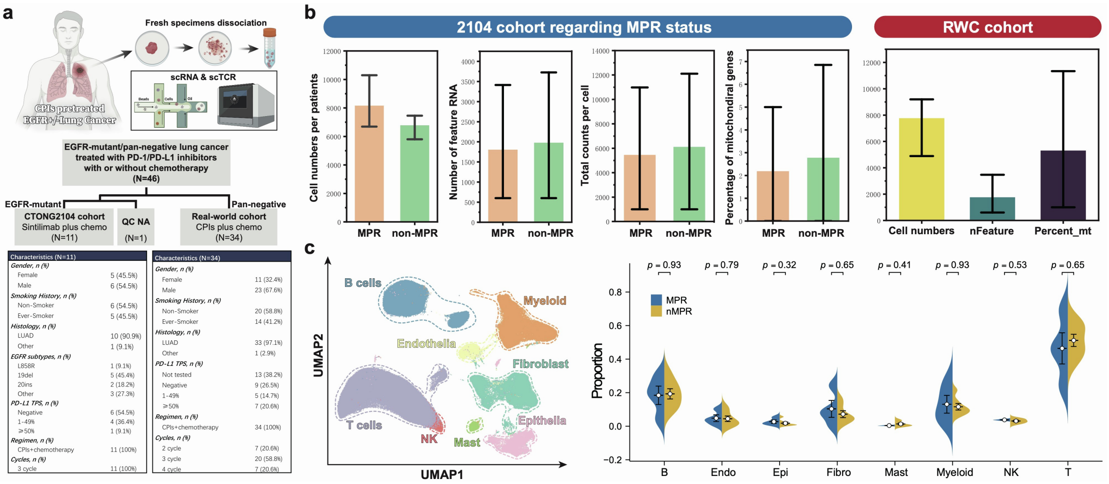

Supplementary Fig.10 Cell subsets annotation through specific feature genes, related to Figure 4, Figure 5 and STAR Methods. Bubble plots were generated to annotate different cell subtypes within diverse major lineage including CD4+lymphocytes (**a**), CD8+lymphocytes (**b**), B cells (**c**), myeloid cells (**d**) and innate immune cells (**e**).

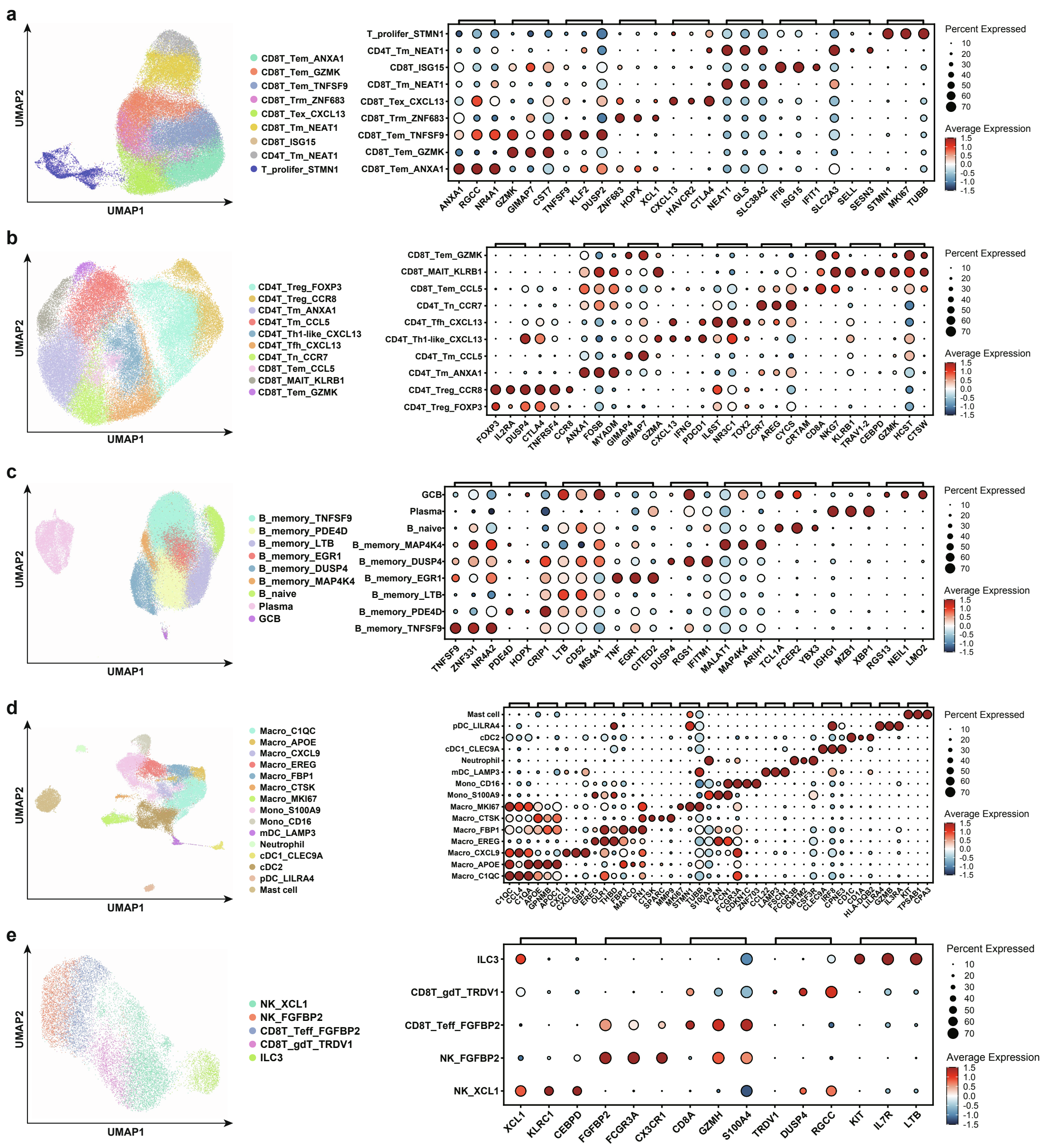

Supplementary Fig.11 Multiple immunohistochemistry staining of pre- and post-treatment specimens in responsive patients, related to Figure 5 and STAR Methods. HE, CD4, CD8 and Foxp3 staining was performed. Red dashed lines indicated tumor area and green arrow indicated staining cells.

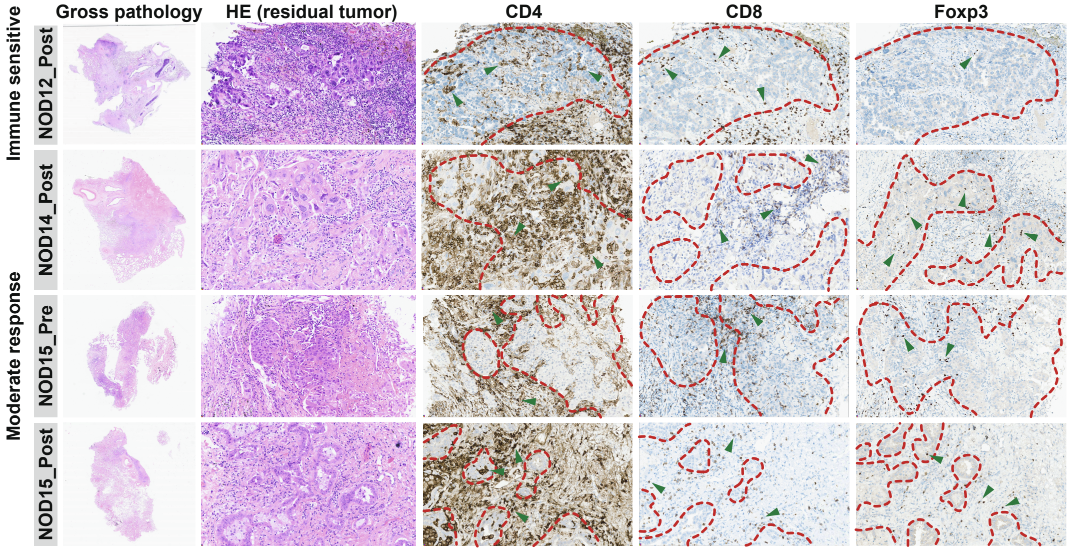

Supplementary Fig.12 Correlation of PD-L1 status and CCR8+Treg/CXCL13+Tex signature, related to Figure 5 and STAR Methods. Proportion of diverse PD-L1 status among different CCR8+Treg/CXCL13+Tex signature. Yates' correction was used to measure the significance.

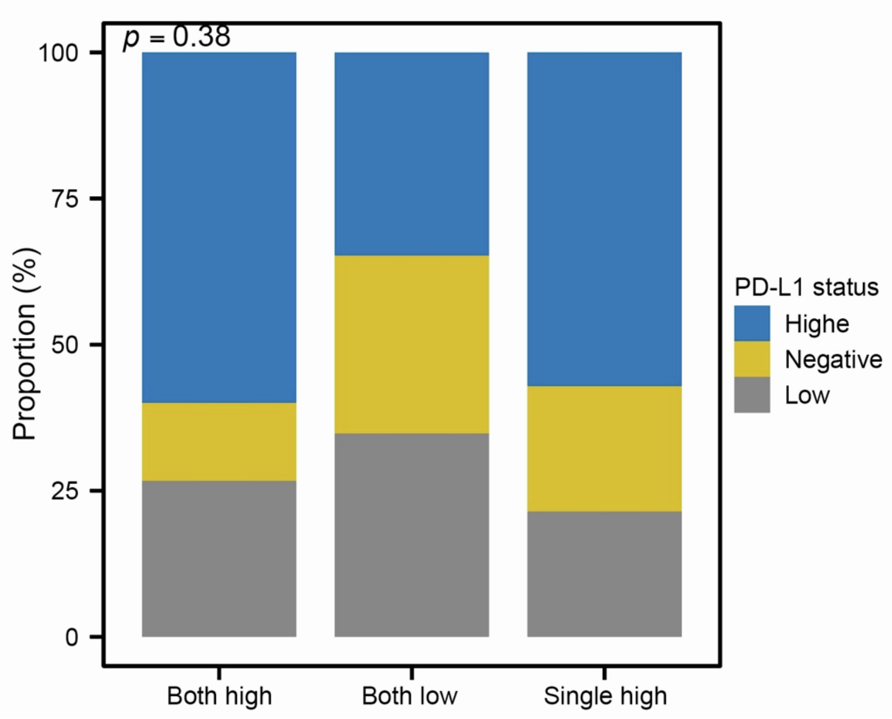

Supplementary Fig.13 External validation through public immunotherapy cohorts, related to Figure 5 and STAR Methods. a Response rate and overall survival of PD-L1 treated NSCLC cohort (Gainor et al). Stratified immune phenotypes were assessed through predominantly pre-treatment samples. Yates' correction was used to calculate the significance among groups. Log-rank p-value was conducted for survival analysis. **b** Response rate and progression-free survival of PD-1 treated melanoma cohort (Schadendorf et al). Stratified immune phenotypes were assessed through predominantly on-treatment samples. Yates' correction was used to calculate the significance among groups. Log-rank p-value was conducted for survival analysis. **c** Response rate and overall survival of PD-1 treated melanoma cohort (Chan et al). Stratified immune phenotypes were assessed through predominantly on-treatment samples. Chisq test was used to calculate the significance between groups. Log-rank p-value was conducted for survival analysis. **d** Proposed predictive value of CCR8^{+}Treg and CXCL13^{+}Tex before and after neoadjuvant immunotherapy in wild-type and EGFR-mutant NSCLC. PD, progressive disease; SD, stable disease; PR, partial response; CR, complete response; DCR, disease control rate; PD-1/PD-L1, PD-L1, programmed cell death protein 1/programmed cell death protein ligand-1; PFS, progression-free survival; OS, overall survival.

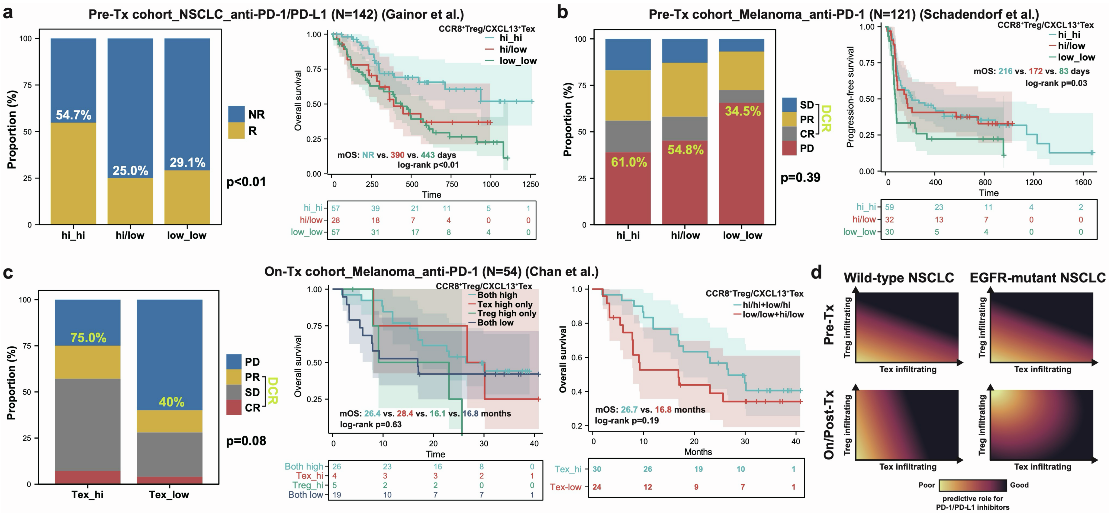

Supplementary Fig.14 Comparison of myeloid subtypes regarding response patterns, related to Figure 6. Proportion of different myeloid subtypes were compared in boxplot regarding response patterns. T test was used to measure the significance.

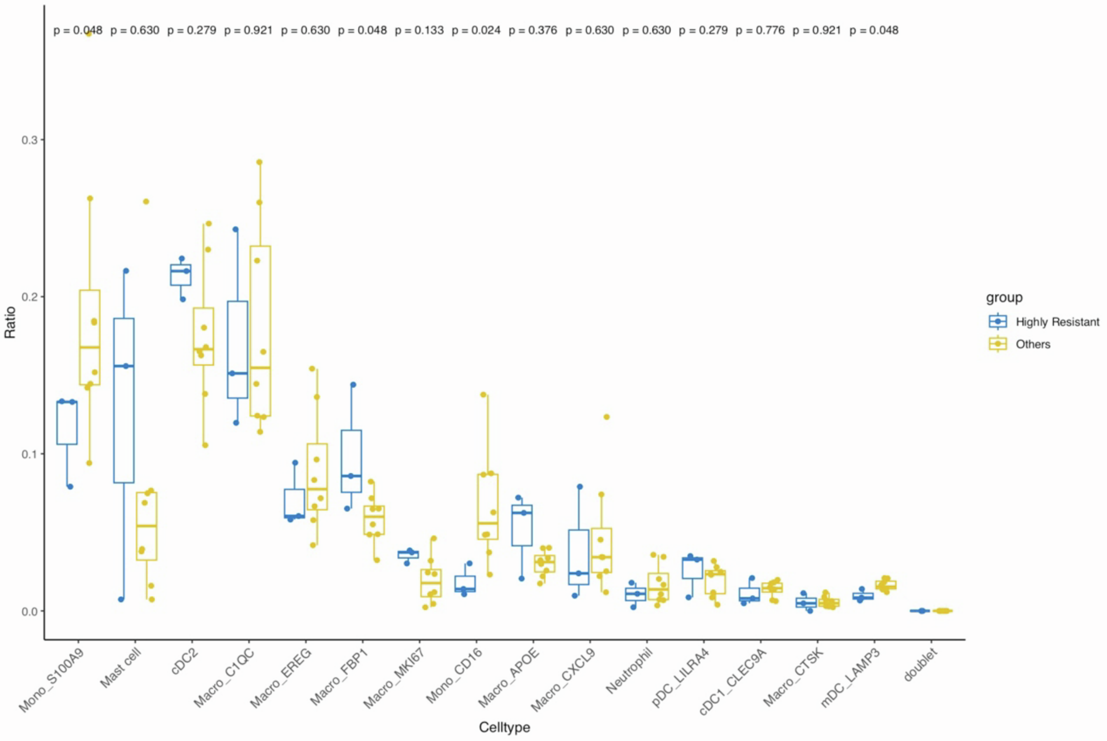

Supplement: Document S1. Figures S1–S14 [file mmc1.pdf]
