# Supplementary material for: Neoadjuvant sintilimab plus chemotherapy in EGFR-mutant NSCLC: Phase 2 trial interim results (NEOTIDE/CTONG2104)
Source: Cell Rep Med. 2024 Jun 18;5(7):101615. doi: 10.1016/j.xcrm.2024.101615 (PMC11293361; doi:10.1016/j.xcrm.2024.101615)

Cell Reports
Medicine

Article

Neoadjuvant sintilimab plus chemotherapy in EGFR-mutant NSCLC: Phase 2 trial interim results (NEOTIDE/CTONG2104)

Graphical abstract

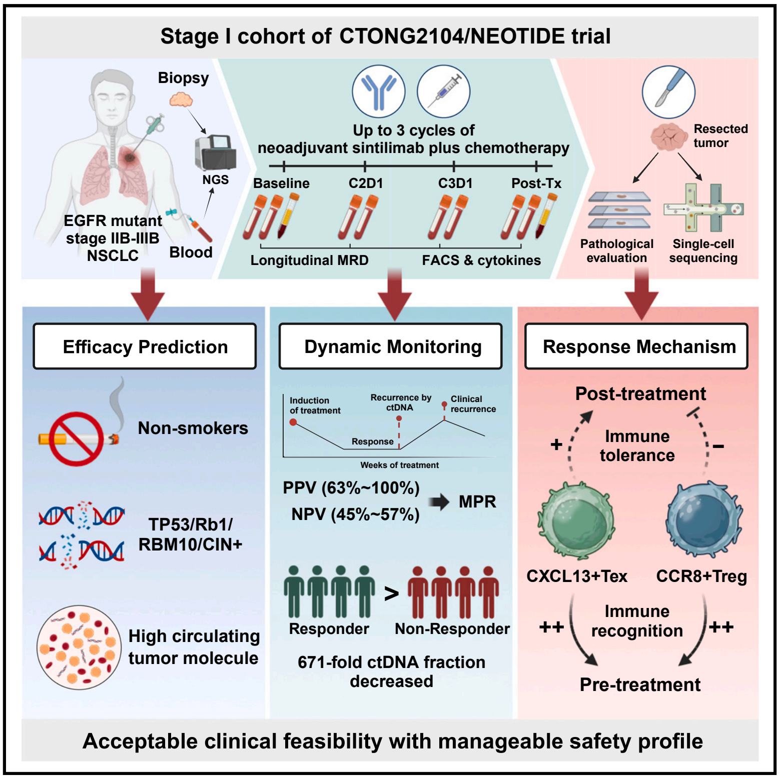

Highlights

- Neoadjuvant sintilimab and chemotherapy are clinically feasible in EGFR-mutant NSCLC
- The treatment regimen is well tolerated, with comparable surgical outcome
- Baseline genomic features and dynamic MRD help identify potential beneficiaries
- CXCL13^{+}Tex/CCR8^{+}Treg cell phenotype predicts efficacy of pre- and post-immunotherapy

Authors

Chao Zhang, Yu-Xuan Sun, Ding-Cheng Yi, ..., Yi-Long Wu, Ze-Min Zhang, Wen-Zhao Zhong

Correspondence

syzhongwenzhao@scut.edu.cn

In brief

Zhang et al. demonstrate the acceptable clinical feasibility and safety of neoadjuvant sintilimab and chemotherapy in patients with EGFR-mutant localized NSCLC. They highlight that the baseline genomic features and dynamic MRD detection help identify responders and illustrate that the CXCL13^{+} Tex/CCR8^{+} Treg cell infiltrating phenotype is correlated with diverse immune statuses and clinical efficacy of immunotherapy.

Zhang et al., 2024, Cell Reports Medicine 5, 101615
July 16, 2024 © 2024 The Author(s). Published by Elsevier Inc.
https://doi.org/10.1016/j.xcrm.2024.101615

CellPress

Cell Reports Medicine

CellPress
OPEN ACCESS

Article

Neoadjuvant sintilimab plus chemotherapy in EGFR-mutant NSCLC: Phase 2 trial interim results (NEOTIDE/CTONG2104)

Chao Zhang,[1,2,11,12] Yu-Xuan Sun,[3,12] Ding-Cheng Yi,[3,12] Ben-Yuan Jiang,[1,2,12] Li-Xu Yan,[4,12] Ze-Dao Liu,[3] Li-Shan Peng,[1,2] Wen-Jie Zhang,[3] Hao Sun,[2] Zhi-Yong Chen,[2,5] Dan-Hua Wang,[6] Di Peng,[6] Song-An Chen,[6] Si-Qi Li,[7] Ze Zhang,[8] Xiao-Yue Tan,[9] Jie Yang,[1,2] Zhang-Yi Zhao,[3] Wan-Ting Zhang,[3] Jian Su,[2] Yang-Si Li,[2] Ri-Qiang Liao,[1,2] Song Dong,[1,2] Chong-Rui Xu,[2] Qing Zhou,[2] Xue-Ning Yang,[1,2] Yi-Long Wu,[2] Ze-Min Zhang,[3,10] and Wen-Zhao Zhong[1,2,13,*]

[1]Department of Pulmonary Surgery, Guangdong Lung Cancer Institute, Guangdong Provincial People's Hospital (Guangdong Academy of Medical Sciences), Southern Medical University, Guangzhou, China
[2]Guangdong Lung Cancer Institute, Guangdong Provincial People's Hospital (Guangdong Academy of Medical Sciences), Southern Medical University, Guangzhou, China
[3]School of Life Sciences, Peking University, Beijing, China
[4]Department of Pathology, Guangdong Provincial People's Hospital (Guangdong Academy of Medical Sciences), Southern Medical University, Guangzhou, China
[5]Department of Radiation Therapy, Guangdong Provincial People's Hospital (Guangdong Academy of Medical Sciences), Southern Medical University, Guangzhou, China
[6]Burning Rock Biotech, Guangzhou, China
[7]Guangzhou Institutes of Biomedicine and Health, Chinese Academy of Sciences, Guangzhou, China
[8]Institute of Biomedical Research, Yunnan University, Kunming, China
[9]PET Center, Department of Nuclear Medicine, Guangdong Provincial People's Hospital (Guangdong Academy of Medical Sciences), Southern Medical University, Guangzhou, China
[10]BIOPIC, Beijing Advanced Innovation Center for Genomics, Peking University, Beijing, China
[11]School of Medicine, South China University of Technology, Guangzhou, China
[12]These authors contributed equally
[13]Lead contact
*Correspondence: syzhongwenzhao@scut.edu.cn
https://doi.org/10.1016/j.xcrm.2024.101615

SUMMARY

The clinical efficacy of neoadjuvant immunotherapy plus chemotherapy remains elusive in localized epidermal growth factor receptor (EGFR)-mutant non-small cell lung cancer (NSCLC). Here, we report interim results of a Simon's two-stage design, phase 2 trial using neoadjuvant sintilimab with carboplatin and nab-paclitaxel in resectable EGFR-mutant NSCLC. All 18 patients undergo radical surgery, with one patient experiencing surgery delay. Fourteen patients exhibit confirmed radiological response, with 44% achieving major pathological response (MPR) and no pathological complete response (pCR). Similar genomic alterations are observed before and after treatment without influencing the efficacy of subsequent EGFR-tyrosine kinase inhibitors (TKIs) *in vitro*. Infiltration and T cell receptor (TCR) clonal expansion of CCR8^{+} regulatory T (Treg)^{hi}/CXCL13^{+} exhausted T (Tex)^{lo} cells define a subtype of EGFR-mutant NSCLC highly resistant to immunotherapy, with the phenotype potentially serving as a promising signature to predict immunotherapy efficacy. Informed circulating tumor DNA (ctDNA) detection in EGFR-mutant NSCLC could help identify patients nonresponsive to neoadjuvant immunochemotherapy. These findings provide supportive data for the utilization of neoadjuvant immunochemotherapy and insight into immune resistance in EGFR-mutant NSCLC.

INTRODUCTION

Epidermal growth factor receptor (EGFR) is the first identified actionable target for non-small cell lung cancer (NSCLC),[1] representing up to 50% of advanced NSCLC cases in Asia.[2] Multiple trials have consistently shown the superior efficacy of EGFR-tyrosine kinase inhibitors (TKIs) in advanced NSCLC patients with sensitive EGFR mutations.[3–5] Nevertheless, treatment resistance eventually emerges in most patients, suggesting that the predominant role of TKIs in lung cancer might be to delay cancer proliferation and metastasis rather than to eradicate the disease.[6–8] Similar observations have been made in both adjuvant and neoadjuvant settings for early-stage EGFR-mutant NSCLC. Trials such as ADJUVANT (ClinicalTrials.gov: NCT01405079)[9] and IMPACT (UMIN000006252),[10] which employed first-generation EGFR-TKIs as adjuvant treatments,

© 2024 The Author(s). Published by Elsevier Inc.
This is an open access article under the CC BY license (http://creativecommons.org/licenses/by/4.0/).

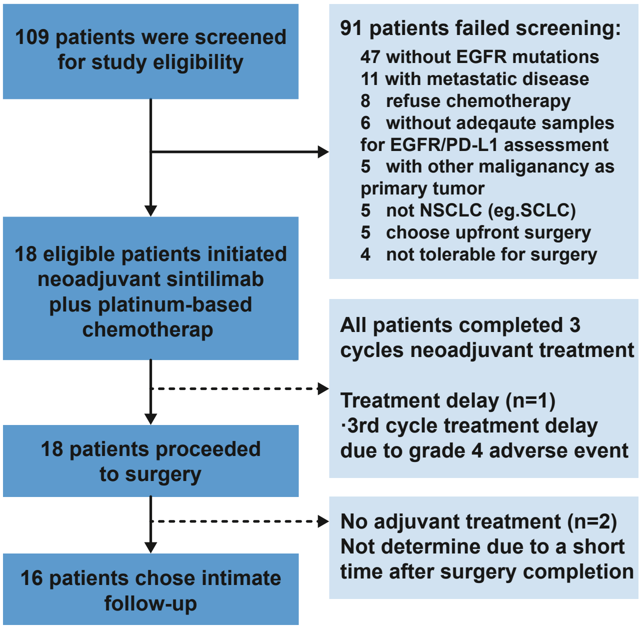

Figure 1. Consort diagram and treatment disposition
109 patients in total were screened for study eligibility, and 91 of them did not meet the inclusion criteria due to absence of EGFR mutations, confirmed metastatic disease, etc. 18 enrolled patients had completed three cycles of neoadjuvant sintilimab plus chemotherapy and received radical surgery. 16 patients chose intimate follow-up without adjuvant targeted therapy, while the other two patients did not determine postoperative treatment at data cutoff.

failed to display significantly improved overall survival (OS), although an elevated disease-free survival was noted in ADJUVANT. In the ADAURA study (NCT02511106), a significant improvement in OS was observed with adjuvant osimertinib. However, disease relapse accelerated after 3 years of adjuvant treatment, indicating the non-curative nature of EGFR-TKIs in the adjuvant setting.[11] Additionally, multiple phase 2 studies have indicated that EGFR-TKIs might not be an effective strategy for the neoadjuvant setting, with MPR ranging from 10%–15%.[12,13]

Unlike TKIs, which primarily target tumor cells with specific driver mutations by inhibiting cell proliferation and invasion,[1] immunotherapy offers an encouraging anti-tumor strategy. This approach enhances the ability of infiltrating T cells to recognize tumor cells by inhibiting the programmed cell death protein-1 (PD-1)/programmed cell death protein ligand-1 (PD-L1) axis.[14,15] Mounting evidence supports the long-term survival benefits of immunotherapy in advanced NSCLC,[16] reflecting an improved prognosis with an impressive 5-year survival rate of up to 31.9%.[17] Given the curative potential of immunotherapy, combining checkpoint inhibitors with surgical resection, another curative method for localized NSCLC, presents a compelling avenue for further research. The Checkmate-816 study (ClinicalTrials.gov: NCT02998528) was pioneering in demonstrating a significantly elevated pathological response rate for neoadjuvant immunotherapy combined with chemotherapy in localized NSCLC compared to traditional neoadjuvant chemotherapy.[18] Subsequent analyses of surgical outcomes further affirmed the clinical viability of pairing neoadjuvant immunotherapy with chemotherapy in multiple prospective trials.[19] However, most of these trials excluded patients with either EGFR or anaplastic lymphoma kinase (ALK) mutations, given the limited efficacy of immunotherapy observed in advanced NSCLC carrying these driver mutations. In a phase 2 trial that assessed neoadjuvant atezolizumab combined with doublet chemotherapy, a promising pathological response was observed in a subset of EGFR-mutant NSCLC patients.[20] Yet, the limited sample size might introduce potential biases to these findings. In the context of advanced NSCLC, single-agent immunotherapy did not exhibit clinical activity in either first-line (ClinicalTrials.gov: NCT02879994)[21] or subsequent treatments.[22] Nonetheless, the IMpower150 trial was the first to demonstrate the moderate success of a combined immunotherapy strategy for advanced EGFR-mutant NSCLC.[23] Building on the post hoc data from IMpower150, a phase 2 trial named Neo-DIANA (ClinicalTrials.gov: NCT04512430) was launched to evaluate neoadjuvant atezolizumab combined with platinum-based doublet chemotherapy and bevacizumab in EGFR-mutant NSCLC patients. However, it is imperative to recognize that adding bevacizumab might introduce challenges in surgical resection due to potential complications or might even reduce resection rates owing to more severe adverse events.

Our group previously published a retrospective multicenter study of patients harboring known driver mutations treated with neoadjuvant immunotherapy plus chemotherapy.[24] In that study, we indirectly compared clinical efficacy among EGFR-mutant patients treated with different neoadjuvant regimens, and immunotherapy plus chemotherapy yielded much better efficacy compared to erlotinib or chemotherapy only. Therefore, we launched an investigator-initiated, Simon's two-stage design trial of neoadjuvant sintilimab combined with carboplatin and nab-paclitaxel (nab-PC) in early-stage EGFR-mutant NSCLC (ClinicalTrials.gov: NCT05244213). Here we report the interim results of stage 1 cohort and multiomics profiling of neoadjuvant immunotherapy combination in early-stage EGFR-mutant patients (Figure S1).

RESULTS

Patient baseline characteristics and treatment disposition

Between May 10, 2022, and May 25, 2023, 109 patients were screened and 18 of them who passed screening and consented were treated at Guangdong Lung Cancer Institute. The most common reasons for screen failure included no known EGFR mutations (52%) and metastatic disease (12%) (Figure 1). The median age of treated patients was 59 (range 48–73), and 44% of patients were male. 72% patients were staged at baseline through positron emission tomography (PET)/computed tomography (CT) plus enhanced brain MRI and only PET/CT for others. Baseline clinical stage was 17% IIB, 55% IIIA, and 28% IIIB by the American Joint Committee on Cancer, 8th edition criteria. 11 of 14 (79%) cN2 patients were pathologically confirmed, and the other two were not available for biopsy (stations 5 and 6). All enrolled patients had confirmed EGFR driver mutations including 5 EGFR 19Del, 5 L858R,

Table 1. Patient characteristics regarding pathological response

Characteristics	All N = 18	MPR N = 8	nMPR N = 10	p Value	Statistic
Age, mean ± SD	58.9 ± 6.9	57.3 ± 9.1	60.2 ± 4.5	0.42	Welch's t test
Gender, n (%)	–	–	–	0.02	Fisher's test
Male	8 (44.4%)	1 (12.5%)	7 (70.0%)	–	–
Female	10 (55.6%)	7 (87.5%)	3 (30.0%)	–	–
Smoking, n (%)	–	–	–	0.04	Fisher's test
Non-smoker	13 (72.2%)	8 (100.0%)	5 (50.0%)	–	–
Ever-smoker	5 (27.8%)	0 (0%)	5 (50.0%)	–	–
PS, n (%)	–	–	–	0.12	Fisher's test
0	13 (72.2%)	4 (50.0%)	9 (90.0%)	–	–
1	5 (27.8%)	4 (50.0%)	1 (10.0%)	–	–
Baseline staging	–	–	–	0.18	Fisher's test
PET/CT + MRI	13 (72.2%)	5 (62.5%)	8 (80.0%)	–	–
PET/CT	5 (27.8%)	3 (37.5%)	2 (20.0%)	–	–
Histology, n (%)	–	–	–	0.18	Fisher's test
Adenocarcinoma	16 (88.9%)	6 (75.0%)	10 (100.0%)	–	–
Adeno-squamous	2 (11.1%)	2 (25.0%)	0 (0%)	–	–
EGFR, n (%)	–	–	–	0.33	Fisher's test
EGFR 19del	5 (27.8%)	2 (25.0%)	3 (30.0%)	–	–
EGFR 21L858R	5 (27.8%)	1 (12.5%)	4 (40.0%)	–	–
EGFR 20ins	4 (22.2%)	3 (37.5%)	1 (10.0%)	–	–
Uncommon	4 (22.2%)	2 (25.0%)	2 (20.0%)	–	–
T stage, n (%)	–	–	–	0.06	Fisher's test
1	4 (22.2%)	0 (0%)	4 (40.0%)	–	–
2	8 (44.4%)	4 (50.0%)	4 (40.0%)	–	–
3	3 (16.7%)	3 (37.5%)	0 (0%)	–	–
4	3 (16.7%)	1 (12.5%)	2 (20.0%)	–	–
N stage, n (%)	–	–	–	0.59	Fisher's test
1	4 (22.2%)	1 (12.5%)	3 (30.0%)	–	–
2	14 (77.8%)	7 (87.5%)	7 (70.0%)	–	–
TNM, n (%)	–	–	–	0.19	Fisher's test
IIB	3 (16.7%)	1 (12.5%)	2 (20.0%)	–	–
IIIA	10 (55.6%)	3 (37.5%)	7 (70.0%)	–	–
IIIB	5 (27.8%)	4 (50.0%)	1 (10.0%)	–	–
PD-L1, n (%)	–	–	–	0.83	Fisher's test
Negative	9 (50%)	4 (50.0%)	5 (50.0%)	–	–
1%–49%	6 (33.3%)	2 (25.0%)	4 (40.0%)	–	–
≥50%	3 (16.7%)	2 (25.0%)	1 (10.0%)	–	–

MPR, major pathological response; PET, positron emission tomography; PS, physical score; EGFR, epidermal growth factor receptor; PD-L1, programmed cell death ligand 1.

4 exon 20 insertion, and 4 other rare driver mutations (G719X and kinase domain duplication). 50% of the treated patients had negative PD-L1 (Dako 22c3) expression, and 33% had low (1%–49%) and 17% had high PD-L1 expression. Detailed baseline characteristics are listed in Tables 1 and S1. We collected biopsy and resected tissue samples and serial peripheral blood during the perioperative period and performed whole-exome sequencing (WES), bulk RNA-sequencing (RNA-seq), and single-cell RNA-seq/T cell receptor (TCR) sequencing and illustrated the association with pathological response (Figure S1; Table S2).

All 18 patients completed 3 cycles of neoadjuvant sintilimab and platinum-based chemotherapy. Four patients (22%) experienced a dose reduction (−20%) owing to grade 3 or 4 myelosuppression after the first or second cycle of neoadjuvant treatment. All patients were determined to be available for surgery after evaluation by thoracic surgeons and radiation oncologist. 17 patients proceeded to surgery as scheduled within 3–6 weeks after

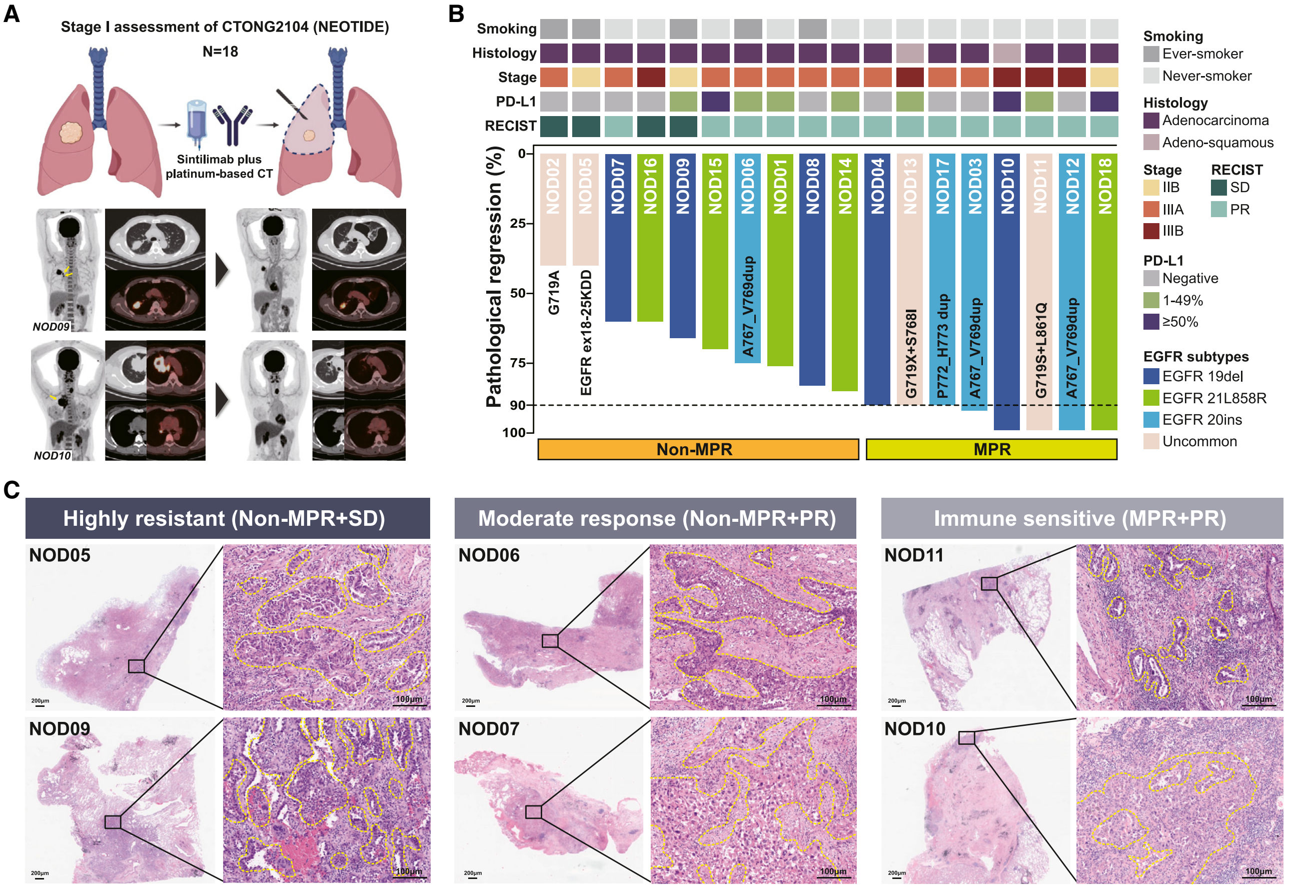

Figure 2. Clinicopathological features and preliminary response

(A) Study treatment and representative radiological response before and after neoadjuvant sintilimab plus nab-PC through PET-CT.

(B) Individual clinicopathological features and pathological response. The black horizontal line indicates the threshold for MPR patients.

(C) Representative pathological response and stratified response patterns in terms of radiological and pathological response, including the highly resistant group (*n* = 2), moderate response group (*n* = 2), and immune-sensitive group (*n* = 2). The yellow dotted lines circle the tumor area. PET-CT, positron emission tomography and computed tomography; MPR, major pathological response; PR, partial response; PD-L1, programmed cell death protein ligand-1; EGFR, epidermal growth factor receptor.

the last dose of neoadjuvant treatment. One patient had delayed surgery due to spontaneous pneumothorax, which was irrelevant to the study drugs. All enrolled patients had completed surgical resection, and 16 patients chose intimate follow-up, while the last two patients were not determined at data cutoff (Figure 1).

Clinical activity and perioperative safety

Among the 18 patients who completed neoadjuvant treatment and underwent surgery, 14 (78%) achieved partial response (PR), and 4 (22%) had SD with no radiological progressive disease regarding pre- and post-treatment PET/CT. For patients who underwent surgery, 8 patients (44%) achieved major pathological response (MPR), and 4 of them had near-pathological complete response (pCR) with 1% residual viable tumor (RVT). No pCR was observed in the stage 1 cohort. Specifically, patients with sensitive EGFR mutations, exon 20 insertion, and other uncommon EGFR mutations achieved 30% (3 of 10), 75% (3 of 4), and 50% (2 of 4) MPR, respectively. 56% (10 of 18) patients had confirmed pathological downstaging (Figures 2A and 2B; Table S3). Employing a meticulous pathological evaluation process (as detailed in Figure S2), we categorized the patients into three groups based on radiological and pathological responses: those highly resistant (showing resistance to both chemotherapy and immunotherapy), those with a moderate response (displaying only a radiological response), and those deemed immune sensitive (exhibiting a profound pathological response). This categorization aimed to facilitate subsequent exploratory analyses (Figure 2C). Interestingly, most clinicopathological features, including EGFR subtypes and PD-L1 expression, did not exhibit significant differences between the MPR and non-MPR (nMPR) groups. However, the MPR group had a notably higher tumor burden and consisted predominantly of non-smokers and female patients (Table 1; Figure S3A). Furthermore, there were no discernible differences between the groups in terms of pre- and post-treatment standardized uptake value, lymphoid-neutrophil ratio, myeloid-lymphoid ratio, and BMI (Figure S3B). Dynamic monitoring of flow cytometry results for peripheral blood

CellPress
OPEN ACCESS

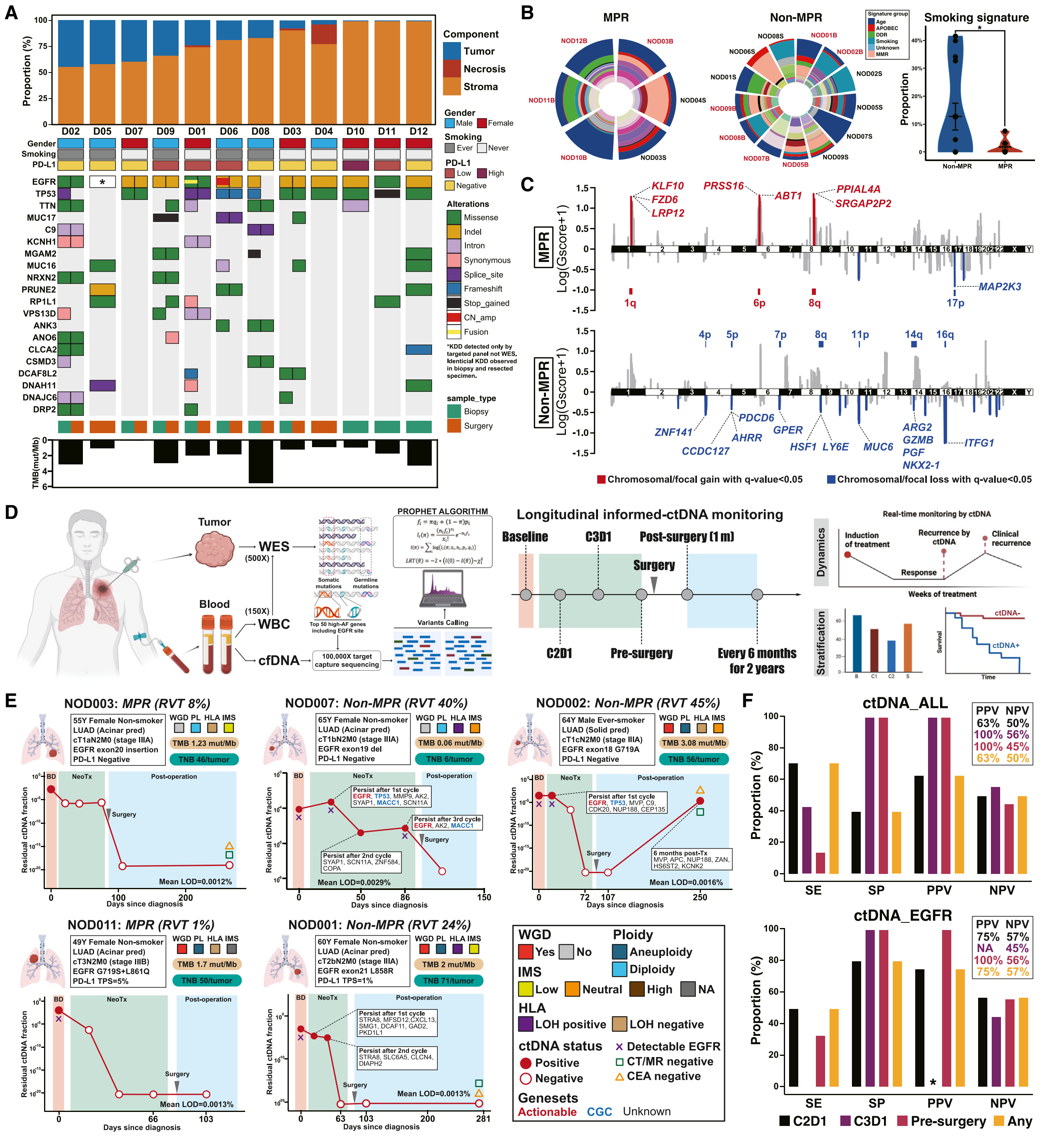

Figure 3. Genomic features of tumor specimens and longitudinal monitoring of informed MRD detection

(A) WES analysis of pre- and post-treatment tumor specimens along with specific pathological response assessment.

(B) Mutational signature analysis and comparison of smoking signature between MPR and nMPR patients. Dominant signature groups are specifically annotated. Proportions of smoking signature with different response groups were compared using Student's t test.

(C) Comparison of focal and chromosomal copy number variants (CNVs) between MPR and nMPR patients. Gscore was used to quantify the value, and bar plots indicate the relative proportion of significant focal CNVs within various chromosomes. Chromosomal/focal gain and chromosomal/focal loss with q < 0.05 are presented in red and blue, respectively.

(D) Schematic of tumor-informed MRD and exploratory design.

(legend continued on next page)

mononuclear cells (PBMCs) and plasma cytokines highlighted a significant increase in $CD3^{+}CD4^{+}$ T cells and a decrease in $CD19^{+}$ B cells in nMPR patients, a trend not observed in MPR patients (Figures S3C and S3D; Table S4). Additionally, MPR patients demonstrated a significant reduction in plasma interleukin-8 (IL-8) levels after neoadjuvant immunochemotherapy but no significant reduction for IL-6 (Figures S3E and S3F; Table S5).

For the neoadjuvant setting, all 18 patients experienced treatment-related adverse events (TRAEs), 7 (39%) patients had grade 3/4 TRAEs, and one of them had a severe adverse event due to hemoptysis during percutaneous lung biopsy for proving suspected disease progression. The most common adverse events were alopecia (83%), nausea (78%), paresthesia (61%), and granulocytopenia (39%). Only one patient had a confirmed immune-related averse event of hyperthyroidism (Tables S6 and S7). Although all 18 patients underwent surgery, 3 patients (17%) had non-R0 resection due to intraoperative occult pleural metastasis, while others had definite surgical resection, and the occult pleural metastasis was pathologically confirmed to be pre-existing instead of disease progression. All patients had a minimally invasive lobectomy (one had bilobectomy) and systemic lymph node dissection. Median surgical duration, estimated intraoperative blood loss, and length of stage were 130 ± 23.2 min, 80 mL (range 20–210 mL), and 4 days, respectively. A severe intraoperative complication was found in one patient, and any-grade peripheral/hilar adhesion as well as intraoperative fibrosis were identified in 7 and 5 patients, respectively (Table S8). Postoperatively, three patients had grade 2 complications, as per the Clavien-Dindo assessment, which required interventions like blood transfusion and intravenous nutritional support.

Genomic architecture and correlation with response

Genomic analysis of pre- and/or post-treatment specimens was completed in the first 12 enrolled patients. The most common genomic alterations accompanying EGFR mutation were *TP53* (83.3%) and *TTN* (41.7%) (Figure 3A). Pretreatment *TP53* missense mutations occurred more frequently in MPR than in nMPR patients (80% vs. 14%, Fisher's test, $p = 0.07$) (Figure S4A; Table S9). Others, including those that were reported to be correlated with inferior efficacy toward EGFR-TKIs, such as *RB1* and *RBM10* mutations, were more common in MPR patients, along with T cell immunity-related genes such as *CD6*, *IFNA4*, and *CD248* mutations, though no significant difference was observed (Figures S4B and S4C). For 9 patients who had adequate resected specimens for WES, only one patient (11%) was not detected to have EGFR mutation after neoadjuvant immunotherapy (Figure 3A; Table S10), which further led to the question whether upfront immunotherapy might impact the efficacy of subsequent TKIs. We therefore established patient-derived organoids (PDOs) for the most poorly responding patients (NOD02 and NOD05) who harbored uncommon EGFR driver mutations (Figure S5A). Multiple immunohistochemistry (IHC) staining and paired WES confirmed relatively identical tumor origins (Figures S5A and S5B). Susceptibility testing indicated superior anti-tumor activity of second-generation EGFR-TKIs in these two PDOs, which was in concordance with historically clinical data for uncommon EGFR mutations.

Patients without a history of smoking were more likely to achieve MPR (100% vs. 50%, Fisher's test, $p = 0.04$). The genomic signature also indicated a relatively lower smoking signature enriched in patients with MPR ($p = 0.05$) (Figure 3B). Although no significant difference was found between groups regarding tumor mutation burden (TMB), tumor neoantigen burden, whole-genome doubling (WGD), aneuploidy, human leukocyte antigen loss of heterozygosity (HLA-LOH), chromosomal instability (CIN), and immune status (immune dN/dS), patients who achieved MPR were more likely to have negative HLA-LOH and higher CIN ($p = 0.08$) (Figure S6). We further looked up chromosomal/focal copy number variants (CNV) and found significant CNV amplifications in MPR patients that were correlated with antigen presentation and tumor suppressors, while significant CNV deletions in nMPR patients correlated with T cell immunomodulation and cell proliferation (Figure 3C).

Longitudinal changes of tumor-informed MRD

77 blood and tissue samples derived from these 12 patients were prospectively collected and analyzed using a tumor-informed personalized circulating tumor DNA (ctDNA) panel based on WES (PROPHET, Burning Rock Biotech, Guangzhou, China). The application of a PROPHET algorithm utilizing maximum likelihood estimation was employed to ascertain the presence of ctDNA (Figures 3D and S7A). A 50-gene panel was designed for all patients except for one with 20-gene panel due to limited genes detected through WES of the tumor (Table S11). The average limit of detection was 0.0015%, ranging from 0.0009% to 0.0032% (Table S12). All 12 patients had detectable ctDNA, and 81% (9 of 11) patients had detectable corresponding EGFR mutations at baseline (Figure S7B). The number of patients with positive ctDNA decreased during neoadjuvant treatment, and only one patient had detectable ctDNA before surgery and failed to achieve MPR (Figures S7C and S7D). No correlation between mean tumor molecules and MPR status during different courses of treatment was found, except for baseline ($p = 0.003$) (Figure S7E). Dynamic changes of tumor-informed ctDNA of each patient were presented along with corresponding clinicopathological and genomic features. Although

(E) Representative dynamic changes of tumor-informed MRD before and after neoadjuvant immunochemotherapy along with individual genomic and immunological features. Specifically, the red dots indicate positive ctDNA regarding the PROPHET algorithm, while red circles indicate negative ctDNA with a fraction of no more than 10^{-6}.
(F) Performance of longitudinal MRD detection for pathological response. Sensitivity, specificity, NPV, and PPV were used to determine the performance of MRD. Different colors represent different time points of MRD detection. Asterisks indicate no EGFR mutations detected at this time point, which led a to PPV of 0/0.
TMB, tumor mutation burden; RVT, residual viable tumor; WES, whole-exome sequencing; WBC, whole blood cell; MRD, minimal residual disease; SE, sensitivity; SP, specificity; PPV, positive predictive value; NPV, negative predictive value; WGD, whole-genome doubling; IMS, immune status; HLA-LOH, human leukocyte antigen loss of heterozygosity; CGC, cancer gene census.

one of 5 patients had detectable ctDNA 6 months after surgery, enhanced thoracic CT, intracranial MRI, and carcinoembryonic antigen remained negative for these 5 patients (Figures 3E and S8). We further analyzed detection sensitivity and specificity as well as positive predictive value (PPV)/negative predictive value (NPV) for pathological response with regard to ctDNA clearance for all customized mutations or EGFR mutation only across different treatment time points. A numerically higher PPV compared to NPV was found across different treatment cycles (Figure 3F). Moreover, nonresponsive patients also had a substantial reduction of the ctDNA fraction compared to responsive patients, although a 671-fold higher reduction of the ctDNA fraction was observed in responsive patients (Table S13). These findings might indicate the potential relevance of considering the PPV over the NPV for ctDNA monitoring in the neoadjuvant setting to predict an inferior pathological response. Specifically, the detection of ctDNA before surgery may offer relatively high PPVs and NPVs, regardless of the overall ctDNA level or the presence of EGFR mutation.

The CCR8$^+$ regulatory T/CXCL13$^+$ exhausted T cell phenotype as potential response predictor

Identifying the potential beneficiaries for EGFR-mutant NSCLC remains a significant clinical unmet needs. We performed in-depth single-cell RNA-seq (scRNA-seq)/TCR sequencing (TCR-seq) of cells derived from 11 resected tumors as well as 34 tumors from a real-world cohort, which were all confirmed to be wild-type lung adenocarcinoma (LUAD) or adeno-squamous carcinoma and received neoadjuvant immunochemotherapy as a control (Table S14). Detailed baseline clinicopathological features of both cohorts are shown in Figure S9A. After rigorous quality control and removal of low-quality and doublet cells, we obtained 308,196 cells from 44 patients. We performed clustering and cell type annotation, identified major lineages of lymphoid (N = 211,076) and myeloid (N = 31,547) cells as well as corresponding subtypes (Figures S9B and S10). Specifically, no significant difference was observed among major cellular lineages regarding MPR status (Figure S9C).

We first compared the difference of lymphoid subsets between the NEOTIDE and the real-world cohort, which exhibited similar MPR rates (Figure 4A). T/natural killer (NK) cells of the whole cohorts were reclustered (Figure 4B), and no statistical difference was observed among various subsets for patients who achieved MPR with immunotherapy (Figure 4C). Additionally, clonal analysis revealed that both cohorts showed shared similar TCR expansion patterns with regard to CD8$^+$ FGFBP2$^+$ effector T (Teff), ZNF683$^+$ resident memory T (Trm), CXCL13$^+$ exhausted T (Tex), and FGFBP2$^+$ NK cells, suggesting that immunochemotherapy could also yield a comparable immune response in EGFR-mutant NSCLC (Figure 4D). Further analysis of different subset fractions in the NEOTIDE cohort regarding MPR status revealed a significantly higher fraction of CD4$^+$ CCR8$^+$ Treg cells (p = 0.03) in nMPR patients, while a numerically higher fraction of CXCL13$^+$ Tex (p = 0.3) and FGFBP2$^+$ NK (p = 0.13) cells was observed in MPR patients (Figure 4E). We further analyzed the clonal expansion of all T cell subtypes in NEOTIDE cohort and found that CXCL13$^+$ Tex cells and FGFBP2$^+$ NK T cells had the most expanded ratio across different subtypes (Figures 4F and 4G). No remarkable clonal overlap was found among different CD4 or CD8$^+$ T cell subsets except for Foxp3$^+$ Treg cells and CCR8$^+$ Treg cells (Figure 4H), which were reported to be of consistent lineage.[25] Moreover, patients who exhibited immune sensitivity had notably higher precursor CXCL13$^+$ Tex cell clonal expansion and lower CXCL13$^+$ Tex cell expansion as well as a presence of relatively high activated Treg (CCR8$^+$) cell clonal expansion for highly resistant patients (Figure 4I).

We further investigated immunological features of CCR8$^+$ Treg cells and found that, compared to tradition Foxp3$^+$ Treg cells, CCR8$^+$ Treg cells exhibited a stronger immune-suppressive and activated phenotype (Figure 5A) as well as numerically higher clonal expansion (Figures 5B and 5C). Moreover, CCR8$^+$ Treg cells possessed a more tumor-reactive and immune-suppressive phenotype compared to FOXP3$^+$ Treg cells, with relatively higher expression of exhaustion and cytokine receptor markers along with strong enrichment of IL-2-STAT5 signaling, which has been reported to be involved in CD8$^+$ T cell exhaustion[26] (Figure 5D; Table S15). Previous studies have indicated efficacy correlation of CCR8$^+$ Treg cells and CXCL13$^+$ Tex cells with immunotherapy in multiple cancer types;[27,28] we therefore matched the infiltration ratio or TCR clonal expansion of CCR8$^+$ Treg cells and CXCL13$^+$ Tex cells with stratified clinical efficacy. While patients with PR showed relatively higher clonal expansion of CXCL13$^+$ Tex cells, patients highly resistant to immunochemotherapy had a high CCR8$^+$ Treg cell ratio and clonal expansion along with low CXCL13$^+$ Tex cell clonal expansion (Figures 5E and 5F). We also performed bulk RNA-seq for paired pre-treatment samples when qualitatively available and found that patients with the CCR8$^+$ Treg^{hi}/CXCL13$^+$ Tex^{hi} relevant phenotype tended to have a more favorable pathological response (Figure 5G; Table S15), which was confirmed by paired multiple immunohistochemistry among different response patterns (Figures 5H and S11). We speculated that highly activated Treg cells pre treatment represented an immune phenotype with more favorable tumor antigen recognition along with high Tex cells, while high CCR8$^+$ Treg cells after immunotherapy represented an irreversible suppressive phenotype. We then applied public datasets of 3 large-scale NSCLC[29] and melanoma[30,31] immunotherapy datasets. We demonstrated that patients with a pre-treatment CCR8$^+$ Treg^{hi}/CXCL13$^+$ Tex^{hi} relevant phenotype correlated with a superior response rate and long-term benefits regardless of PD-L1 status (Figure S12), while a high/low CCR8$^+$ Treg and low CXCL13$^+$ Tex phenotype captured in on-treatment tumors correlated with inferior efficacy and survival (Figures 5I and S13A–S13C). We proposed that this phenotype could be applicable to EGFR-mutant NSCLC treated with immunotherapy for discerning potential beneficiaries, but a larger sample size is warranted (Figure S13D).

Hallmark of residual tumor and immune interaction

To elucidate the characteristics of residual tumors, we first investigated pathological microscopic features and conducted multiple immunohistochemistry of residual tumors (Figures 6A and 6B). We identified two major patterns, including external and internal infiltrates of lymphocytes within the tumor bed, and that displaying external infiltrate was more likely occur in nMPR patients (Figure 6C). We next evaluated the HLA-LOH status, which

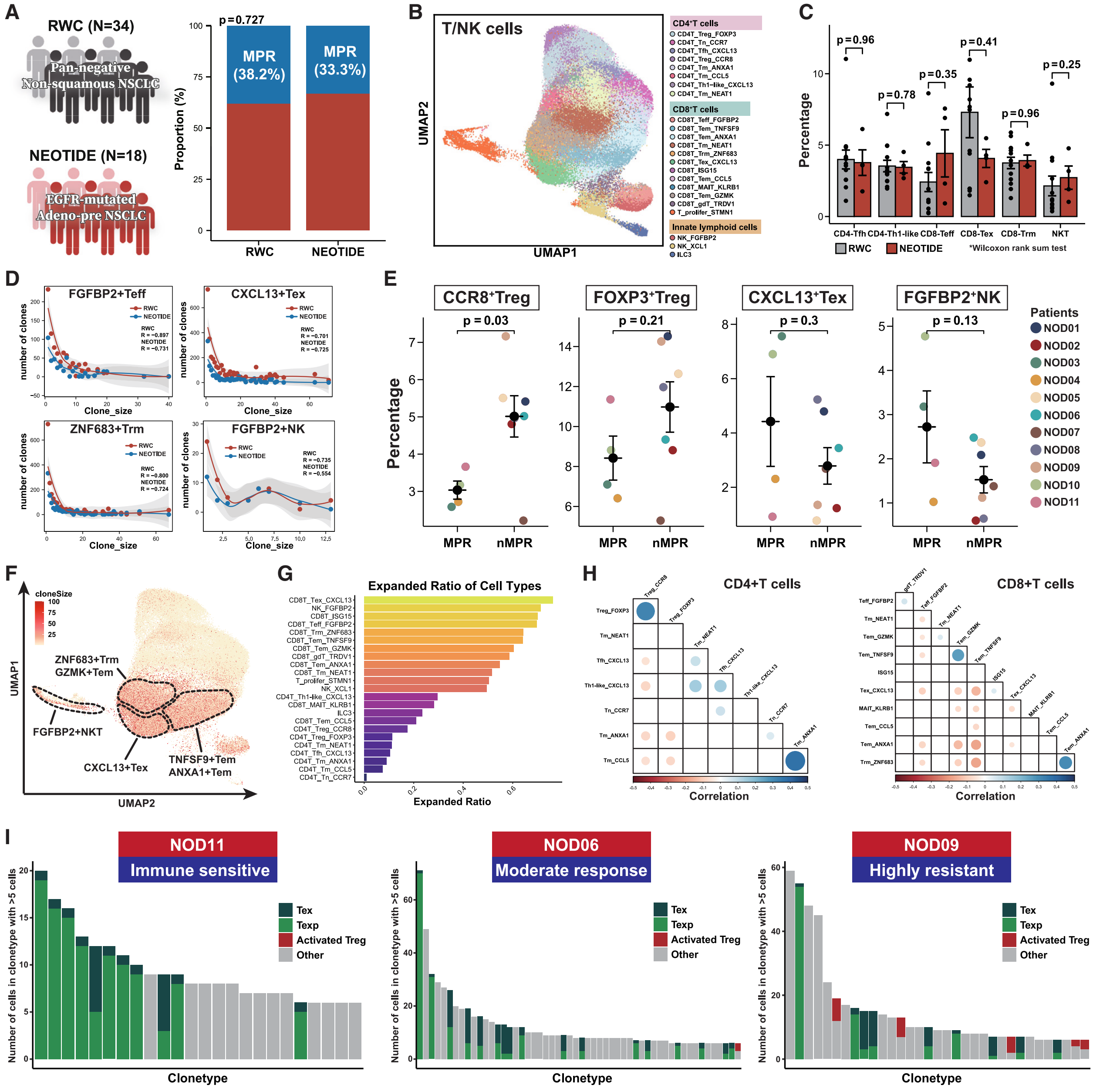

Figure 4. Differential TME phenotype of distinct pathological response in EGFR-mutant NSCLC

(A) Comparison of MPR (excluding metastatic lymph nodes) between the NEOTIDE and real-world cohorts.

(B) Reclustered T/NK cells and cell type annotation.

(C) Comparison of different T/NK cell subsets between the NEOTIDE and real-world cohorts. Wilcoxon rank-sum test was used to quantify the significance.

(D) Clonal analysis of different reactive cell subsets including FGFBP2^{+} Teff/NK cells, CXCL13^{+} Tex cells, and ZNF683^{+} Trm cells between the NEOTIDE and real-world cohorts.

(E) Percentages of reactive cell subsets between MPR and nMPR patients in the NEOTIDE cohort. Each colored dot represents different individuals, and Fisher's exact test was used to quantify the significance.

(F) Clonal expansion across all T cell subtypes through uniform manifold approximation and projection (UMAP). Clone size is noted, and cells of higher clonal expansion are marked in deep red. Dashed lines indicate T cell subsets of higher clonal expansion.

(G) Bar plot of expanded ratio across different T cell subtypes.

(H) Pearson correlation of clonal expansion among different T cell subtypes. Dots with dark blue indicate positive correlation, while dark red indicates the opposite.

(I) Representative individual clonal expansion regarding terminal Tex, precursor Tex, and activated Treg cells among different treatment responses.

has been proven to be one of the mechanisms that prevent tumor antigens from being recognized and lead to immune escape. WES-based HLA assessment revealed relatively higher pre-treatment HLA-LOH in nMPR patients, and IHC of HLA-A also verified that the mechanism of immune escape existed in EGFR-mutant NSCLC when treated with immunotherapy (Figure 6D). Intrigued by the possibility that the residual tumor might harbor potential therapeutic targets, we employed CopyKAT to filter the malignant component in epithelial cells (*N* = 1,669) (Figure 6E). Of note, TACSTD2 (Trop2) and CEACAM5/6 were expressed relatively highly in residual tumors, suggesting a potential strategy of corresponding ADC combination. Additionally, NT5E (CD73) was also highly significantly expressed in the hazard ratio (HR) group, though the expression level of CD73 was relatively low in general (Figure 6F). By analyzing differential genes in the HR group, we noticed that several genes, like SFTPD and CXCL14, that correlated with innate immune response were significantly enriched in the HR group (Figure 6G). Compared with the real-world control cohort, where no significant difference of innate immune cells was found, higher $CD16^{+}$ Mono and lower $FBP1^{+}$ Mφ were found in MPR patients (Figures 6H and S14). Further CellphoneDB analysis revealed a strong interaction of SFTPD-ADGRE5 and CXCL14-CXCR4 between malignant and myeloid cells, which might enhance macrophage infiltration and induce M2 polarization (Figure 6I). Similarly, IHC of CD163, a representative M2 marker, revealed similar findings of elevated infiltration in the HR group (Figure 6J). Together, these findings shed light on the underlying mechanism of incomplete effectiveness for residual tumors and suggest potential combination strategies with ADC for EGFR-mutant NSCLC.

DISCUSSION

Neoadjuvant immunotherapy plus chemotherapy has undoubtedly shifted the treatment paradigm for early-stage and locally advanced NSCLC without EGFR/ALK alterations.[32] While patients with oncogenic mutations have traditionally been perceived as not benefitting from immunotherapy, no prospective study to date has confirmed the inferior efficacy of neoadjuvant immunotherapy and chemotherapy in locally advanced, oncogene-mutant NSCLC. For localized EGFR-mutant NSCLC, despite the fact that osimertinib has been well established in the adjuvant setting, neoadjuvant EGFR-TKIs did not yield a satisfactory pathological response compared to immunotherapy, with an MPR ranging from only 10%–15%.[12,13] Moreover, whether chemotherapy is required in the postoperative setting has remained unclear,[33] which might leave room for immunotherapy plus chemotherapy to give the best shot in the neoadjuvant setting. In this ongoing phase 2 prospective study, we reported the short-term efficacy of neoadjuvant sintilimab (a PD-1 inhibitor) plus platinum-based chemotherapy in stage IIB-IIIB (excluding N3) EGFR-mutant NSCLC for stage I enrollment and showed that EGFR-mutant NSCLC could achieve comparable results through neoadjuvant immunotherapy plus chemotherapy. Despite no pCR being observed in the stage I cohort, neoadjuvant sintilimab plus chemotherapy could produce an MPR rate of 44.4% along with a tolerable safety profile and no disease progression, suggesting potential for conducting neoadjuvant immunotherapy and chemotherapy in EGFR-mutant NSCLC.

Most of the previous published trials involving neoadjuvant immunochemotherapy have excluded patients with known EGFR/ALK alterations. Although no MPR was found in EGFR/ALK-positive NSCLC treated with neoadjuvant atezolizumab alone regarding the LCMC3 study,[34] a phase II study enrolled 4 patients with EGFR mutations, of whom 2 achieved pCR after neoadjuvant atezolizumab plus chemotherapy.[20] Our center has also performed a retrospective multicenter study, showing that patients with EGFR mutations could yield a more superior pathological response compared to historical data of neoadjuvant EGFR-TKIs. Both studies applied carboplatin plus nab-PC as predominant chemotherapy regimens.[24] Therefore, in this ongoing phase 2 trial, we also used carboplatin plus nab-PC regimens regardless of histology. Overall, stage IIB-IIIB EGFR-mutant NSCLC treated with neoadjuvant sintilimab plus chemotherapy showed encouraging clinical efficacy and a favorable safety profile. Even for sensitive EGFR mutations (19del and 21L858R), such a treatment modality could provide an MPR rate of 30%. EGFR exon 20 insertion is a well-acknowledged EGFR subtype with poor prognosis and limited effective targeted drugs.[35] This study indicated a tremendous pathological response with an MPR rate of 75% through anti-PD-1 blockade plus chemotherapy despite the limited subgroup sample size. The underlying immune mechanism resulted in such deep pathological response with neoadjuvant PD-1 blockade plus chemotherapy in localized EGFR-mutant NSCLC remained unclear. We supposed that the chemotherapy regimen could be one of the key elements that increased the clinical efficacy, since all 3 randomized trials utilizing pemetrexed, platinum-based chemotherapy and PD-1 inhibitors failed to show remarkably improvement on survival,[36–38] and 2 studies using nab-PC in combination with PD-1/PD-L1 blockade exhibited encouraging pathological responses.[20,24] Also, another phase 3 prospective study comparing different chemotherapy regimens as an adjuvant setting for early-stage NSCLC exhibited significantly improved survival with vinorelbine, a similar anti-microtubule agent as (nab)paclitaxel, compared to traditional pemetrexed in EGFR-mutant LUAD,[39] implying that a (nab)paclitaxel-based regimen might be an ideal option for EGFR-mutant NSCLC.

By integrating genomic profiling for tumor specimens before and after neoadjuvant treatment, we discerned that neoadjuvant immunochemotherapy cased no significant alteration of the genomic landscape and demonstrated that the remaining tumor cells could still be sensitive to corresponding EGFR-TKIs *in vitro*. While we could not pinpoint mutations that could serve as markers for beneficiaries of immunochemotherapy in EGFR-mutant NSCLC, we did observe that certain pre-treatment mutations like *RBM10* and *RB1*, which have been previously linked to reduced EGFR-TKI efficacy in EGFR-mutant NSCLC,[40–42] occurred more frequently in patients achieving MPR after neoadjuvant immunochemotherapy. More importantly, we integrated the tumor-informed personalized MRD technique to longitudinally monitor the treatment efficacy of neoadjuvant treatment in patients who all bore specific EGFR mutations. A recent study reported that the ctDNA detection

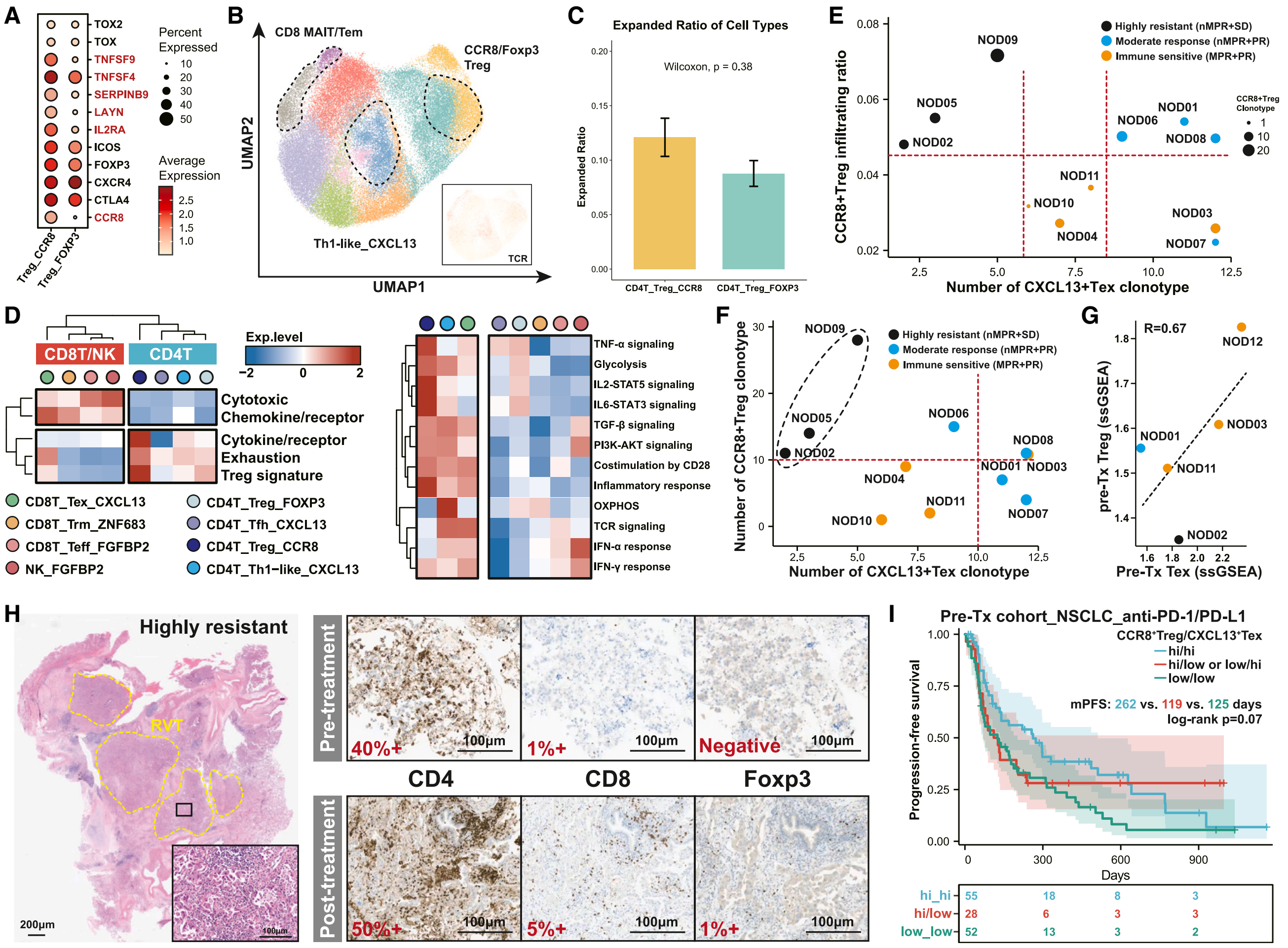

Figure 5. CCR8⁺ Treg cells and CXCL13⁺ Tex cells as potentially predictive biomarkers for immunotherapy

(A) Transcriptional difference between $CD4^+Foxp3^+$ Treg cells and $CCR8^+$ Treg cells in terms of suppressive and inflammatory genes.

(B) UMAP of $CD4^+$ T cell clusters and corresponding clonal expansion of $CXCL13^+$ Th1-like, $CCR8^+$ Treg cells and $FOPX3^+$ Treg cells.

(C) Difference of expanded ratio between CCR8+Treg and FOXP3+Treg. Wilcoxon was used to calculate the significance.

(D) Heatmap of enriched markers and functions among different CD4 and CD8 T subsets. Expression of specific markers or functions was normalized and underwent unsupervised clustering.

(E) Correlation of $CCR8^+$ Treg cell infiltration ratio and clonal expansion of $CXCL13^+$ Tex cells in patients exhibiting diverse response patterns.

(F) Scatterplot of TCR clonotype for $CCR8^+$ Treg cells and $CXCL13^+$ Tex cells among different response patterns, indicated by diverse colored dots. Red dotted lines represented the artificially defined cutoff to indicate high TCR expansion. The black dotted circles represented tumors with the worst response to immunochemotherapy.

(G) Degree of infiltrating $CCR8^+$ Treg cells and $CXCL13^+$ Tex cells, evaluated through single-sample gene set enrichment analysis (ssGSEA) in paired pre-treatment specimens. Correlation was analyzed by Pearson's correlation.

(H) Representative immunohistochemistry of CD4, CD8, and FOXP3 along with corresponding HE staining in a patient exhibiting highly resistant to immunochemotherapy.

(I) Comparison of survival after PD-1/PD-L1 inhibitors in external NSCLC and melanoma cohorts. Log rank p value was use to estimate the significance among groups.

rate for early-stage and locally advanced EGFR-mutant NSCLC is approximately 67% at baseline through droplet-digital polymerase chain reaction.[43] In our study, all patients had detectable ctDNA at baseline, of whom over 80% had detectable EGFR mutations, suggesting a more competitive platform with high sensitivity for the neoadjuvant setting. We also highlighted that the value of ctDNA monitoring in the neoadjuvant setting might rely on its PPV instead of NPV. Unlike the postoperative setting, where most patients would not undergo continuous systemic treatment, the treatment pressure in the neoadjuvant setting, especially chemotherapy, might significantly decrease the probability of ctDNA detection and lead to false-negative findings, which impact the value of NPV.[44] We also found that pre-surgery would be an ideal time point to detect MRD with relatively good performance of PPV in all customized mutations and EGFR mutations. This is of considerable importance for guiding the study design of dynamic monitoring for oncogene-mutant NSCLC.

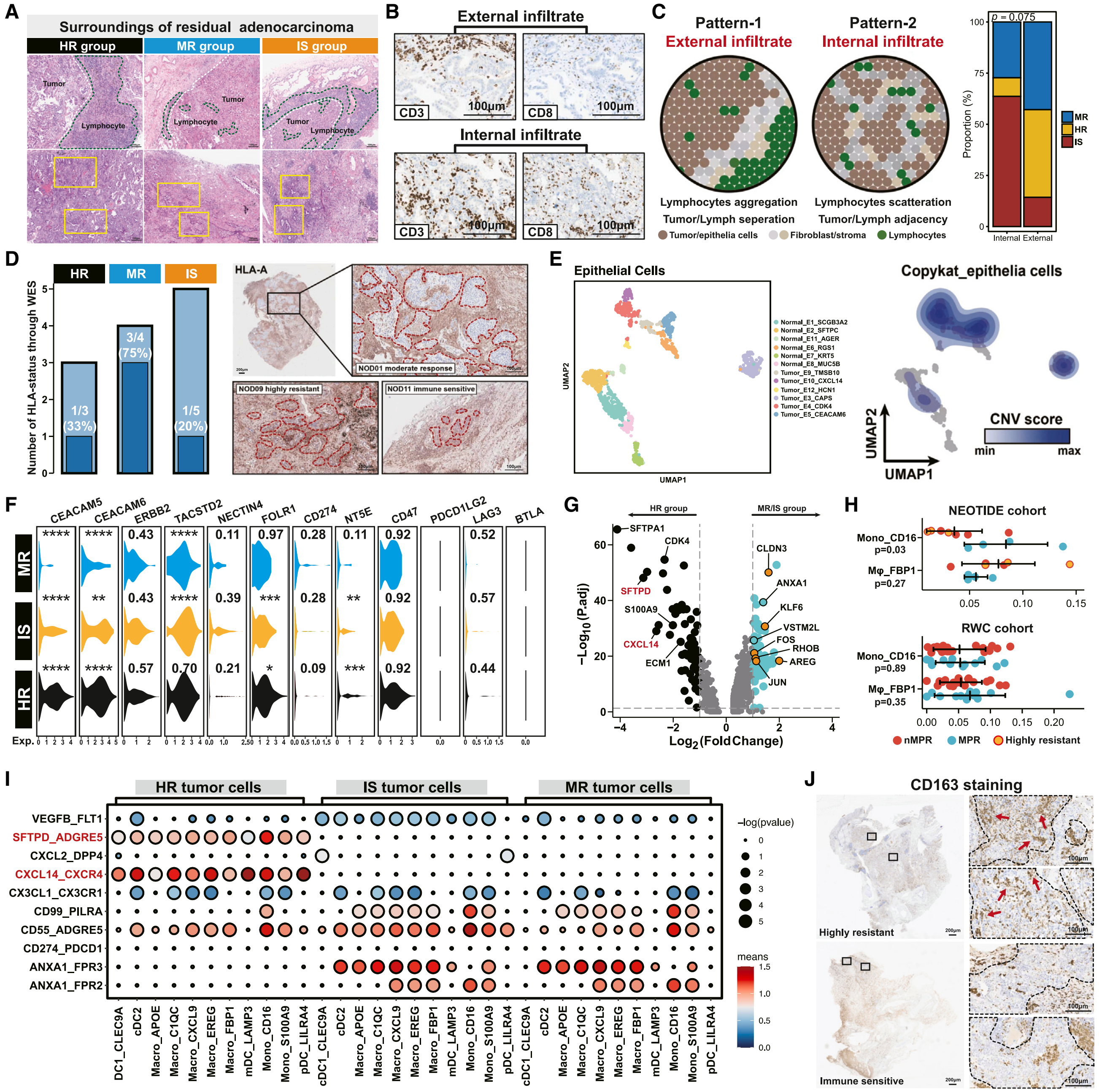

Figure 6. Extrinsic and intrinsic response mechanisms of residual tumors

(A) Microscopic pathological features through H&E staining among different response patterns. The green dotted circles indicate infiltrating immune cells, and the white dotted circles indicate tumor area.

(B) Immunohistochemistry of CD3 and CD8 staining between different response patterns.

(C) Schematic of diverse infiltration patterns within the tumor bed and surroundings. Proportions of different infiltrating patterns were calculated in terms of response patterns. Significance was analyzed using Fisher's exact test.

(D) WES-based HLA analysis and immunohistochemistry of HLA-A staining. A bar plot was used to compare proportions of HLA-LOH among different response patterns.

(E) Reclustering of epithelial cells and CopyKAT analysis.

(F) Expression levels of immune checkpoints and tumor-associated antigen for residual tumors among groups. p values of the top, intermediate, and low row represented MR vs. IS, IS vs. HR, and HR vs. MR, respectively. $^*p < 0.05$, $^{**}p < 0.01$, $^{***}p < 0.001$, $^{****}p < 0.0001$.

(G) Differential genes expressed in the HR and MR/IS groups. Cancer- or immune-relevant genes are specifically annotated.

(legend continued on next page)

A key result of the scRNA-seq/TCR-seq analyses in this cohort was that we defined a highly resistant subgroup of EGFR-mutant NSCLC in response to neoadjuvant immunochemotherapy, which was characterized as high infiltration and expansion of CCR8$^+$ Treg cells and low expansion of CXCL13$^+$ Tex cells. A previous study reported a non-inflamed tumor microenvironment (TME) with high CD4$^+$ Treg cell infiltration in pre-treated EGFR-mutant NSCLC.[45] We further deepened the concept and found that CCR8$^+$ Treg cells, unlike tradition FOXP3$^+$ Treg cells, displayed high expression of exhaustion and cytokine receptors and strongly correlated with inflammatory response, suggesting its highly activated and tumor-reactive phenotypes.[28] Besides, we demonstrated that EGFR-mutant NSCLC treated with neoadjuvant immunochemotherapy might undergo similar immune infiltration and TCR clonal expansion as wild-type LUAD, indicating the clinical feasibility of neoadjuvant immunochemotherapy in localized EGFR-mutant NSCLC. By analyzing available paired tumor samples, we put forward a first-defined classification of pre-treatment immune phenotype stratified through CCR8$^+$ Treg cells and CXCL13$^+$ Tex cells to identify those who would benefit more from immunotherapy, which has been verified by multiple large-scale immunotherapy cohorts. We supposed that infiltration of pre-treatment and post-treatment CCR8$^+$ Treg cells represented a diverse immune status and opposite predictive value for immunotherapy. Indeed, it should be interpreted with caution, since it has only been verified in EGFR-mutant NSCLC through limited sample size. Apart from the immune microenvironment, we also explore the hallmarks of residual EGFR-mutant tumor cells and interaction with immune cells after neoadjuvant immunochemotherapy. We identified two predominant lymphocyte patterns for surroundings of residual tumors, which was in line with some previous studies suggesting that surrounding cancer-associated fibrosis could prevent lymphocyte infiltration and induce immune-suppressive conditions.[46,47] Besides, we demonstrated that HLA-LOH was another major influential factor that impair the efficacy of neoadjuvant immunochemotherapy in EGFR-mutant NSCLC, which has also been illustrated in multiple cancer subtypes treated with immunotherapy.[48,49] Analysis of the residual malignant tumor cells revealed notably higher CD73 expression in the highly resistant subgroup and extensive TACSTD2 (TROP2) expression, which would also enlighten the study design of an ADC combination strategy as a perioperative setting for EGFR-mutant NSCLC.[50]

In summary, neoadjuvant sintilimab plus nab-PC demonstrates acceptable clinical feasibility and a tolerable safety profile in locally advanced EGFR-mutant NSCLC, including those with EGFR insertion and other uncommon subtypes. These data complement neoadjuvant immunochemotherapy as standard-of-care treatments in localized wild-type NSCLC and provide insights into potential combination strategies for future neoadjuvant treatment design. This ongoing study will further evaluate long-term survival outcome, testifying to the role of tumor-informed MRD detection during the perioperative period and verifying the value of pretreatment immune phenotype in selecting potential beneficiaries of immunotherapy in EGFR-mutant NSCLC.

Limitations of the study

The study is constrained by the small sample size, and no survival outcome has been reported. It is important to note that this represents the stage 1 assessment for a Simon's two-stage design study, and it has already met the endpoint for the stage I cohort, with no fewer than 3 patients achieving MPR. The study is still ongoing, and the survival outcome will be evaluated once the predefined condition is met, with over half of the total participants having undergone at least 1-year follow-up after 35 participants have been enrolled. We also recognize that the clinical efficacy of EGFR-TKIs in EGFR-mutant NSCLC after immunochemotherapy has only been demonstrated *in vitro*. Clinical data are needed to clarify whether upfront immunochemotherapy might influence the efficacy and safety profile of EGFR-TKIs after disease recurrence. Additionally, the findings of exploratory analysis for response and resistance mechanisms in the current study could be limited by the sample size and dimensional analysis. Furthermore, the stratification of immune phenotype based on pre-treatment CCR8$^+$ Treg cells and CXCL13$^+$ Tex cells in EGFR-mutant NSCLC requires a larger sample size to further illustrate its predictive value for immunotherapy. Last, it is essential to consider whether baseline genomic features and longitudinal detection of MRD could help in implementing more personalized treatment for early-stage and locally advanced EGFR-mutant NSCLC, which warrants a longer duration of surveillance.

STAR★METHODS

Detailed methods are provided in the online version of this paper and include the following:

- KEY RESOURCES TABLE
- RESOURCE AVAILABILITY
  - Lead contact
  - Materials availability
  - Data and code availability
- EXPERIMENTAL MODELS AND STUDY PARTICIPANT DETAILS
  - Human subjects and ethical approval
- METHOD DETAILS
  - Study design
  - Sample size justification
  - Pathological assessment
  - PD-L1 assessment through IHC
  - DNA extraction and quality control
  - Whole exome library preparation, sequencing and analysis platform
  - Whole exome data analysis
  - Whole transcriptome sequencing and analysis
  - Patient derived organoids (PDOs) establishment
  - Drug treatment and sensitivity tests
  - Circulating tumor DNA (ctDNA) analysis

(H) Proportion comparison of selected myeloid subsets between the NEOTIDE and real-world cohorts. Colored dots represent different treatment responses. Student's t test was used to measure the significance.

(I) CellphoneDB analysis of selected ligand-receptor interactions between tumor cells and myeloid subsets.

(J) Immunohistochemistry of CD163 staining within the tumor bed.

- Flow cytometry (FACS) and multiplex cytokines of peripheral samples
- Tissue dissociation and scRNA-seq
- Single-cell RNA sequencing data processing
- Clustering and identification of cell types
- Single-cell TCR data processing and analysis
- Gene module enrichment analysis
- Identification of malignant cells and differential expression analysis
- Cell-cell interaction analysis
- External Validation using bulk RNA data
- QUANTIFICATION AND STATISTICAL ANALYSIS
- ADDITIONAL RESOURCES

SUPPLEMENTAL INFORMATION

Supplemental information can be found online at https://doi.org/10.1016/j.xcrm.2024.101615.

ACKNOWLEDGMENTS

The authors thank the patients and their families, the study investigators and clinical staff, and the collaborating laboratories for biomarker testing. The CTONG2104 (NEOTIDE) study was partially funded by Innovent Biologics and Eli Lilly. This study was also supported by the National Science Foundation of China (grant 82241235), the National High-level Personnel of Special Support Program (Ten Thousand Talents Plan) (KA0120231004), the Guangdong Basic and Applied Basic Research Foundation (2019B1515130002), the High-level Hospital Construction Project (DFJH201801), and the Guangdong Provincial Key Laboratory of Lung Cancer Translational Medicine (2017B030314120). The graphic abstract was created with BioRender.

AUTHOR CONTRIBUTIONS

The CTONG2104 (NEOTIDE) study was conceived by C.Z. and W.-Z.Z., including development of the study protocol. W.-Z.Z., Y.-L.W., and Z.-M.Z. initiated and led the study. C.Z. and Y.-X.S. designed the experiments. C.Z., Y.-X.S., D.-C.Y., Z.-D.L., and W.-J.Z. performed the experiments and analyzed scRNA-seq data. C.Z. and L.-S.P. collected clinical samples. C.Z., B.-Y.J., H.S., and Y.-S.L. collected clinical data and verified radiological response. L.-X.Y. performed pathological response evaluation and immunohistochemistry staining. X.-Y.T. provided paired radiological images analysis. C.Z., B.-Y.J., L.-S.P., Z.-Y.C., J.Y., R.-Q.L., S.D., C.-R.X., Q.Z., X.-N.Y., and W.-Z.Z. assisted with patient enrollment and informed consent. S.-Q.L. and Z.Z. performed PDO establishment and subsequent experiments. C.Z., S.-A.C., D.P., and D.-H.W. performed analysis of MRD and DNA-based sequencing data. C.Z., Y.-X.S., and D.-C.Y. created all figures and drafted the manuscript. All authors assisted with data interpretation, provided important suggestions, approved the final draft, and agreed to be accountable for the data reported here.

DECLARATION OF INTERESTS

W.-Z.Z.. received speech honoraria from AstraZeneca, Roche, Eli Lilly, and Pfizer outside the submitted work. Z.-M.Z. is a founder of Analytical BioSciences. Y.-L.W. received research funding from Roche and speech honoraria from AstraZeneca, Roche, Eli Lilly, Pfizer, and Sanofi, and he was a research consultant for AstraZeneca. Q.Z. reports honoraria from AstraZeneca, Boehringer Ingelheim, BMS, Eli Lilly, MSD, Pfizer, Roche, and Sanofi outside the submitted work.

Received: November 2, 2023
Revised: January 31, 2024
Accepted: May 23, 2024
Published: June 18, 2024

REFERENCES

1. Lynch, T.J., Bell, D.W., Sordella, R., Gurubhagavatula, S., Okimoto, R.A., Brannigan, B.W., Harris, P.L., Haserlat, S.M., Supko, J.G., Haluska, F.G., et al. (2004). Activating mutations in the epidermal growth factor receptor underlying responsiveness of non-small-cell lung cancer to gefitinib. N. Engl. J. Med. *350*, 2129–2139. https://doi.org/10.1056/NEJMoa040938.
2. Shi, Y., Au, J.S.K., Thongprasert, S., Srinivasan, S., Tsai, C.M., Khoa, M.T., Heeroma, K., Itoh, Y., Cornelio, G., and Yang, P.C. (2014). A prospective, molecular epidemiology study of EGFR mutations in Asian patients with advanced non-small-cell lung cancer of adenocarcinoma histology (PIONEER). J. Thorac. Oncol. *9*, 154–162. https://doi.org/10.1097/jto.0000000000000033.
3. Mok, T.S., Wu, Y.L., Thongprasert, S., Yang, C.H., Chu, D.T., Saijo, N., Sunpaweravong, P., Han, B., Margono, B., Ichinose, Y., et al. (2009). Gefitinib or carboplatin-paclitaxel in pulmonary adenocarcinoma. N. Engl. J. Med. *361*, 947–957. https://doi.org/10.1056/NEJMoa0810699.
4. Wu, Y.L., Cheng, Y., Zhou, X., Lee, K.H., Nakagawa, K., Niho, S., Tsuji, F., Linke, R., Rosell, R., Corral, J., et al. (2017). Dacomitinib versus gefitinib as first-line treatment for patients with EGFR-mutation-positive non-small-cell lung cancer (ARCHER 1050): a randomised, open-label, phase 3 trial. Lancet Oncol. *18*, 1454–1466. https://doi.org/10.1016/s1470-2045(17)30608-3.
5. Soria, J.C., Ohe, Y., Vansteenkiste, J., Reungwetwattana, T., Chewaskulyong, B., Lee, K.H., Dechaphunkul, A., Imamura, F., Nogami, N., Kurata, T., et al. (2018). Osimertinib in Untreated EGFR-Mutated Advanced Non-Small-Cell Lung Cancer. N. Engl. J. Med. *378*, 113–125. https://doi.org/10.1056/NEJMoa1713137.
6. Passaro, A., Jänne, P.A., Mok, T., and Peters, S. (2021). Overcoming therapy resistance in EGFR-mutant lung cancer. Nat. Can. (Ott.) *2*, 377–391. https://doi.org/10.1038/s43018-021-00195-8.
7. Blaquier, J.B., Ortiz-Cuaran, S., Ricciuti, B., Mezquita, L., Cardona, A.F., and Recondo, G. (2023). Tackling osimertinib resistance in EGFR mutant non-small cell lung cancer. Clin. Cancer Res. *29*, 3579–3591. https://doi.org/10.1158/1078-0432.Ccr-22-1912.
8. Skoulidis, F., and Heymach, J.V. (2019). Co-occurring genomic alterations in non-small-cell lung cancer biology and therapy. Nat. Rev. Cancer *19*, 495–509. https://doi.org/10.1038/s41568-019-0179-8.
9. Zhong, W.Z., Wang, Q., Mao, W.M., Xu, S.T., Wu, L., Wei, Y.C., Liu, Y.Y., Chen, C., Cheng, Y., Yin, R., et al. (2021). Gefitinib Versus Vinorelbine Plus Cisplatin as Adjuvant Treatment for Stage II-IIIA (N1-N2) EGFR-Mutant NSCLC: Final Overall Survival Analysis of CTONG1104 Phase III Trial. J. Clin. Oncol. *39*, 713–722. https://doi.org/10.1200/jco.20.01820.
10. Tada, H., Mitsudomi, T., Misumi, T., Sugio, K., Tsuboi, M., Okamoto, I., Iwamoto, Y., Sakakura, N., Sugawara, S., Atagi, S., et al. (2022). Randomized Phase III Study of Gefitinib Versus Cisplatin Plus Vinorelbine for Patients With Resected Stage II-IIIA Non-Small-Cell Lung Cancer With EGFR Mutation (IMPACT). J. Clin. Oncol. *40*, 231–241. https://doi.org/10.1200/jco.21.01729.
11. Tsuboi, M., Herbst, R.S., John, T., Kato, T., Majem, M., Grohé, C., Wang, J., Goldman, J.W., Lu, S., Su, W.C., et al. (2023). Overall Survival with Osimertinib in Resected EGFR-Mutated NSCLC. N. Engl. J. Med. *389*, 137–147. https://doi.org/10.1056/NEJMoa2304594.
12. Lv, C., Fang, W., Wu, N., Jiao, W., Xu, S., Ma, H., Wang, J., Wang, R., Ji, C., Li, S., et al. (2023). Osimertinib as neoadjuvant therapy in patients with EGFR-mutant resectable stage II-IIIB lung adenocarcinoma (NEOS): A multicenter, single-arm, open-label phase 2b trial. Lung Cancer *178*, 151–156. https://doi.org/10.1016/j.lungcan.2023.02.011.
13. Urisman, A., Gubens, M.A., Mulvey, C., Allen, G.M., Rotow, J.K., Kerr, D.L., Chakrabarti, T., Bacaltos, B., Gee, M., Jones, K., et al. (2023). Phase II trial of neoadjuvant osimertinib for surgically resectable EGFR-mutated non-small cell lung cancer. 2023 ASCO Annual Meeting *41*, 8508. https://doi.org/10.1200/JCO.2023.41.16_suppl.8508.

14. Ribas, A., and Wolchok, J.D. (2018). Cancer immunotherapy using checkpoint blockade. Science *359*, 1350–1355. https://doi.org/10.1126/science.aar4060.
15. Wei, S.C., Duffy, C.R., and Allison, J.P. (2018). Fundamental Mechanisms of Immune Checkpoint Blockade Therapy. Cancer Discov. *8*, 1069–1086. https://doi.org/10.1158/2159-8290.Cd-18-0367.
16. Borghaei, H., Ciuleanu, T.E., Lee, J.S., Pluzanski, A., Caro, R.B., Gutierrez, M., Ohe, Y., Nishio, M., Goldman, J., Ready, N., et al. (2023). Long-term survival with first-line nivolumab plus ipilimumab in patients with advanced non-small-cell lung cancer: a pooled analysis. Ann. Oncol. *34*, 173–185. https://doi.org/10.1016/j.annonc.2022.11.006.
17. Reck, M., Rodríguez-Abreu, D., Robinson, A.G., Hui, R., Csőszi, T., Fülöp, A., Gottfried, M., Peled, N., Tafreshi, A., Cuffe, S., et al. (2021). Five-Year Outcomes With Pembrolizumab Versus Chemotherapy for Metastatic Non-Small-Cell Lung Cancer With PD-L1 Tumor Proportion Score ≥ 50. J. Clin. Oncol. *39*, 2339–2349. https://doi.org/10.1200/jco.21.00174.
18. Forde, P.M., Spicer, J., Lu, S., Provencio, M., Mitsudomi, T., Awad, M.M., Felip, E., Broderick, S.R., Brahmer, J.R., Swanson, S.J., et al. (2022). Neoadjuvant Nivolumab plus Chemotherapy in Resectable Lung Cancer. N. Engl. J. Med. *386*, 1973–1985. https://doi.org/10.1056/NEJMoa2202170.
19. Lee, J.M., Tsuboi, M., and Brunelli, A. (2022). Surgical Perspective on Neoadjuvant Immunotherapy in Non-Small Cell Lung Cancer. Ann. Thorac. Surg. *114*, 1505–1515. https://doi.org/10.1016/j.athoracsur.2021.06.069.
20. Shu, C.A., Gainor, J.F., Awad, M.M., Chiuzan, C., Grigg, C.M., Pabani, A., Garofano, R.F., Stoopler, M.B., Cheng, S.K., White, A., et al. (2020). Neoadjuvant atezolizumab and chemotherapy in patients with resectable non-small-cell lung cancer: an open-label, multicentre, single-arm, phase 2 trial. Lancet Oncol. *21*, 786–795. https://doi.org/10.1016/s1470-2045(20)30140-6.
21. Lisberg, A., Cummings, A., Goldman, J.W., Bornazyan, K., Reese, N., Wang, T., Coluzzi, P., Ledezma, B., Mendenhall, M., Hunt, J., et al. (2018). A Phase II Study of Pembrolizumab in EGFR-Mutant, PD-L1+, Tyrosine Kinase Inhibitor Naïve Patients With Advanced NSCLC. J. Thorac. Oncol. *13*, 1138–1145. https://doi.org/10.1016/j.jtho.2018.03.035.
22. Lee, C.K., Man, J., Lord, S., Links, M., Gebski, V., Mok, T., and Yang, J.C.H. (2017). Checkpoint Inhibitors in Metastatic EGFR-Mutated Non-Small Cell Lung Cancer-A Meta-Analysis. J. Thorac. Oncol. *12*, 403–407. https://doi.org/10.1016/j.jtho.2016.10.007.
23. Nogami, N., Barlesi, F., Socinski, M.A., Reck, M., Thomas, C.A., Cappuzzo, F., Mok, T.S.K., Finley, G., Aerts, J.G., Orlandi, F., et al. (2022). IMpower150 Final Exploratory Analyses for Atezolizumab Plus Bevacizumab and Chemotherapy in Key NSCLC Patient Subgroups With EGFR Mutations or Metastases in the Liver or Brain. J. Thorac. Oncol. *17*, 309–323. https://doi.org/10.1016/j.jtho.2021.09.014.
24. Zhang, C., Chen, H.F., Yan, S., Wu, L., Yan, L.X., Yan, X.L., Yue, D.S., Xu, C.W., Zheng, M., Li, J.S., et al. (2022). Induction immune-checkpoint inhibitors for resectable oncogene-mutant NSCLC: A multicenter pooled analysis. npj Precis. Oncol. *6*, 66. https://doi.org/10.1038/s41698-022-00301-8.
25. Zheng, L., Qin, S., Si, W., Wang, A., Xing, B., Gao, R., Ren, X., Wang, L., Wu, X., Zhang, J., et al. (2021). Pan-cancer single-cell landscape of tumor-infiltrating T cells. Science (New York, N.Y.) *374*, abe6474. https://doi.org/10.1126/science.abe6474.
26. Hashimoto, M., Araki, K., Cardenas, M.A., Li, P., Jadhav, R.R., Kissick, H.T., Hudson, W.H., McGuire, D.J., Obeng, R.C., Wieland, A., et al. (2022). PD-1 combination therapy with IL-2 modifies CD8(+) T cell exhaustion program. Nature *610*, 173–181. https://doi.org/10.1038/s41586-022-05257-0.
27. Liu, B., Zhang, Y., Wang, D., Hu, X., and Zhang, Z. (2022). Single-cell meta-analyses reveal responses of tumor-reactive CXCL13(+) T cells to immune-checkpoint blockade. Nat. Can. (Ott.) *3*, 1123–1136. https://doi.org/10.1038/s43018-022-00433-7.
28. Barsheshet, Y., Wildbaum, G., Levy, E., Vitenshtein, A., Akinseye, C., Griggs, J., Lira, S.A., and Karin, N. (2017). CCR8(+)FOXp3(+) T(reg) cells as master drivers of immune regulation. Proc. Natl. Acad. Sci. USA *114*, 6086–6091. https://doi.org/10.1073/pnas.1621280114.
29. Ravi, A., Hellmann, M.D., Arniella, M.B., Holton, M., Freeman, S.S., Naranbhai, V., Stewart, C., Leshchiner, I., Kim, J., Akiyama, Y., et al. (2023). Genomic and transcriptomic analysis of checkpoint blockade response in advanced non-small cell lung cancer. Nat. Genet. *55*, 807–819. https://doi.org/10.1038/s41588-023-01355-5.
30. Liu, D., Schilling, B., Liu, D., Sucker, A., Livingstone, E., Jerby-Arnon, L., Zimmer, L., Gutzmer, R., Satzger, I., Loquai, C., et al. (2019). Integrative molecular and clinical modeling of clinical outcomes to PD1 blockade in patients with metastatic melanoma. Nat. Med. *25*, 1916–1927. https://doi.org/10.1038/s41591-019-0654-5.
31. Riaz, N., Havel, J.J., Makarov, V., Desrichard, A., Urba, W.J., Sims, J.S., Hodi, F.S., Martín-Algarra, S., Mandal, R., Sharfman, W.H., et al. (2017). Tumor and Microenvironment Evolution during Immunotherapy with Nivolumab. Cell *171*, 934–949.e16. https://doi.org/10.1016/j.cell.2017.09.028.
32. Passaro, A., Attili, I., and de Marinis, F. (2022). Neoadjuvant Chemotherapy Plus Immunotherapy in Early-Stage Resectable Non-Small-Cell Lung Cancer. J. Clin. Oncol. *40*, 2871–2877. https://doi.org/10.1200/jco.22.00873.
33. Wu, Y.L., John, T., Grohe, C., Majem, M., Goldman, J.W., Kim, S.W., Kato, T., Laktionov, K., Vu, H.V., Wang, Z., et al. (2022). Postoperative Chemotherapy Use and Outcomes From ADAURA: Osimertinib as Adjuvant Therapy for Resected EGFR-Mutated NSCLC. J. Thorac. Oncol. *17*, 423–433. https://doi.org/10.1016/j.jtho.2021.10.014.
34. Chaft, J.E., Oezkan, F., Kris, M.G., Bunn, P.A., Wistuba, I.I., Kwiatkowski, D.J., Owen, D.H., Tang, Y., Johnson, B.E., Lee, J.M., et al. (2022). Neoadjuvant atezolizumab for resectable non-small cell lung cancer: an open-label, single-arm phase II trial. Nat. Med. *28*, 2155–2161. https://doi.org/10.1038/s41591-022-01962-5.
35. Friedlaender, A., Subbiah, V., Russo, A., Banna, G.L., Malapelle, U., Rolfo, C., and Addeo, A. (2022). EGFR and HER2 exon 20 insertions in solid tumours: from biology to treatment. Nat. Rev. Clin. Oncol. *19*, 51–69. https://doi.org/10.1038/s41571-021-00558-1.
36. Lu, S., Wu, L., Jian, H., Chen, Y., Wang, Q., Fang, J., Wang, Z., Hu, Y., Sun, M., Han, L., et al. (2022). Sintilimab plus bevacizumab biosimilar IBI305 and chemotherapy for patients with EGFR-mutated non-squamous non-small-cell lung cancer who progressed on EGFR tyrosine-kinase inhibitor therapy (ORIENT-31): first interim results from a randomised, double-blind, multicentre, phase 3 trial. Lancet Oncol. *23*, 1167–1179. https://doi.org/10.1016/s1470-2045(22)00382-5.
37. Mok, T.S.K., Nakagawa, K., Park, K., Ohe, Y., Girard, N., Kim, H.R., Wu, Y.L., Gainor, J., Lee, S.H., Chiu, C.H., et al. (2022). LBA8 Nivolumab (NIVO) + chemotherapy (chemo) vs chemo in patients (pts) with EGFR-mutated metastatic non-small cell lung cancer (mNSCLC) with disease progression after EGFR tyrosine kinase inhibitors (TKIs) in CheckMate 722. Ann. Oncol. *33*, S1561–S1562. https://doi.org/10.1016/j.annonc.2022.10.350.
38. Yang, J.C.H., Lee, D.H., Lee, J.S., Fan, Y., de Marinis, F., Okamoto, I., Inoue, T., Rodriguez Cid, J.R., Zhang, L., Yang, C.T., et al. (2023). Pemetrexed and platinum with or without pembrolizumab for tyrosine kinase inhibitor (TKI)-resistant, EGFR-mutant, metastatic nonsquamous NSCLC: Phase 3 KEYNOTE-789 study. J. Clin. Oncol. *41*, LBA9000. https://doi.org/10.1200/JCO.2023.41.17_suppl.LBA9000.
39. Kenmotsu, H., Yamamoto, N., Yamanaka, T., Yoshiya, K., Takahashi, T., Ueno, T., Goto, K., Daga, H., Ikeda, N., Sugio, K., et al. (2020). Randomized Phase III Study of Pemetrexed Plus Cisplatin Versus Vinorelbine Plus Cisplatin for Completely Resected Stage II to IIIA Nonsquamous Non-Small-Cell Lung Cancer. J. Clin. Oncol. *38*, 2187–2196. https://doi.org/10.1200/jco.19.02674.
40. Ferrara, M.G., Belluomini, L., Smimmo, A., Sposito, M., Avancini, A., Giannarelli, D., Milella, M., Pilotto, S., and Bria, E. (2023). Meta-analysis of the

prognostic impact of TP53 co-mutations in EGFR-mutant advanced non-small-cell lung cancer treated with tyrosine kinase inhibitors. Crit. Rev. Oncol. Hematol. *184*, 103929. https://doi.org/10.1016/j.critrevonc.2023.103929.

41. Nanjo, S., Wu, W., Karachaliou, N., Blakely, C.M., Suzuki, J., Chou, Y.T., Ali, S.M., Kerr, D.L., Olivas, V.R., Shue, J., et al. (2022). Deficiency of the splicing factor RBM10 limits EGFR inhibitor response in EGFR-mutant lung cancer. J. Clin. Invest. *132*, e145099. https://doi.org/10.1172/jci145099.
42. Foggetti, G., Li, C., Cai, H., Hellyer, J.A., Lin, W.Y., Ayeni, D., Hastings, K., Choi, J., Wurtz, A., Andrejka, L., et al. (2021). Genetic Determinants of EGFR-Driven Lung Cancer Growth and Therapeutic Response In Vivo. Cancer Discov. *11*, 1736–1753. https://doi.org/10.1158/2159-8290.Cd-20-1385.
43. Jung, H.A., Ku, B.M., Kim, Y.J., Park, S., Sun, J.M., Lee, S.H., Ahn, J.S., Cho, J.H., Kim, H.K., Choi, Y.S., et al. (2023). Longitudinal Monitoring of Circulating Tumor DNA From Plasma in Patients With Curative Resected Stages I to IIIA EGFR-Mutant Non-Small Cell Lung Cancer. J. Thorac. Oncol. *18*, 1199–1208. https://doi.org/10.1016/j.jtho.2023.05.027.
44. Pellini, B., and Chaudhuri, A.A. (2022). Circulating Tumor DNA Minimal Residual Disease Detection of Non-Small-Cell Lung Cancer Treated With Curative Intent. J. Clin. Oncol. *40*, 567–575. https://doi.org/10.1200/jco.21.01929.
45. Sugiyama, E., Togashi, Y., Takeuchi, Y., Shinya, S., Tada, Y., Kataoka, K., Tane, K., Sato, E., Ishii, G., Goto, K., et al. (2020). Blockade of EGFR improves responsiveness to PD-1 blockade in EGFR-mutated non-small cell lung cancer. Sci. Immunol. *5*, eaav3937. https://doi.org/10.1126/sciimmunol.aav3937.
46. Hu, H., Piotrowska, Z., Hare, P.J., Chen, H., Mulvey, H.E., Mayfield, A., Noeen, S., Kattermann, K., Greenberg, M., Williams, A., et al. (2021). Three subtypes of lung cancer fibroblasts define distinct therapeutic paradigms. Cancer Cell *39*, 1531–1547.e10. https://doi.org/10.1016/j.ccell.2021.09.003.
47. Grout, J.A., Sirven, P., Leader, A.M., Maskey, S., Hector, E., Puisieux, I., Steffan, F., Cheng, E., Tung, N., Maurin, M., et al. (2022). Spatial Positioning and Matrix Programs of Cancer-Associated Fibroblasts Promote T-cell Exclusion in Human Lung Tumors. Cancer Discov. *12*, 2606–2625. https://doi.org/10.1158/2159-8290.Cd-21-1714.
48. Sade-Feldman, M., Jiao, Y.J., Chen, J.H., Rooney, M.S., Barzily-Rokni, M., Eliane, J.P., Bjorgaard, S.L., Hammond, M.R., Vitzthum, H., Blackmon, S.M., et al. (2017). Resistance to checkpoint blockade therapy through inactivation of antigen presentation. Nat. Commun. *8*, 1136. https://doi.org/10.1038/s41467-017-01062-w.
49. Gettinger, S., Choi, J., Hastings, K., Truini, A., Datar, I., Sowell, R., Wurtz, A., Dong, W., Cai, G., Melnick, M.A., et al. (2017). Impaired HLA Class I Antigen Processing and Presentation as a Mechanism of Acquired Resistance to Immune Checkpoint Inhibitors in Lung Cancer. Cancer Discov. *7*, 1420–1435. https://doi.org/10.1158/2159-8290.Cd-17-0593.
50. Tu, E., McGlinchey, K., Wang, J., Martin, P., Ching, S.L., Floc'h, N., Kurasawa, J., Starrett, J.H., Lazdun, Y., Wetzel, L., et al. (2022). Anti-PD-L1 and anti-CD73 combination therapy promotes T cell response to EGFR-mutated NSCLC. JCI insight *7*, e142843. https://doi.org/10.1172/jci.insight.142843.
51. Chen, S., Zhou, Y., Chen, Y., and Gu, J. (2018). fastp: an ultra-fast all-in-one FASTQ preprocessor. Bioinformatics *34*, i884–i890. https://doi.org/10.1093/bioinformatics/bty560.
52. Wang, K., Li, M., and Hakonarson, H. (2010). ANNOVAR: functional annotation of genetic variants from high-throughput sequencing data. Nucleic Acids Res. *38*, e164. https://doi.org/10.1093/nar/gkq603.
53. Cingolani, P., Platts, A., Wang, L.L., Coon, M., Nguyen, T., Wang, L., Land, S.J., Lu, X., and Ruden, D.M. (2012). A program for annotating and predicting the effects of single nucleotide polymorphisms, SnpEff: SNPs in the genome of Drosophila melanogaster strain w1118; iso-2; iso-3. Fly *6*, 80–92. https://doi.org/10.4161/fly.19695.
54. Zapata, L., Pich, O., Serrano, L., Kondrashov, F.A., Ossowski, S., and Schaefer, M.H. (2018). Negative selection in tumor genome evolution acts on essential cellular functions and the immunopeptidome. Genome Biol. *19*, 67. https://doi.org/10.1186/s13059-018-1434-0.
55. Mermel, C.H., Schumacher, S.E., Hill, B., Meyerson, M.L., Beroukhim, R., and Getz, G. (2011). GISTIC2.0 facilitates sensitive and confident localization of the targets of focal somatic copy-number alteration in human cancers. Genome Biol. *12*, R41. https://doi.org/10.1186/gb-2011-12-4-r41.
56. Bielski, C.M., Zehir, A., Penson, A.V., Donoghue, M.T.A., Chatila, W., Armenia, J., Chang, M.T., Schram, A.M., Jonsson, P., Bandlamudi, C., et al. (2018). Genome doubling shapes the evolution and prognosis of advanced cancers. Nat. Genet. *50*, 1189–1195. https://doi.org/10.1038/s41588-018-0165-1.
57. Burrell, R.A., McClelland, S.E., Endesfelder, D., Groth, P., Weller, M.C., Shaikh, N., Domingo, E., Kanu, N., Dewhurst, S.M., Gronroos, E., et al. (2013). Replication stress links structural and numerical cancer chromosomal instability. Nature *494*, 492–496. https://doi.org/10.1038/nature11935.
58. Shukla, A., Nguyen, T.H.M., Moka, S.B., Ellis, J.J., Grady, J.P., Oey, H., Cristino, A.S., Khanna, K.K., Kroese, D.P., Krause, L., et al. (2020). Chromosome arm aneuploidies shape tumour evolution and drug response. Nat. Commun. *11*, 449. https://doi.org/10.1038/s41467-020-14286-0.
59. Szolek, A., Schubert, B., Mohr, C., Sturm, M., Feldhahn, M., and Kohlbacher, O. (2014). OptiType: precision HLA typing from next-generation sequencing data. Bioinformatics *30*, 3310–3316. https://doi.org/10.1093/bioinformatics/btu548.
60. Jurtz, V., Paul, S., Andreatta, M., Marcatili, P., Peters, B., and Nielsen, M. (2017). NetMHCpan-4.0: Improved Peptide-MHC Class I Interaction Predictions Integrating Eluted Ligand and Peptide Binding Affinity Data. J. Immunol. *199*, 3360–3368. https://doi.org/10.4049/jimmunol.1700893.
61. Chen, K., Yang, F., Shen, H., Wang, C., Li, X., Chervova, O., Wu, S., Qiu, F., Peng, D., Zhu, X., et al. (2023). Individualized tumor-informed circulating tumor DNA analysis for postoperative monitoring of non-small cell lung cancer. Cancer Cell *41*, 1749–1762.e6. https://doi.org/10.1016/j.ccell.2023.08.010.
62. Hua, H., He, W., Chen, N., He, Y., Wu, G., Ye, F., Zhou, X., Li, Y., Ding, Y., Zhong, W., et al. (2022). Genomic and transcriptomic analysis of MSI-H colorectal cancer patients with targetable alterations identifies clinical implications for immunotherapy. Front. Immunol. *13*, 974793. https://doi.org/10.3389/fimmu.2022.974793.
63. Korsunsky, I., Millard, N., Fan, J., Slowikowski, K., Zhang, F., Wei, K., Baglaenko, Y., Brenner, M., Loh, P.R., and Raychaudhuri, S. (2019). Fast, sensitive and accurate integration of single-cell data with Harmony. Nat. Methods *16*, 1289–1296. https://doi.org/10.1038/s41592-019-0619-0.
64. Gao, R., Bai, S., Henderson, Y.C., Lin, Y., Schalck, A., Yan, Y., Kumar, T., Hu, M., Sei, E., Davis, A., et al. (2021). Delineating copy number and clonal substructure in human tumors from single-cell transcriptomes. Nat. Biotechnol. *39*, 599–608. https://doi.org/10.1038/s41587-020-00795-2.
65. Garcia-Alonso, L., Lorenzi, V., Mazzeo, C.I., Alves-Lopes, J.P., Roberts, K., Sancho-Serra, C., Engelbert, J., Marečková, M., Gruhn, W.H., Botting, R.A., et al. (2022). Single-cell roadmap of human gonadal development. Nature *607*, 540–547. https://doi.org/10.1038/s41586-022-04918-4.
66. Hänzelmann, S., Castelo, R., and Guinney, J. (2013). GSVA: gene set variation analysis for microarray and RNA-seq data. BMC Bioinf. *14*, 7. https://doi.org/10.1186/1471-2105-14-7.
67. Lander, E.S., Linton, L.M., Birren, B., Nusbaum, C., Zody, M.C., Baldwin, J., Devon, K., Dewar, K., Doyle, M., FitzHugh, W., et al. (2001). Initial sequencing and analysis of the human genome. Nature *409*, 860–921. https://doi.org/10.1038/35057062.

STAR★METHODS

KEY RESOURCES TABLE

REAGENT or RESOURCE	SOURCE	IDENTIFIER
Antibodies		
Rabbit anti CK-7	ProteinTech	Cat # 17513-1-AP; RRID: AB_2134468
Rabbit anti TTF1	HuaBio	Cat # ER1902-68; RRID: AB_3069452
Rabbit anti EGFR EP38Y	Abcam	Cat # 1902-1; RRID: AB_764519
Rabbit anti HLA-A EP1395Y	Abcam	Cat # ab52922; RRID: AB_881225
Rabbit anti CD3 2GV6	Roche Diagnostics	Cat # 790–4341; RRID: AB_2335978
Rabbit anti CD4 SP35	Roche Diagnostics	Cat # 790–4423; RRID: AB_2335982
Rabbit anti CD8 SP16	GeneTex	Cat # GTX16696; RRID: AB_422355
Mouse anti CD20 L26	Roche Diagnostics	Cat # 760–2531; RRID: AB_2335956
Mouse anti CD163 10D6	Thermo Fisher Scientific	Cat # MA5-11458; RRID: AB_10982556
Mouse anti Foxp3 mAbcam22510	Abcam	Cat # ab22510; RRID: AB_447114
Mouse anti CD235a S21003A	Biolegend	Cat # 116702; RRID: AB_2924454
Mouse anti PD-L1 Dako22C3	PharmDx	Cat #M3653; RRID: AB_2861298
CD4 13B8.2	Beckman	Cat # IM2468; RRID: AB_130781
CD127 HIL-7R-M21	BD Bioscience	Cat # 557938; RRID: AB_1645486
CD25 M-A251	BD Bioscience	Cat # 561399; RRID: AB_10643029
CD69 FN50	Thermo Fisher Scientific	Cat # 12-0699-42; RRID: AB_10733526
CD8 RPA-T8	BD Bioscience	Cat # 347314; RRID: AB_400280
CD19 HIB19	BD Bioscience	Cat # 555415; RRID: AB_398597
CD5 L17F12	BD Bioscience	Cat # 341109; RRID: AB_2868765
CD28 CD28.2	BD Bioscience	Cat # 556620; RRID: AB_396492
CD3 UCHT1	Beckman	Cat # 6607100; RRID: AB_1575958
Biological samples		
Pre-treatment biopsy samples	This study	N/A
Post-treatment resected samples	This study	N/A
Patient blood samples	This study	N/A
Chemicals, peptides, and recombinant proteins		
Dimethyl sulfoxide	Aladdin	Cat #D103281-500mL CAS:67-78-5
10×Phosphate Buffered Saline	Biosharp	Cat # BL316A
TrypLE^{TM} Express	Thermo Fisher Scientific	Cat # 12604021
Trypan blue	DING GUO PROSPEROUS	Cat # AR-0761
Matrigel matrix	Corning	Cat # 356231
CellCounting-Lite 3D Luminescent Cell Viability Assay	Vazyme	Cat # DD1102-1
7-AAD Viability Staining Solution	Biolegend	Cat # 420404
Advanced DMEM/F12	Thermo Fisher Scientific	Cat # 12634028
B-27 supplement	Thermo Fisher Scientific	Cat # 17504044
Penicillin/streptomycin	Thermo Fisher Scientific	Cat # 15140122
Antibiotic-Antimycotic	Thermo Fisher Scientific	Cat # 15240062
HEPES	Thermo Fisher Scientific	Cat # 15630080
GlutaMax	Thermo Fisher Scientific	Cat # 35050061
N-acetyl-L-cysteine	Sigma Aldrich	Cat # A9165
Primocin	InvivoGene	Cat # ant-pm-1

(*Continued on next page*)

CellPress
OPEN ACCESS

Continued

REAGENT or RESOURCE	SOURCE	IDENTIFIER
Noggin	Bio-techne	Cat # 1967-NG
R-spondin 1	Bio-techne	Cat # 4645-RS
A 83-01	Cayman	Cat # 9001799
Y-27632	Selleck	Cat #S1049
FGF-7	OrganRegen	Cat # 923-FG7-1000
FGF-10	Novoprotein	Cat # CR11
SB202190	Selleck	Cat #S1077
Nicotinamide	Sigma Aldrich	Cat #N0636
Afatinib	Topscience	Cat #T21312<br>CAS:850140-72-6
Almonertinib	Topscience	Cat #T5462<br>CAS:1899921-05-1
Dacomitinib	Selleck	Cat #S2727<br>CAS:1110813-31-4
Erlotinib	Topscience	Cat #T0373<br>CAS:183321-74-6
Osimertinib	Topscience	Cat #T2490<br>CAS:1421373-65-0
Pemetrexed	Selleck	Cat #S5971<br>CAS:137281-23-3
**Critical commercial assays**		
QIAamp DNA FFPE tissue kit	Qiagen	Cat # 56404
MagPure FFPE DNA kit	Magen	Cat# D6323-02B
MagPure Universal DNA kit	Magen	Cat# MD5105-02
Magpure FFPE DNA/RNA kit	Magen	Cat# D6323-01R
QIAamp Circulating Nucleic Acid kit	Qiagen	Cat # 55114
QIAsymphony DSP Circulating DNA kit	Qiagen	Cat # 937556
Twist Human Core Exome kit	Twist Bioscience	Cat# 102027
Twist Fast Hybridization and Wash kit	Twist Bioscience	Cat# 104181
Tumor Dissociation Kit	Miltenyi	Cat # 130-095-929
Human Th1/Th2/Th17 14-plex (AimPlex)	QuantoBio	Cat #C191114
**Deposited data**		
Human reference genome full hg19/GRCh37	Genome Reference Consortium	N/A
Single cell RNA/TCR sequencing data	This paper	GEO: GSE241934
Whole-exome sequencing data	This paper	Supplementary Tables 9 and 10
Informed ctDNA data	This paper	Supplementary Tables 11 and 12
Bulk RNA sequencing data	This paper	GSE: HRA007419
RNA-Seq data Lung Cancer Checkpoint Blockade Response Project	Ravi et al.[29]	Phs002822.v1.p1
RNA-Seq data Melanoma Genome Sequencing Project	Liu et al.[30]	Phs000452.v3.p1
RNA-Seq data Immunotherapy with Nivolumab	Riaz et al.[31]	GEO: GSE91061
NEOTIDE_scRNA	This paper (Zenodo)	https://doi.org/10.5281/zenodo.11235504
**Software and algorithms**		
DRAGEN Bcl Convert pipeline v3.7.4.	Illumina sequencing system	https://support.illumina.com/sequencing/sequencing_software/dragen-bio-it-platform/downloads.html
GraphPad Prism	GraphPad Software, La Jolla California, USA	www.graphpad.com

(*Continued on next page*)

Continued

REAGENT or RESOURCE	SOURCE	IDENTIFIER
Fastp (Version 0.23.0)	Chen et al.[51]	RRID:SCR_016962
ANNOVAR	Wang et al.[52]	RRID:SCR_012821
SnpEff (Version 3.6)	Cingolani et al.[53]	RRID:SCR_005191
R (Version 4.0)	R Development Core Team, 2008	http://www.r-project.org
SPSS (Version 25.0)	IBM SPSS Statistics, New York, USA	https://www.ibm.com/cn-zh/products/spss-statistics
Python (Version 3.9.13)	Python 3 Reference Manual	https://www.python.org/
SOPRANO tool	Zapata et al.[54]	http://github.com/luisgls/SOPRANO
GISTIC 2.0	Mermel et al.[55]	https://www.genepattern.org/modules/docs/GISTIC_2.0
WGD	Bielski et al.[56]	https://github.com/taylor-lab/GD
CIN	Burrell et al.[57]	https://github.com/oicr-gsi/sequenza
Aneuploid	Shukla et al.[58]	https://github.com/pascalduijf/CAAs_1
OptiType	Szolek et al.[59]	https://github.com/FRED-2/OptiType
NetMHCpan4.0 software	Jurtz et al.[60]	https://services.healthtech.dtu.dk/services/NetMHCpan-4.0/
PROPHET	Chen et al.[61]	https://github.com/bnr-cdx/prophet
BWA (Version 0.7.10)	Aligning sequence reads, clone	RRID:SCR_010910
CellRanger toolkit (Version 6.1.2)	10X Genomics	https://www.10xgenomics.com/support/software/cell-ranger
Seurat package (Version 4.3.0)	Hao et al.[62]	https://cran.r-project.org/web/packages/Seurat/
Harmony package (Version 0.1.1)	Korsunsky et al.[63]	https://cran.r-project.org/web//packages/harmony/
CopyKAT package (Version 1.1.0)	Gao et al.[64]	https://github.com/navinlabcode/copykat
CellphoneDB v4	Alonso et al.[65]	https://github.com/ventolab/CellphoneDB
GSVA	Hänzelmann et al.[66]	https://www.bioconductor.org/
**Other**		
10µl Pipette Tip	Biosharp	Cat # BS-10-T
125µl Pipette Tip	INTEGRA Biosciences	Cat # 4421
200µl Pipette Tip	ExCell Bio	Cat # CS015-0012
300µl Pipette Tip	INTEGRA Biosciences	Cat # 4431
1000µl Pipette Tip	ExCell Bio	Cat # CS015-0013
1.5 mL Centrifuge tube	Sangon Biotech	Cat #F607620-9001
384-well Flat Clear Bottom White Polystyrene TC-treated Microplates	Corning	Cat # 3765
Cell counting tablet	Bodboge	Cat # JSP-GM
Treated Culture Dishes	Corning	Cat # 430196
Qualified 384-well Polypropylene (PP) Microplate	Beckman Coulter Life Science	Cat #C74290
Aluminum Sealing Film For General Sealing Purposes	Corning(Axygen)	Cat # PCR-AS-200
Screw cap micro tube(0.5mL)	Sarstedt	Cat # 72.730.105
Cell-Free DNA BCT tube	Streck, La Vista, NE, USA	Cat # 230244
Eppendorf single channel pipette(2-20µL)	EPPENDORF	Cat # 3123000098
Eppendorf single channel pipette(20-200µL)	EPPENDORF	Cat # 3123000055
Eppendorf single channel pipette(100-1000µL)	EPPENDORF	Cat # 3123000063
VOYAGER Electronic Pipettes(2-50µL)	INTEGRA Biosciences	Cat # 4726
VOYAGER Electronic Pipettes(10-300µL)	INTEGRA Biosciences	Cat # 4723
Cell counter	BodBoge	Cat # JSY-SC-031N

(*Continued on next page*)

Continued

REAGENT or RESOURCE	SOURCE	IDENTIFIER
Constant Temperature Mixer	Tuohe Electromechanical Technology	Cat # JXH-100+MD32
Mini Shaker	Kylin-Bell	Cat # MH-2
Luminoskan^{TM} Microplate Luminometer	Thermo Fisher Scientific	Cat # 5200330
Benchtop Centrifuge	EPPENDORF	Cat # 02-262-8187
Novaseq 6000 System	Illumina	Cat # 20012850
NovaSeq 6000 S4 Reagent Kit v1.5 (300 cycles)	Illumina	Cat # 20028312
NANO DROP	Thermo Scientific	Ca t# ND8000LAPTOP
LabChip GX Touch HT Nucleic Acid Analyzer	PerkinElmer	Cat # CLS137031

RESOURCE AVAILABILITY

Lead contact

Further information and requests for resources and reagents should be directed to and will be fulfilled by the lead contact, Wen-zhao Zhong (syzhongwenzhao@scut.edu.cn).

Materials availability

This study did not generate new unique reagents.

Data and code availability

All data generated in this study are included in this published article and its supplementary information files. The single-cell data that support the findings of this study are available from GEO: GSE241934. Bulk RNA data is available from GSA (HRA007419). Whole-exome sequencing (WES) data as well as tumor-informed ctDNA involving variant level data have been provided in Tables S9–S12. All other relevant detailed clinical and omics data could be available upon reasonable request from the lead contact Z.Zhong. No custom computer codes are reported in this paper. Codes used for scRNA-seq analysis are available from https://github.com/Anderson-Chaos/NEOTIDE_scRNA and on Zenodo (https://doi.org/10.5281/zenodo.11235504). Any additional information required to re-analyze the data reported in this work paper is available from the lead contact upon request.

EXPERIMENTAL MODELS AND STUDY PARTICIPANT DETAILS

Human subjects and ethical approval

Eligible patients were 18–75 years old with EGFR-mutant clinical stage IIB-IIIB (excluding N3) NSCLC (American Joint Committee on Cancer seventh edition criteria) which was considered as potentially resectable by multidisciplinary discussion team (MDT). All lesions should be measurable in terms of RECIST v1.1. EGFR mutation status should be confirmed before enrollment through either next generation sequencing (NGS) or PCR, and patients harbored either sensitive EGFR mutations (19del or 21L858R) or uncommon EGFR mutations (e.g., G719X, 20insertion, etc) were available to enroll. All patients had Eastern Cooperative Oncology Group performance status of 0 or 1 with normal organ function and no contra-indication to surgery. Patients who had an active, known or suspected autoimmune disease, or any underlying disease that investigators consider it may affect patient's prognosis including sever cardiovascular, pulmonary disease or serious infections were excluded. Besides, patients with active prior malignancy within the previous 5 years except for locally curable cancers that have been apparently cured, such as basal or squamous cell skin cancer, superficial bladder cancer, or carcinoma *in situ* of the prostate, cervix, or breast were excluded. Detailed inclusion and exclusion criteria could be found in supplemented study protocol.

The study was conducted in accordance with the clinical trial protocol and Good Clinical Practices Guidelines as defined by the International Conference on Harmonization and the Declaration of Helsinki. Written informed consent was provided by all study participants. The study was approved by Guangdong Provincial People's Hospital's institutional review board (KY-H-2022-009).

METHOD DETAILS

Study design

This is a phase 2, open-label, single-institution study (NCT05244213) conducted at Guangdong Provincial People's Hospital. All enrolled patients received 3 cycles of intravenous sintilimab 200mg, nab-paclitaxel 260 mg/m^2 and carboplatin AUC 5 at 3-week intervals. Surgery was scheduled within 3–6 weeks after last dose of neoadjuvant treatment. Since this study also included uncommon EGFR mutations, patients could choose either adjuvant EGFR-TKIs (prefer osimertinib) for no less than 2 years or intimate follow-up under investigators' recommendations. Patients were followed for at least 2 years after surgery.

All patients underwent baseline staging through enhanced PET-CT with or without enhanced intracranial MRI. Patients with suspected clinical stage N2 disease of station 2, 4 and 7 should be confirmed by either mediastinoscopy or EBUS. N1 disease could be determined through PET/CT but biopsy of primary lung cancer is needed. Routine scans were performed every 3 months during postoperative setting for up to 2 years after surgery which included thoracic CT scans at 3-month intervals and intracranial MRI at 6-month intervals or any relevant central nerve system (CNS) symptoms occurred. If patients were radiologically confirmed disease recurrence, whole-body PET-CT and biopsy of recurrence lesions should be performed if available.

The primary endpoint was MPR (defined as no greater than 10% viable tumor) rate for all treated participants. The secondary endpoints included pCR, event-free survival (EFS), objective response rate (ORR) (evaluated by the experienced medical oncologist through RECIST 1.1), overall survival (OS) and safety profile for all treated patients. Exploratory endpoints were surgical outcome for which per-protocol (PP) cohort would be analyzed including, but not limited to delayed or canceled surgery, surgical approaches and intraoperative/postoperative complications, and biomarkers analysis of correlation of genomic and immune profiling with pathological response and survival if corresponding data was mature. All treated patients were monitored for adverse events (AEs) according to the National Cancer Institute Common Terminology Criteria for Adverse Events, v5.0. Details of mandatory laboratory and physical tests at baseline, during neoadjuvant period and pre-surgery were noted in the study protocol.

Sample size justification

Simon's optimal two-stage design was applied for the study. Overall, 35 participants will be enrolled for the study. For stage I assessment, 18 participants should be enrolled and that at least 3 participants achieved MPR would allow to initiate stage II enrollment or terminate otherwise. If greater than 6 out of 35 participants had achieved MPR, the study would be deemed as positive and would be further evaluated in a randomized trial to verify the clinical superiority of this treatment modality in selected EGFR-mutant early-stage NSCLC. The sample size of the study is calculated based on the primary endpoint of MPR. The unacceptable (null hypothesis) MPR for the study was 10% and the desirable (alternative hypothesis) MPR was 30% following investigators' determinations and historical data. When the MPR rate is 10%, the probability of early termination is 73.4% with an average sample size of 22.5. The type I error (alpha) was set as 5% and type II error (beta) was set as 10%, generating 90% power.

Pathological assessment

Pathological assessment included gross examination and histopathological assessment of the resected lung specimens as well as lymph nodes. Surgeons should provide gross identification and mark the general tumor or tumor bed, which will then be sent to pathology department for further assessment. Pathological assessment will be performed by the experienced pathologist (L.X.Y) in regard of IASLC Multidisciplinary Recommendations for pathological assessment. At least one section per centimeter of greatest tumor (bed) diameter was used for histopathological evaluation. For primary tumors less than or equal to 3 cm in size, the entire tumor bed will be sampled for pathological evaluation. If tumors larger than 3 cm in size, at least one 0.5 cm-thick section would be submitted for review. The pathological assessment consisted three components including: 1) viable tumor cells 2) necrosis 3) stroma. Resected mediastinal and hilar lymph nodes were all submitted for microscopic assessment to determine whether there were residual metastatic tumor cells or not. Tumors with less than or equal to 10% of viable tumor cells in primary lung cancer were considered as MPR, and tumors with no viable tumor cells in both primary lung cancer and lymph nodes were considered as pCR. Pathological staging was performed based on primary lung cancer and lymph nodes of the resected specimens. To be noticed, part of the resected specimens that was sent out for experiments analysis will also be assessed pathological response in case of MPR overestimation.

PD-L1 assessment through IHC

FFPE tumor tissues were used to perform IHC analysis for PD-L1 (Dako 22C3, pharmDx). PD-L1 expression was quantified as the proportion of PD-L1-positive tumor cells. Positive PD-L1 expression in a given specimen was defined as $\geq$1% for tumor cell and $\geq$50% for high expression. Cases with <100 total tumor cells for scoring were defined as not applicable (NA).

DNA extraction and quality control

Tumor tissue acquired from surgical procedures or biopsies was processed as formalin-fixed and paraffin-embedded (FFPE) sections, with the tumor fraction for each sample determined via hematoxylin and eosin staining. Genomic DNA (gDNA) was extracted from tumor tissues with a pathological tumor cellularity of at least 30%, utilizing either the QIAamp DNA FFPE tissue kit from Qiagen (Hilden, Germany) or the MagPure FFPE DNA Kit (High Pure) from Magen (Guangzhou, China). Matched genomic DNA was extracted from EDTA-anticoagulated peripheral whole blood or buffy coat samples using the MagPure Universal DNA Kit (Magen, Guangzhou, China), following the manufacturer's instructions. DNA concentration was quantified using the Qubit dsDNA HS assay from Thermo Fisher (Carlsbad, CA, US). For circulating cell-free DNA (cfDNA), approximately 10 mL of peripheral blood sample was collected and preserved in Cell-Free DNA BCT tubes (Streck, La Vista, NE, US). Within 72 h of collection, the sample was centrifuged at 2,000 g and 4°C for 10 min. The supernatant was transferred to a 15 mL centrifuge tube and centrifuged at 16,000 g and 4°C for 10 min. Subsequently, the supernatant was transferred to a new tube and stored at −80°C until further use. cfDNA was recovered from 4 to 5 mL of plasma by using the QIAamp Circulating Nucleic Acid kit or QIAsymphony DSP Circulating DNA Kit (Qiagen, Hilden, Germany). Quantification of cfDNA was conducted using the Qubit dsDNA HS assay (Thermo Fisher, Carlsbad, CA, US).

CellPress
OPEN ACCESS

Whole exome library preparation, sequencing and analysis platform

The library preparation for whole-exome sequencing (WES) was carried out using Twist Human Core Exome kit (Twist Bioscience, South San Francisco, CA, US), following the manufacturer's recommendations. Briefly, FFPE DNA samples and paired white blood cell (WBC) gDNA samples were fragmented to peak size 200 bp using M220 Focused-ultrasonicator (Covaris, Woburn, MA, US). The fragmented DNA samples underwent end-repaired and dA-tailed, followed by ligation with Universal Adapters. After post-ligation purification, the ligated products were polymerase chain reaction (PCR) amplified with index primers. The number of amplification cycles used during library preparation varied according to the manufacturer's recommendations. Exome capture was performed utilizing Twist Fast Hybridization and Wash Kit, using a 33 Mb Human Core Exome panel and a customized-designed supplementary panel. Up to 8 libraries were multiplexed in one capture reaction, with each library requiring 400 ng of input. The Qubit dsDNA HS assay (Thermo Fisher, Carlsbad, CA, US) was used to quantify the final libraries. Following library size-distribution determination using the LabChip GX Touch System, the libraries were sequenced on a NovaSeq 6000 sequencer (Illumina, San Diego, CA, US), generating 2 × 151 bp pair-end reads with unique dual index, with a mean target coverage of 500× for tumor samples and 150× for paired normal samples.

WES data analysis was performed using Illumina DRAGEN Bio-IT Platform (Illumina, Inc., San Diego, CA, US) unless otherwise indicated. FastQ files were generated from raw BCL data using the DRAGEN Bcl Convert pipeline v3.7.4. Adapters were trimmed using fastp version 0.23.0,[51] and reads with a length less than 50 bp were discarded. Clean reads were aligned to the human reference genome (NCBI GRCh37; hg19).[67] Initial sequencing and analysis of the human genome and PCR duplicates were marked for downstream analysis filtering. As a quality-control (QC) process for all samples captured by the Human Core Exome panel, tumor and paired normal alignments were checked for multiple QC parameters using the in-house software to assess capture efficiency, coverage uniformity, and library complexity. Single nucleotide variants (SNVs) and insertions/deletions (INDELs) were called if the variant supporting reads were at least 5, and the mutation variant allele frequency (VAF) was at least 3%. Variants with a population frequency equal to or greater than that found in ExAC, 1000 Genomes, dbSNP, and ESP6500SI-V2 databases were excluded. The ratio of tumor allele frequency (AF) to paired-normal AF was calculated for each variant in order to obtain authentic somatic mutations. Variants with a ratio <3 or both tumor and paired-normal AFs >10% were excluded. All variants passing the applied filters were annotated using ANNOVAR[52] and SnpEff version 3.6.[53]

Whole exome data analysis

Statistical analyses were conducted using R version 4.0 (http://www.r-project.org). The SOPRANO tool (http://github.com/luisgls/SOPRANO) was built based on the previously published method by Zapata et al.[54] It calculates selection in variant effect predictor annotated files, focusing on negative selection in tumor genome evolution acting on essential cellular functions and the immunopeptidome. The dN/dS ratio was computed both inside (ON-target dN/dS) and outside (OFF-target dN/dS) of a target region using SSB192, a 192-trinucleotide context correction. We utilized GISTIC 2.0[55] to identify somatic copy number alterations (sCNAs) that could distinguish the MPR group from the non-MPR group. Indicators related to genomic instability were calculated using an in-house algorithm, incorporating whole genome doubling (WGD),[56] CIN[57] and aneuploidy.[58] Sequenza (R package, (http://www.cbs.dtu.dk/biotools/sequenza/)) and scarHRD (https://github.com/sztup/scarHRD) were employed to calculate indicators related to homologous recombination deficiency (HRD). HLA prediction was performed using OptiType[59] (https://github.com/FRED-2/OptiType) and NetMHCpan4.0 software[60] for epitope binding predictions. The R package deconstructSigs 1.8.0 identifies signatures present in a tumor sample. Tumor neoantigen burden (TNB) value is the total number of peptides with predicted affinity less than 500 nM. Tumor mutational burden (TMB) was presented as the ratio between the total number of nonsynonymous mutations and the panel size of WES kit.[62]

Whole transcriptome sequencing and analysis

RNA was isolated from FFPE samples using an Magpure DNA/RNA FFPE Kit (Magen, Guangzhou, China). The quantity and quality of extracted RNA was quantified by NANO DROP (Thermo Fisher Scientific, Waltham, MA, USA) and LabChip GX Touch HT Nucleic Acid Analyzer (PerkinElmer, Waltham, MA, USA), respectively. Fragmented RNA was subjected to strand-specific cDNA synthesis, followed by dA-tailing, unique molecular identifier (UMI) adaptor ligation, PCR amplification, and hybridization with capture probe baits. The prepared NGS libraries were sequenced on a NovaSeq 6000 system (Illumina, Inc., San Diego, CA, USA). A threshold of >25 million reads per sample was set. After deduplication and removing UMI from the sequence header, adaptors, and low-quality reads were removed. The cleaned reads were aligned to the human reference genome 19 by STAR (2.7.3a)., then the consensus reads were created using homebrew software based on UMI sequence and read alignment position. Consensus reads were aligned again to the human reference genome 19 by STAR2 (2.7.3a).

Patient derived organoids (PDOs) establishment

Human lung cancer tissues were washed twice in cold D-PBS and minced into small pieces with a scalpel, then transferred to 5 mM EDTA in PBS for 15 min at room temperature, and digested in 1 mM EDTA in TrypLE for 1 h at 37°C with agitation. The pieces were collected in cold Advanced DMEM/F12, further dissociated to obtain tissue cell suspension and filtered through a 70 μm cell strainer to remove large debris. Cell pellets were centrifuged at 300g for 5 min after washed with cold D- PBS and resuspended with cold Matrigel. Three drops (10000 cells/drop) of 30ul mixture were plated into one well of a 12-well plate. After drops were solidified at

the incubator for 10 min, 1 mL pre-warmed human lung organoid medium (Advanced DMEM/F12(12634028; Thermo Scientific) supplemented with N2 (1750202; Thermo Scientific), B27 (17504044; Thermo Scientific), penicillin/streptomycin (15140122; Thermo Scientific), Antibiotic-Antimycotic (100 U/ml; Thermo Scientific), HEPES (10 mM; Thermo Scientific), GlutaMax (2mM; Thermo Scientific), N-acetyl-L-cysteine (1.25 mM; Sigma-Aldrich), Primocin (50μg/ml; Invitrogen) containing Noggin (100 ng/mL; R&D), R-spondin 1 (500 ng/mL; R&D), A83-01 (0.5 μM; Cayman), Y-27632 (5 μM; Selleck), FGF-7 (25 ng/mL; Novoprotein), FGF-10 (100 ng/mL; Novoprotein), SB202190 (5μM; Selleck), Nicotinamide (5 mM; Sigma-Aldrich)) were added. The medium was replaced every 4 days and organoids were passaged at 1:2-1:3 every 2 weeks.

Drug treatment and sensitivity tests

The well-cultured organoids are harvested from the Matrigel matrix, washed with PBS, and centrifuged to remove the supernatant. They are then resuspended in an appropriate volume of TrypLE digestion solution and incubated at 37°C for gentle digestion. The organoids are dispersed into single cells by gentle pipetting, followed by centrifugation to remove the supernatant. The cells are resuspended in PBS, mixed with an equal volume of 2% Trypan Blue for cell counting, and centrifuged again to remove the supernatant. The cell pellet is resuspended in culture medium containing 5% Matrigel, and the cells are seeded in a 384-well plate at a volume of 30μL per well. The plate is incubated in a 37-degrees Celsius, 5% CO2 cell culture incubator. After 24 h of incubation, the test compounds including targeted drugs and chemotherapy are introduced. Following a 5-day incubation period with the compounds, the ATP detection reagent is added. After shaking and centrifugation, the cells are incubated at room temperature for 25 min, and cell viability is assessed using a chemiluminescence reader. Finally, the data is analyzed using GraphPad.

Circulating tumor DNA (ctDNA) analysis

ctDNA analysis was conducted using a tumor-informed personalized ctDNA panel based on whole-exome sequencing (PROPHET, Burning Rock Biotech, Guangzhou, China). The PROPHET algorithm for measuring ctDNA was fully introduced in the previous publication.[61] In brief, patient-specific somatic variants were identified through the analysis of WES data obtained from the primary tumor and matched normal white blood cell. For a given set of variants, up to 50 variants with the highest Variant Allele Frequency (VAF) and VAF ≥ 3.0% were selected to construct the personalized panel. The biotinylated capture probe pool was created in-house, tailored to each individual's personalized panel. The library preparation and enrichment process were carried out using the Burning Rock HS unique molecular identifier (UMI) library preparation kit. Ultra-deep UMI-based sequencing was performed on a NovaSeq 6000 platform (Illumina, San Diego, CA, US), generating 2 × 151bp paired-end reads, with a target raw depth of 100,000x. The ctDNA fraction in plasma samples was estimated using the maximum likelihood (ML) method based on monitoring multiple loci, as previously established. Initially, PROPHET assumed that the mutation rate at each site followed a Poisson distribution to calculate the significance of each informed mutation. Sites with a p-value <0.05 were considered as significant sites. Subsequently, a likelihood ratio test was applied to determine whether the ctDNA fraction in the sample was significantly greater than 0, and the sample-level p-value was calculated. Ultimately, the ctDNA positive status was defined as having two or more significant sites and a sample-level p-value <0.005.

Flow cytometry (FACS) and multiplex cytokines of peripheral samples

Peripheral blood mononuclear cells (PBMCs) were stained at a maximal concentration of $5*10^6$ cells/mL in staining buffer in the dark (PBS, 2% FCS, 1mM EDTA) for 20min. All the procedures were performed upon standard manufacturing of FACS. Cell surface markers were stained with the corresponding antibodies identified in the key resources table. For multi-color flow cytometric surface marker analysis, cells were stained for 30 min in the dark. For multiplex cytokines detection, a commercial kit (AimPlex) identified in the key resources table was used for multiple cytokines detection including IFN-γ, IL-1b, IL-2, IL-4, IL-5, IL-6, IL-8, IL-10, IL-12p70, IL-17A, IL17F, IL22, TNF-α and TNF-β.

Tissue dissociation and scRNA-seq

Fresh resected tissues were collected within half an hour after surgical resection and kept in MACS Tissue Storage Solution (Miltenyi Biotec) until processing. Samples were washed with RPMI-1640 medium (Gibco, 11875093), cut into small pieces of approximately $1mm^3$ on ice and enzymatically digested with Tumor Dissociation Kit (Miltenyi, 130-095-929) for 60 min on a rotor at 37°C. Dissociated cells were subsequently sieved through a 100μm MACS SmartStrainers (Miltenyi, 130-110-917) and centrifuged at 300g for 8 min. After removing the supernatant, pelleted cells were suspended in red blood cell lysis buffer (TIANDZ, 90309-100) and incubated on ice for 5 min to lyse red blood cells. After washing with PBS (Gibco, 10010023), cell pellets were re-suspended in sorting buffer (PBS supplemented with 2% FBS). Single-cell suspensions were then stained with 7-AAD Viability Staining Solution (Invitrogen, 00-6993-50) and an antibody against CD235a (BioLegend, 349114) for FACS sorting on a BD Aria III instrument. Non-erythrocytic living cells were enriched by gating 7AAD−CD235a− cells. The concentration of single-cell suspensions was then adjusted to 700~1,200 cells/μL^{-1} and single-cell suspensions with viable cell rate greater than 80% were used for single cell sequencing. In total, ~18,000 cells for each sample were used for 10X Chromium Single cell 5′ and human TCR library construction (10X Genomics), according to the manufacturer's instructions. All subsequent steps were performed following the standard manufacturer protocols. Purified libraries were subsequently sequenced using an Illumina NovaSeq 6000 sequencer with 150-base pair (bp) paired-end reads.

Single-cell RNA sequencing data processing

The raw count matrix was obtained using the CellRanger toolkit (version 6.1.2), employing GRCh38 as the reference genome. We then analyzed the combined count matrix using the Seurat package (version 4.3.0).[62] Only cells fulfilling the following three quality control metrics were preserved for subsequent analyses: (1) a total UMI count between 1000 and 40000, (2) a detected gene count ranging from 600 to 5000, and (3) a mitochondrial gene count proportion under 10%. Cells expressing marker genes spanning two major cell types were regarded as potential doublets and subsequently filtered out before further analyses. Additionally, genes detected in fewer than 200 cells were excluded. We executed read count normalization and variable feature selection using the Seurat package. The top 2000 variable genes were selected for principal component analysis (PCA), and the first 20 PCs were used for downstream analysis. We used RunHarmony function in the Harmony package (version 0.1.1)[63] to correct batch effect between patients.

Clustering and identification of cell types

The Louvain algorithm, implemented in the FindCluster function, was used to identify clusters. To reduce dimensionality and visualize single cells in a two-dimensional space, we performed UMAP using the RunUMAP function with the first 20 principal components. Major single-cell lineages were identified according to the following canonical marker genes: T/NK (CD3D, CD3E, NKG7), Myeloid (CD68), B (CD79A), Mast (KIT, TPSAB1), Endothelial (VWF, PECAM1), Epithelial (EPCAM, KRT19), Fibroblast (COL1A1, COL6A1). We replicated this process for cells within each major lineage of immune cells to identify subpopulations. After multiple rounds of clustering to optimize resolution, we identified 22 T/NK cell clusters, 9 B cell clusters, and 15 myeloid cell clusters.

Single-cell TCR data processing and analysis

We utilized CellRanger(version 6.1.2) for TCR-seq read alignment and assembly. Our analysis included only the productive, highly confident, and full-length TCR sequences assigned with a valid cell barcode and an unambiguous chain type. Clonal cells were characterized as those having identical alpha/beta-chain pairs, indicating the same origin and antigen specificity. The size of each clonotype was calculated based on the aforementioned definitions. To quantify different expansion levels of T cell subtypes, we defined expanded ratio of a cell type as number of cells in expanded clonotype($\geq$ 3 cells in a clonotype) in one cell type divided by total number of cells in that cell type. We defined expanded CXCL13+Tex clones as clonotype containing T cells annotated as CD8T_Tex_CXCL13 with a clone size of $\geq$ 10 cells. For Treg cells, expanded CCR8+ Treg clones were defined as clonotype containing T cells annotated as CD4T_Treg_CCR8 with a clone size of $\geq$ 3 cells because Tregs were observed to be less expanded than CD8 T cells.

Gene module enrichment analysis

To explore functional differences between T cell clusters, we used AddModuleScore function with default parameters to quantify normalized gene set expression score for each cell. Gene sets used for module enrichment analysis could be found in Table S15.

Identification of malignant cells and differential expression analysis

We used the CopyKAT package (version 1.1.0)[64] to evaluate copy number variations (CNVs) within individual cells, thereby distinguishing malignant cells from normal epithelia. We used stromal cells, including fibroblasts and endothelia, as the normal reference for the algorithm. To mitigate potential algorithmic bias, we conducted the aforementioned processing and clustering procedure for all epithelial cells and identified clusters with high proportions of aneuploid cells and high CNV scores as potentially malignant clusters. CNV score was defined as the sum of absolute values of copykat predicted CNVs. Samples with fewer than 10 identified malignant cells were excluded prior to further analyses. To identify genes differentially expressed between highly resistant tumor cells and other tumor cells, we used the Wilcoxon rank-sum test, implemented in the FindAllMarkers function with the parameters "min.pct = 0.25, thresh.use = 0.25".

Cell-cell interaction analysis

To infer interaction between malignant cells and immune cells, we used CellPhoneDB[65] to identify potential ligand-receptor interactions. The significance of the interactions was given by permutation test (1000 times) while other parameters were remained as default.

External Validation using bulk RNA data

To validate our results, we used gene set variation analysis (GSVA)[66] to estimate the proportions of CCR8+Treg and CXCL13+Tex in external bulk RNA seq datasets. Markers of these two cell types (CCR8+Treg: FOXP3, IL2RA, CCR8; CXCL13+Tex: HAVCR2, PDCD1, LAG3, CXCL13, TIGIT, CTLA4, ENTPD1, LAYN) (Table S8) as the gene set for GSVA with the parameters “methods = ‘ssgsea’, kcdf = ‘Gaussian’”. The patients were classified into high and low signature group by the median value. Survival data as well as response rate, if available, was used to testify the immune signature.

QUANTIFICATION AND STATISTICAL ANALYSIS

SPSS software (version 25.0, IBM; Armonk, NY), GraphPad Prism (version 8.0, GraphPad Software; San Diego, Calif), and R software (version 4.0, R Foundation; Vienna, Austria) were used for statistical and survival analysis. Continuous variables are summarized as mean standard deviations or medians with full ranges. Categorical variables are summarized as frequencies and percentages. Continuous variables were compared using the Student's t test or Wilcoxon rank-sum test regarding specific conditions. Categorical variables were compared using the chi-square test or Fisher exact test. Kaplan Meier analysis was used to compare survival between groups and log rank test *p* value was calculated. All reported *p*-value were 2-tailed, and the statistical significance was defined as $p < 0.05$.

ADDITIONAL RESOURCES

This clinical trial has been registered on https://clinicaltrials.gov (NCT05244213).

Cell Reports Medicine, Volume *5*

Supplemental information

Neoadjuvant sintilimab plus chemotherapy in EGFR-mutant NSCLC: Phase 2 trial interim results (NEOTIDE/CTONG2104)

Chao Zhang, Yu-Xuan Sun, Ding-Cheng Yi, Ben-Yuan Jiang, Li-Xu Yan, Ze-Dao Liu, Li-Shan Peng, Wen-Jie Zhang, Hao Sun, Zhi-Yong Chen, Dan-Hua Wang, Di Peng, Song-An Chen, Si-Qi Li, Ze Zhang, Xiao-Yue Tan, Jie Yang, Zhang-Yi Zhao, Wan-Ting Zhang, Jian Su, Yang-Si Li, Ri-Qiang Liao, Song Dong, Chong-Rui Xu, Qing Zhou, Xue-Ning Yang, Yi-Long Wu, Ze-Min Zhang, and Wen-Zhao Zhong

Supplementary Figure

Supplementary Fig.1 Clinical and multi-omics exploratory study design of CTONG2104 (NEOTIDE), related to Figure 1 and STAR Methods. Tx, treatment; EGFR, epidermal growth factor receptor; ECOG, Eastern Cooperative Oncology Group; MPR, major pathological response; pCR, pathological complete response; ORR, objective response rate; EFS, event-free survival; OS, overall survival; CN, chemotherapy; WES, whole-exome sequencing; WBC, whole blood cells; MRD, minimal residual disease.

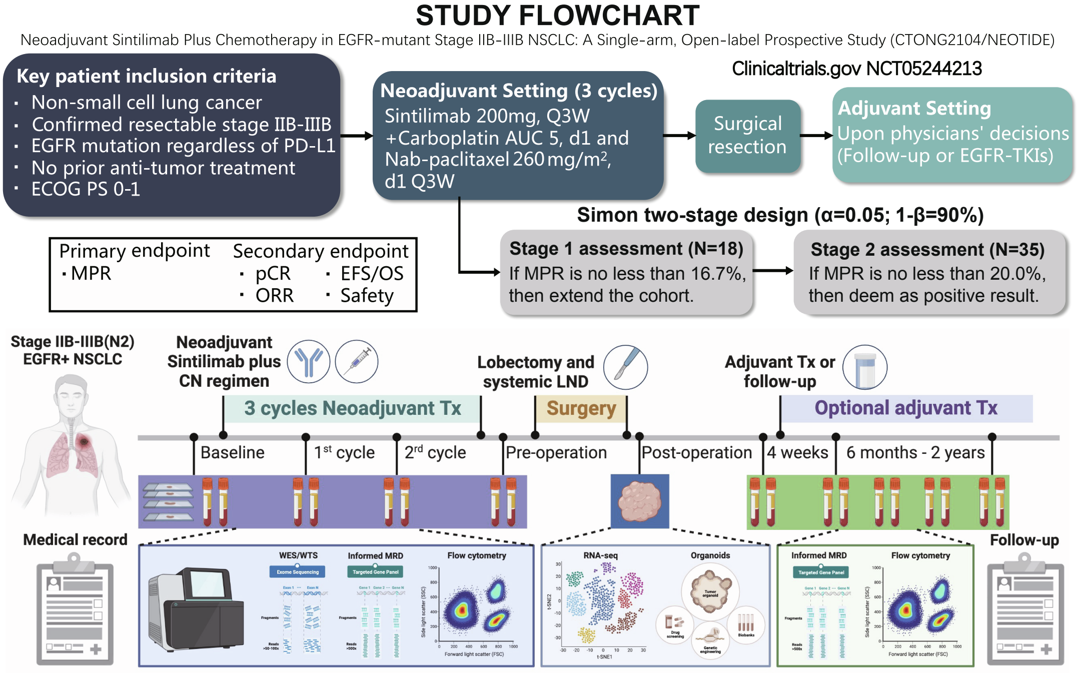

Supplementary Fig.2 Pathological response evaluation procedures, related to Figure 2 and STAR Methods. a Process of tumor sampling, preparation and assessment. **b** Schematic of counted components during pathological assessment including viable tumor, necrosis and stroma. **c** Specific pathological evaluation across different slides in a representative case. Red circles indicated viable tumor area. PLC, primary lung cancer; DLN, draining lymph nodes; RVT, residual viable tumor.

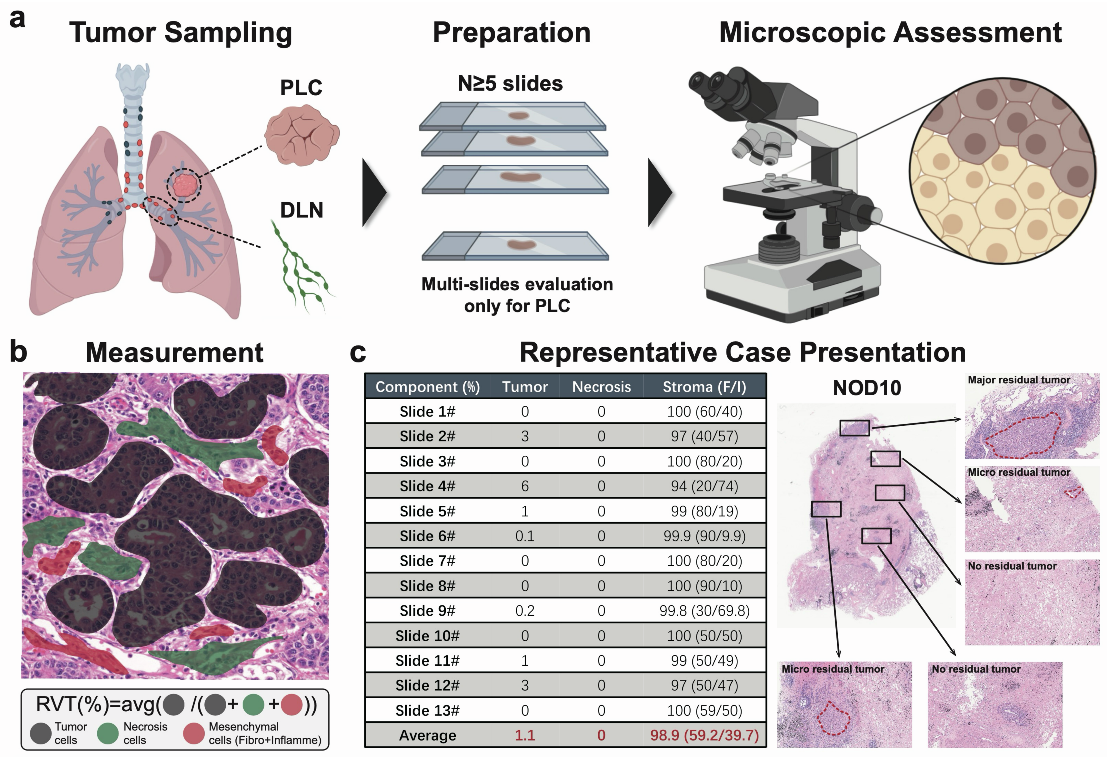

Component (%)	Tumor	Necrosis	Stroma (F/I)
Slide 1#	0	0	100 (60/40)
Slide 2#	3	0	97 (40/57)
Slide 3#	0	0	100 (80/20)
Slide 4#	6	0	94 (20/74)
Slide 5#	1	0	99 (80/19)
Slide 6#	0.1	0	99.9 (90/9.9)
Slide 7#	0	0	100 (80/20)
Slide 8#	0	0	100 (90/10)
Slide 9#	0.2	0	99.8 (30/69.8)
Slide 10#	0	0	100 (50/50)
Slide 11#	1	0	99 (50/49)
Slide 12#	3	0	97 (50/47)
Slide 13#	0	0	100 (59/50)
Average	1.1	0	98.9 (59.2/39.7)

Supplementary Fig.3 Correlation of MPR status with clinicopathological features and peripheral blood test, related to Figure 2 and STAR Methods. a Associations between clinicopathological characteristics (smoking, staging, EGFR subtypes and PD-L1 levels) and pathological response. Wilcoxon-rank sum test was used to measure the significance. **b** Comparison between relevant clinical factors and MPR status where no significant difference was found. **c** Flowcytometry analysis of PBMC regarding major cell lineage before and after neoadjuvant treatment. T test was used to measure the significance. **p<0.01, ***p<0.001. **d** Dynamic changes of $CD3^{+}CD4^{+}$T cells and $CD19^{+}$B cells across different time points. Two-way ANOVA was used to calculate the significance of each time point between MPR and non-MPR, and Wilcoxon signed rank test was used to measure the significance between baseline and pre-surgery within each group. **e** Comparison of plasma IL6/IL8 before and after neoadjuvant immunochemotherapy. Wilcoxon signed rank test was used to measure the significance. **f** Difference of plasma IL8 before and after neoadjuvant treatment in MPR and non-MPR cohort, respectively. PRR, pathological regression rate; NLR, neutrophil/lymphocyte ratio; LMR, lymphocyte/monocyte ratio; MPR, major pathological response; PBMC, peripheral blood mononuclear cell.

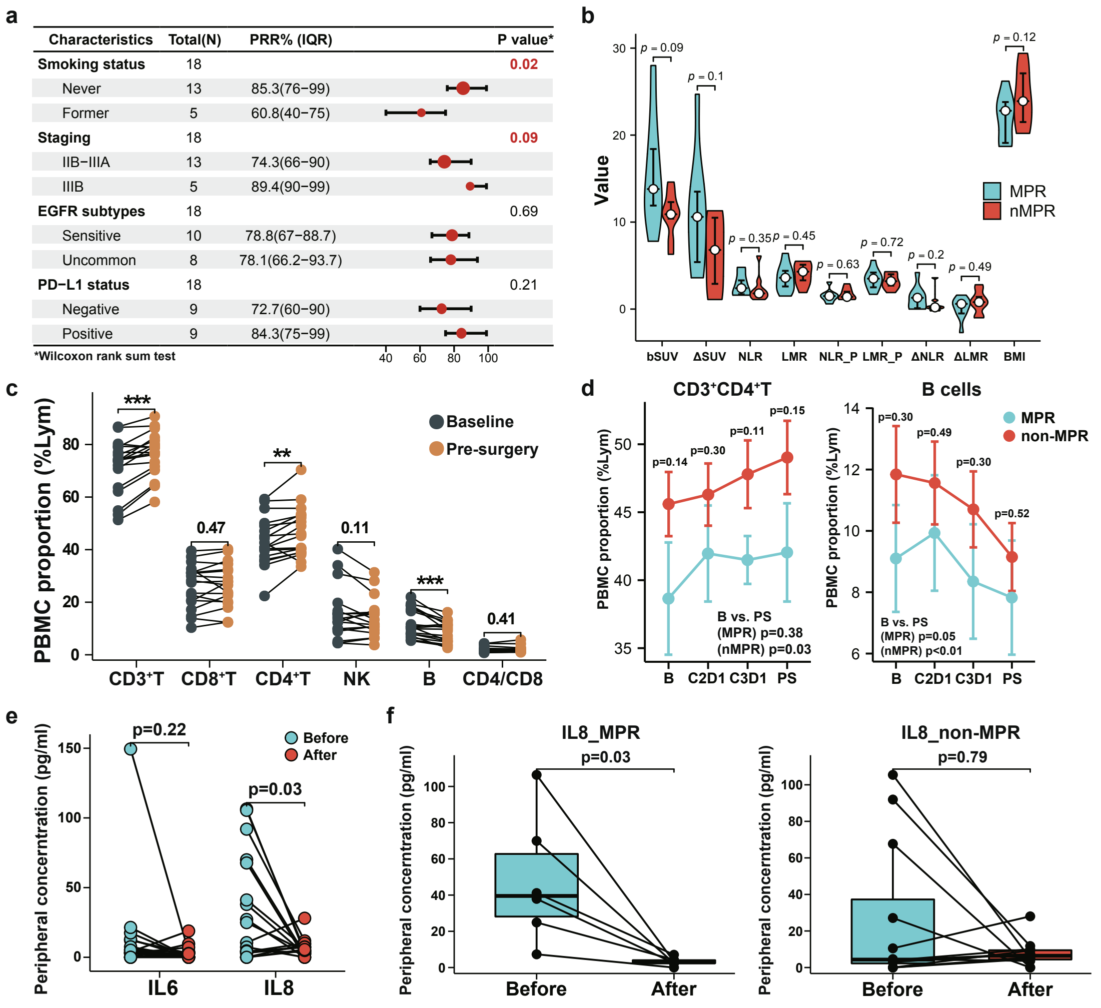

Supplementary Fig.4 Correlation of genomic features and pathological response, related to Figure 3 and STAR Methods. a Comparison of TP53 subtypes between MPR and non-MPR patients. Fisher exact test was used to measure the significance. **b** Relative abundance of preferable altered mutations in MPR or non-MPR patients with annotated genes correlated with TKIs (red) or immune response (green). **c** Pathway enrichment of MPR and non-MPR patients through differential genes. MPR, major pathological response; TKI, tyrosine kinase inhibitor.

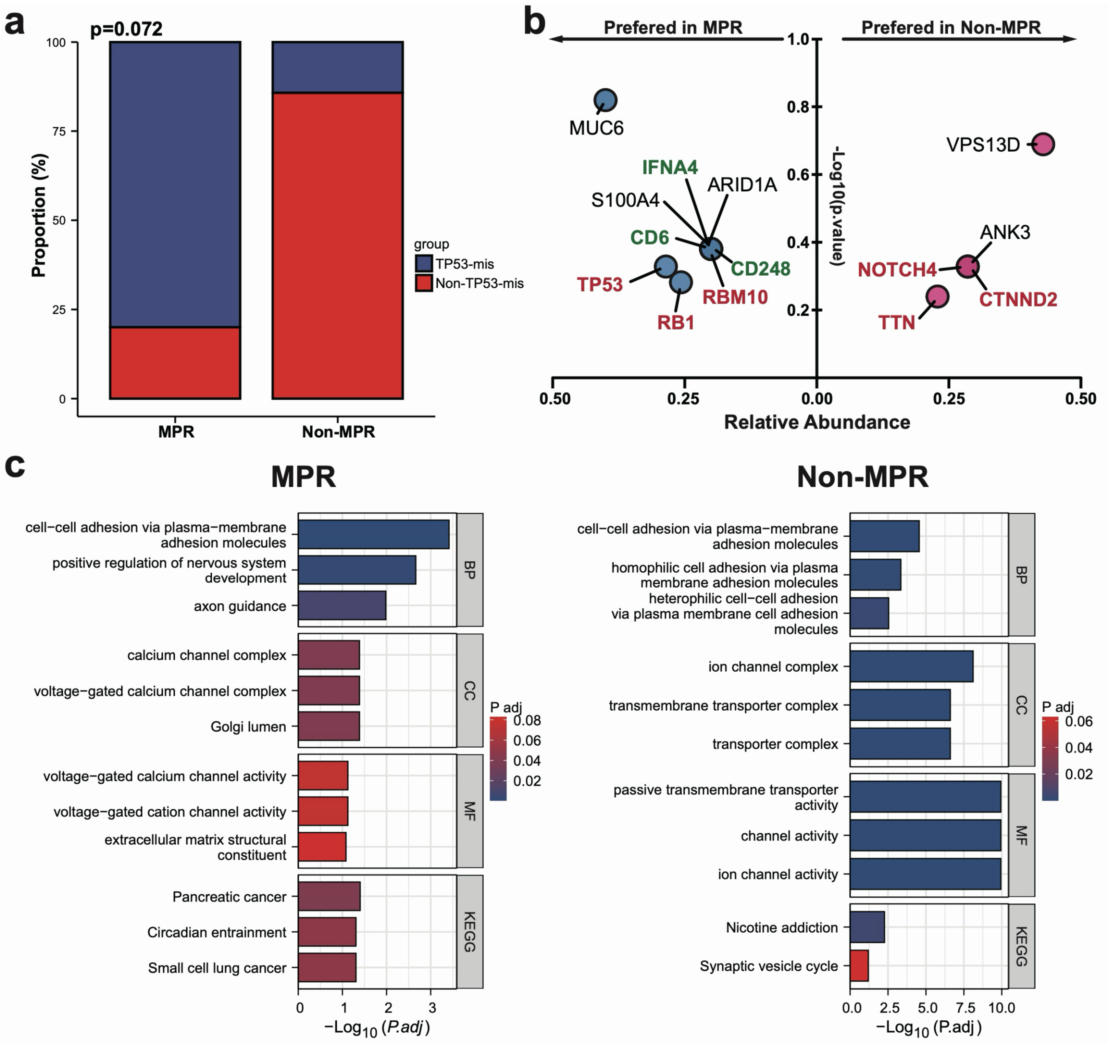

Supplementary Fig.5 Establishment of PDOs for residual tumor and susceptibility test, related to Figure 3 and STAR Methods. a Verification of PDOs through HE and IHC staining for two patients. Staining from both origin tumor and PDOs were matched. **b** Comparison of genomic features showed relatively similar CNVs between origin tumor and PDOs as well as specific EGFR mutation subtypes. **c** Anti-tumor susceptibility test suggested superior efficacy of second-generation EGFR-TKIs followed by the third generation EGFR-TKI. IC50 of each drug was presented along with AUC value. PDOs, patient-derived organoids; HE, hematoxylin-eosin; IHC, immunohistochemistry; IC50, half maximal inhibitory concentration.

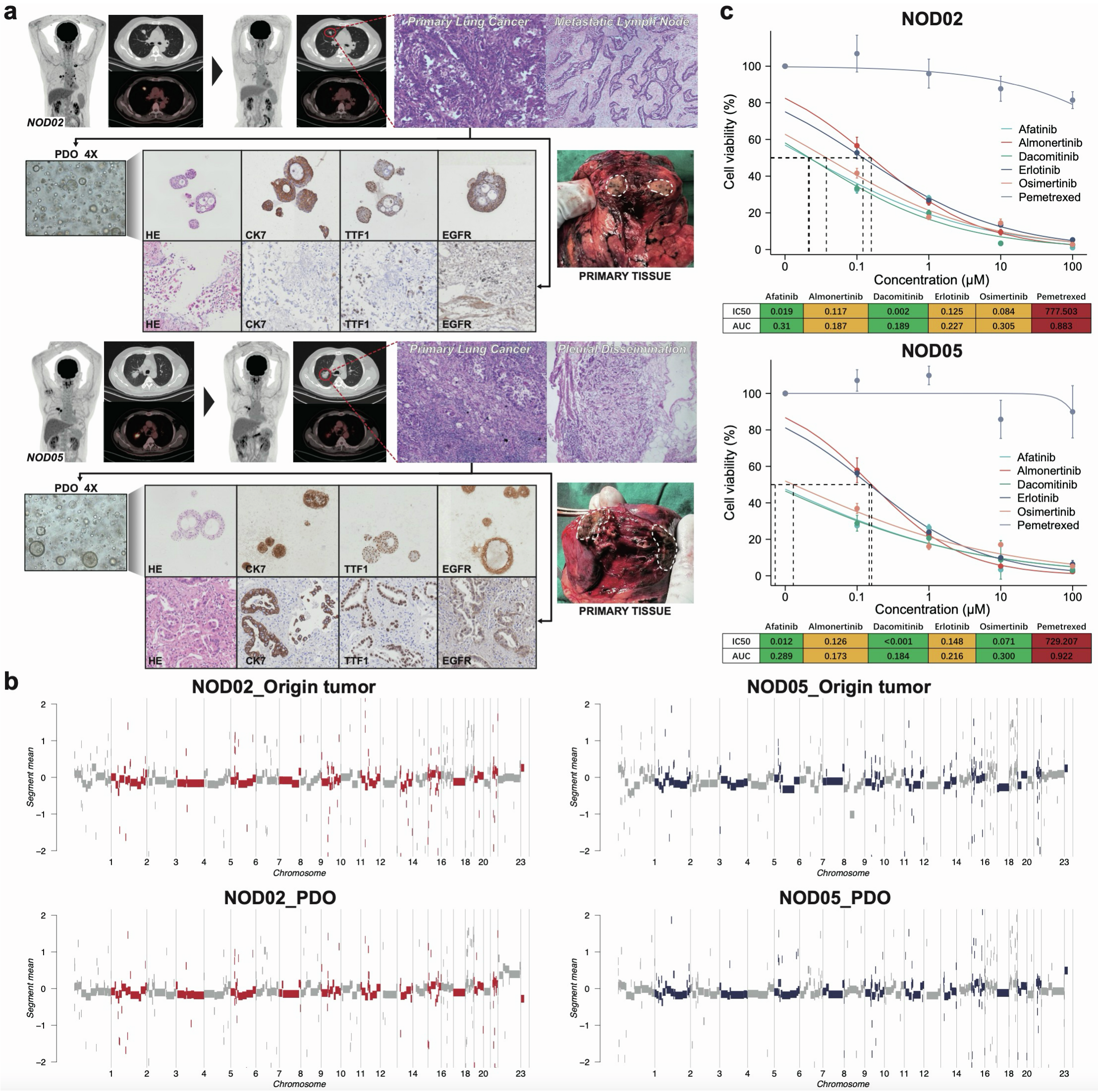

Supplementary Fig.6 Other immune-relevant genomic features regarding WES, related to Figure 3 and STAR Methods. Comparison of WGD(**a**), Ploidy(**b**), HLA-LOH(**c**), TMB(**d**), TNB(**e**) and CIN(**f**) between MPR and non-MPR patients (N=12). Fisher test was used to calculate the significance for categorical variables and T test was used for continuous variable. **g** Immune selection measured through SOPRANO algorithm among different individuals. No apparent difference was found between groups. WGD, whole genome doubling; HLA-LOH, human leukocyte antigen loss of heterozygosity; TMB, tumor mutation burden; TNB. Tumor neoantigen burden; CIN, chromosomal instability; dN/dS, nonsynonymous to synonymous mutations ratio.

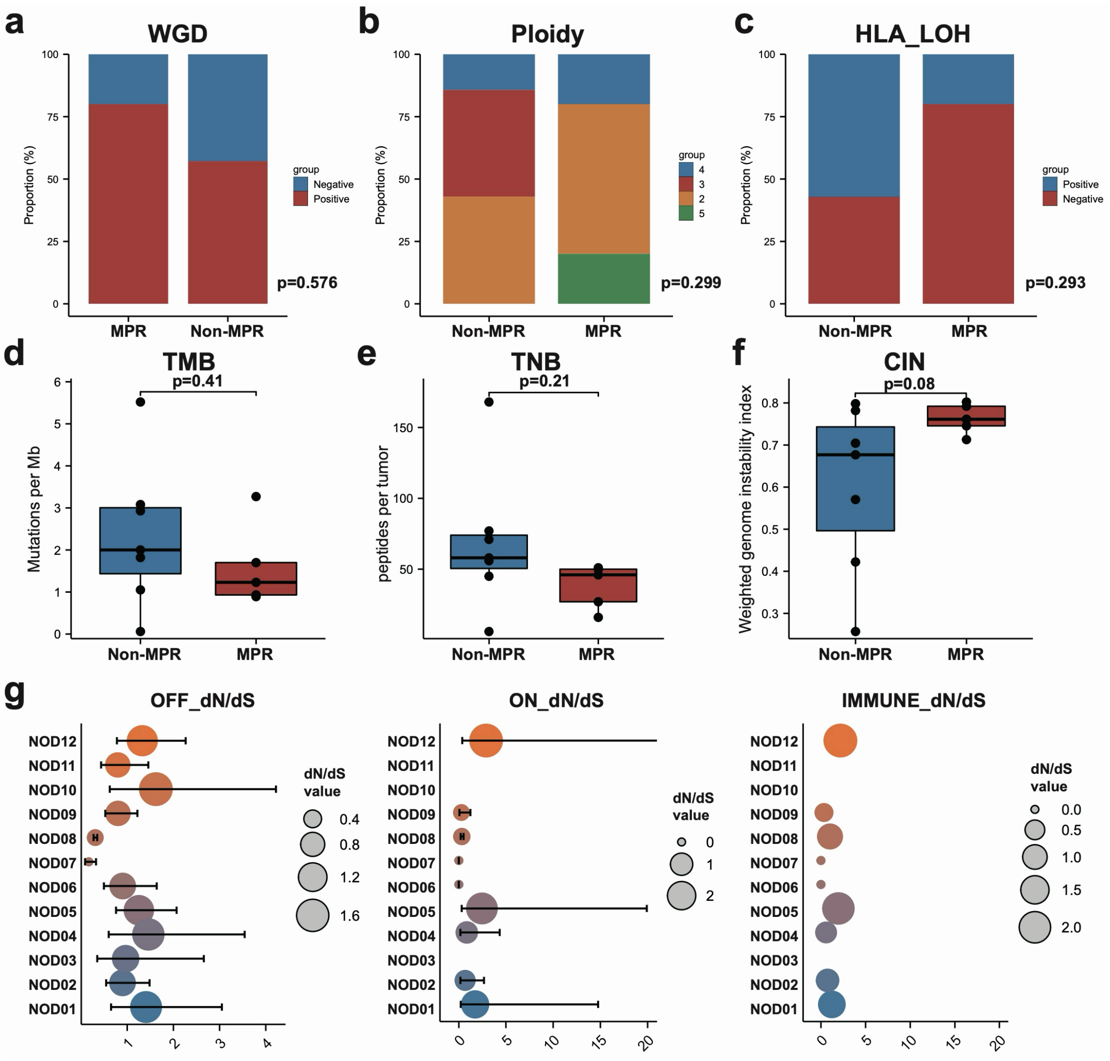

Supplementary Fig.7 Flowchart of ctDNA acquisition and dynamic changes, related to Figure 3 and STAR Methods. a Acquisition of ctDNA in first 12 patients. 2 patients did not have adequate tumor for WES and used resected specimens to generate the customized ctDNA panel. **b** Positivity of all customized ctDNA levels and EGFR mutation only at baseline detected through tumor-informed MRD. EGFR KDD mutation derived from one patient could not be captured by WES technically, but large panel NGS confirmed EGFR KDD mutation in resected specimens after neoadjuvant treatment. **c** Percentage of ctDNA positivity across different time points. **d** Dynamic changes of ctDNA status and response outcome. **e** Comparison of MTM between MPR and non-MPR patients across different time points. Welch t' test was used to calculate the significance.

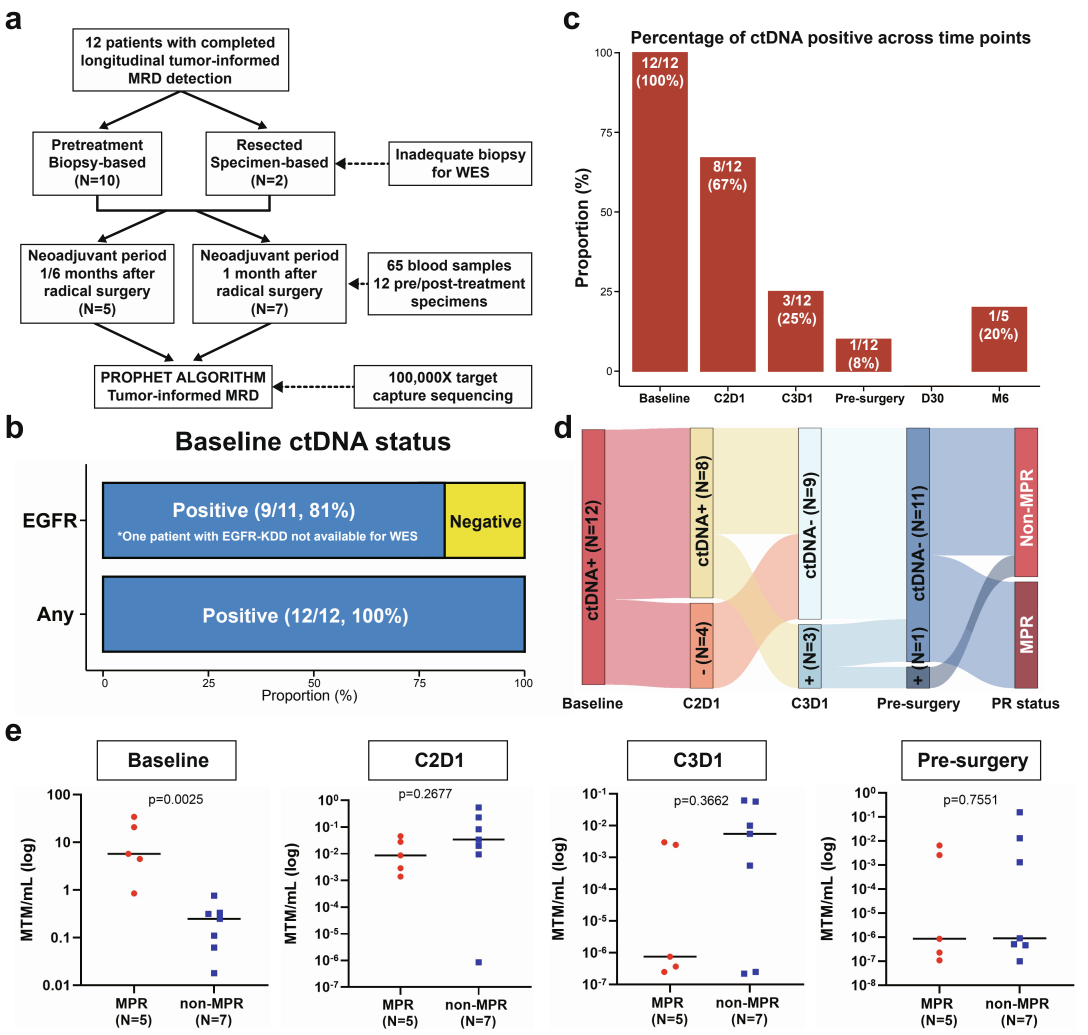

Supplementary Fig.8 Longitudinal monitoring of informed MRD detection, related to Figure 3 and STAR Methods. Each plot represented longitudinal changes of MRD individually along with their genomics features. LUAD, lung adenocarcinoma; LUASC, lung adeno-squamous carcinoma; EGFR, epidermal growth factor receptor; MRD, minimal residual disease; WGD, whole genome doubling; PL, ploidy; IMS, immune status; HLA-LOH, human leukocyte antigen loss of heterozygosity; TMB, tumor mutation burden; TNB, tumor neoantigen burden.

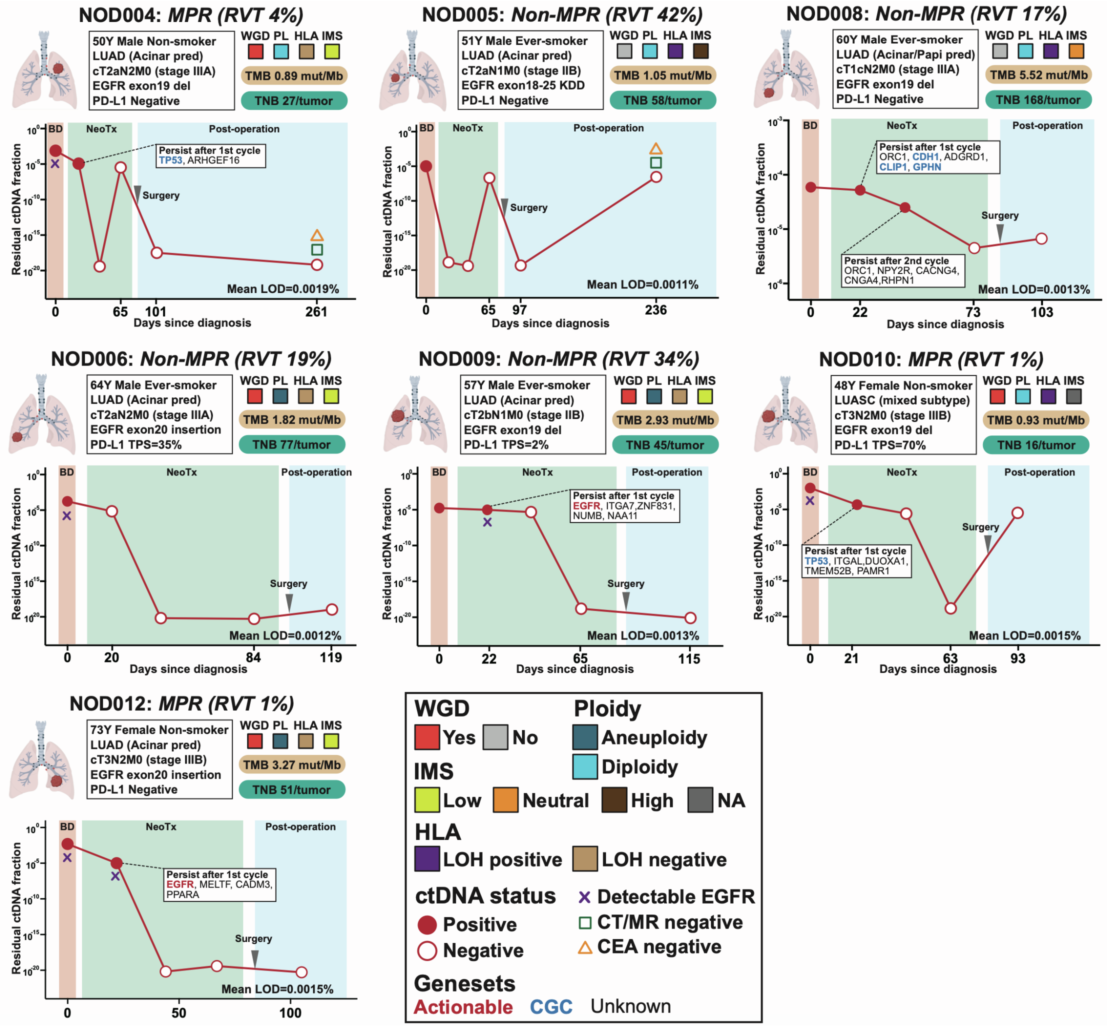

Supplementary Fig.9 Patients' characteristics of included cohorts and quality control of single-cell sequencing, related to Figure 4 and STAR Methods. a Baseline clinical features of NEOTIDE and real-world cohort. Only one patient from NEOTIDE cohort did not have qualified resected sample for single-cell sequencing while others all have available samples for single-cell RNA sequencing. **b** Quality control of single-cell RNA data in NEOTIDE and real-world cohort including cell numbers and total counts per cell. **c** Clustering of major cell type lineage and comparison of different cell types regarding MPR status. T test was used to measure the significance.

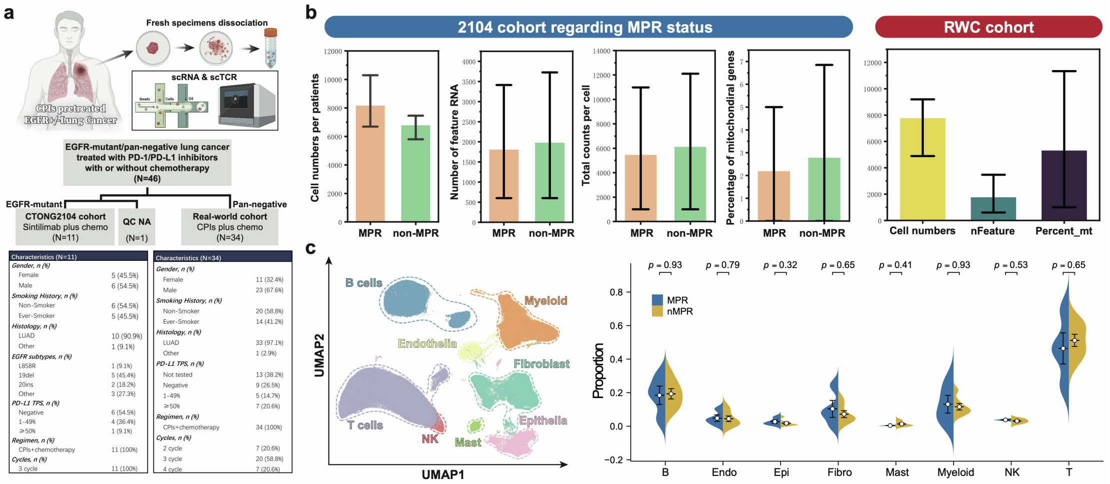

Supplementary Fig.10 Cell subsets annotation through specific feature genes, related to Figure 4, Figure 5 and STAR Methods. Bubble plots were generated to annotate different cell subtypes within diverse major lineage including CD4+lymphocytes (**a**), CD8+lymphocytes (**b**), B cells (**c**), myeloid cells (**d**) and innate immune cells (**e**).

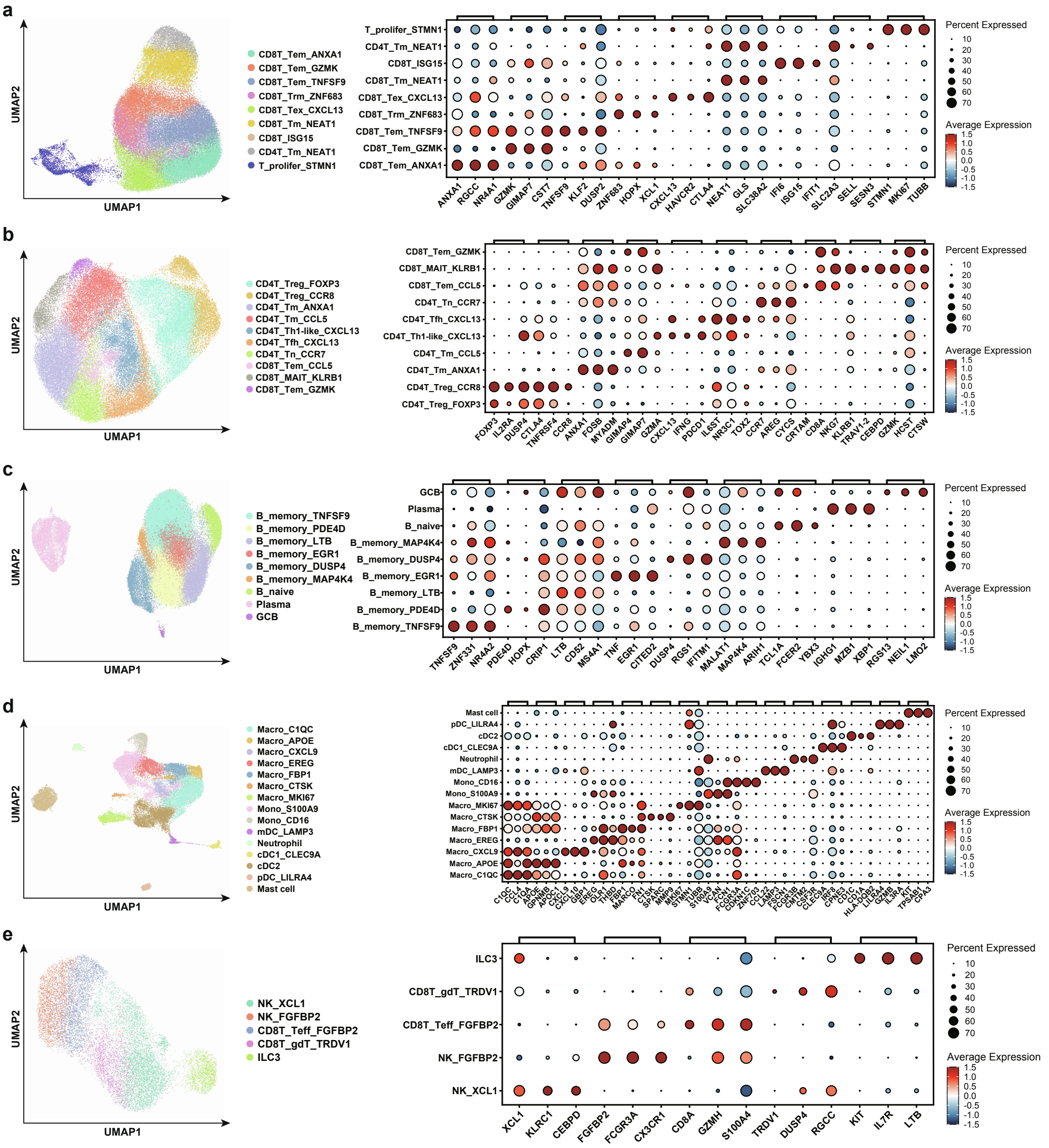

Supplementary Fig.11 Multiple immunohistochemistry staining of pre- and post-treatment specimens in responsive patients, related to Figure 5 and STAR Methods. HE, CD4, CD8 and Foxp3 staining was performed. Red dashed lines indicated tumor area and green arrow indicated staining cells.

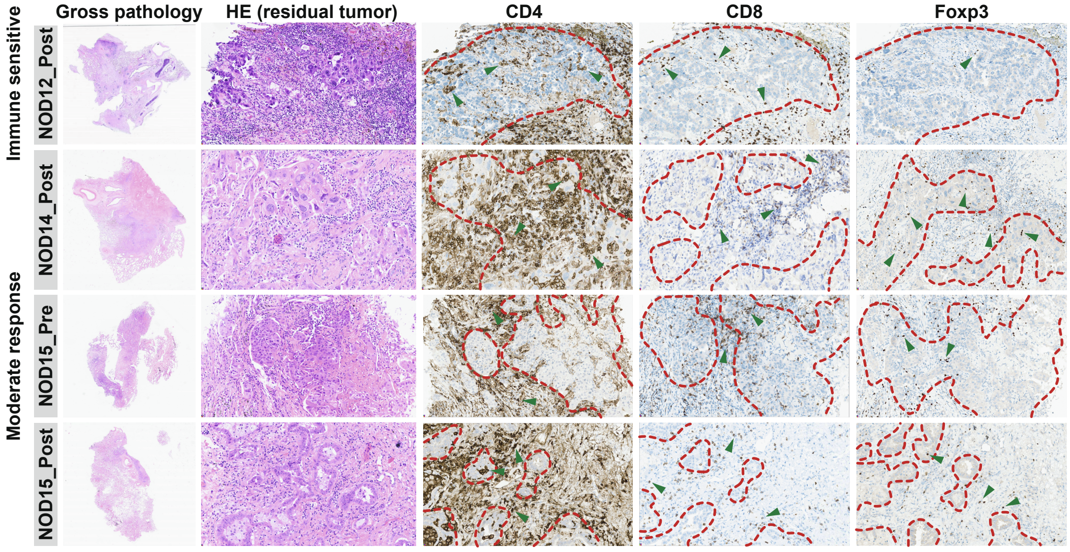

Supplementary Fig.12 Correlation of PD-L1 status and CCR8+Treg/CXCL13+Tex signature, related to Figure 5 and STAR Methods. Proportion of diverse PD-L1 status among different CCR8+Treg/CXCL13+Tex signature. Yates' correction was used to measure the significance.

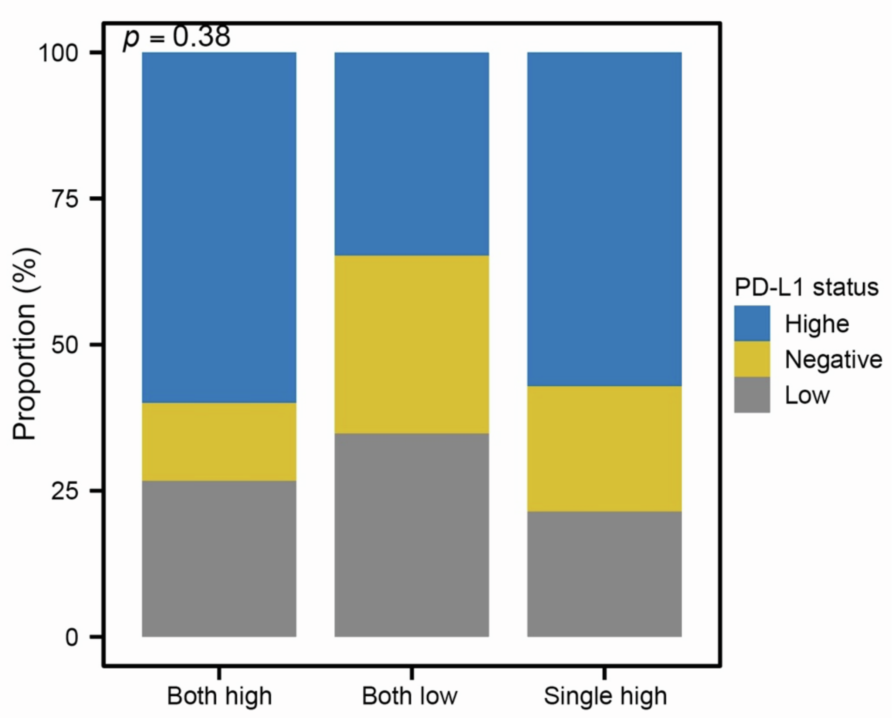

Supplementary Fig.13 External validation through public immunotherapy cohorts, related to Figure 5 and STAR Methods. a Response rate and overall survival of PD-L1 treated NSCLC cohort (Gainor et al). Stratified immune phenotypes were assessed through predominantly pre-treatment samples. Yates' correction was used to calculate the significance among groups. Log-rank p-value was conducted for survival analysis. **b** Response rate and progression-free survival of PD-1 treated melanoma cohort (Schadendorf et al). Stratified immune phenotypes were assessed through predominantly on-treatment samples. Yates' correction was used to calculate the significance among groups. Log-rank p-value was conducted for survival analysis. **c** Response rate and overall survival of PD-1 treated melanoma cohort (Chan et al). Stratified immune phenotypes were assessed through predominantly on-treatment samples. Chisq test was used to calculate the significance between groups. Log-rank p-value was conducted for survival analysis. **d** Proposed predictive value of CCR8^{+}Treg and CXCL13^{+}Tex before and after neoadjuvant immunotherapy in wild-type and EGFR-mutant NSCLC. PD, progressive disease; SD, stable disease; PR, partial response; CR, complete response; DCR, disease control rate; PD-1/PD-L1, PD-L1, programmed cell death protein 1/programmed cell death protein ligand-1; PFS, progression-free survival; OS, overall survival.

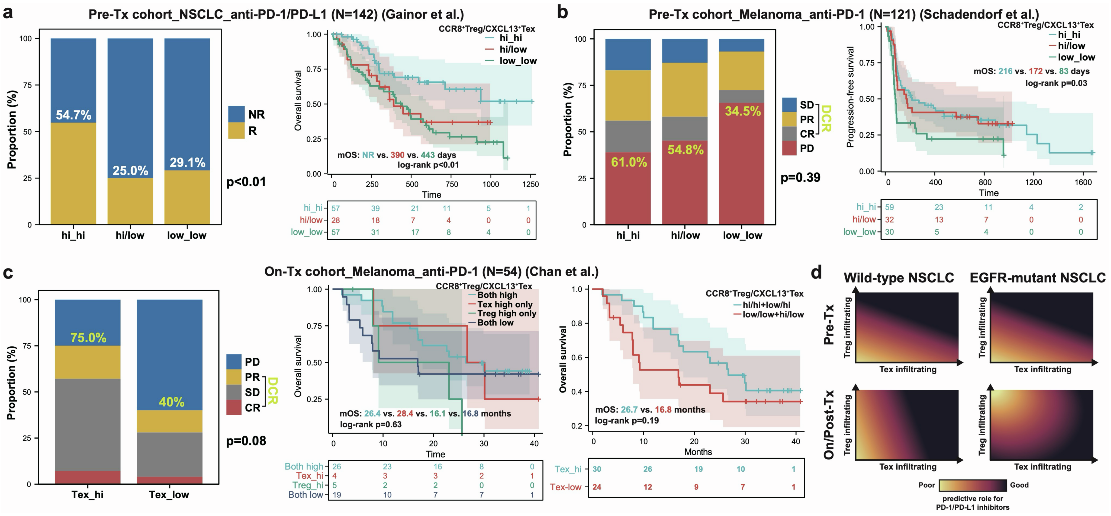

Supplementary Fig.14 Comparison of myeloid subtypes regarding response patterns, related to Figure 6. Proportion of different myeloid subtypes were compared in boxplot regarding response patterns. T test was used to measure the significance.

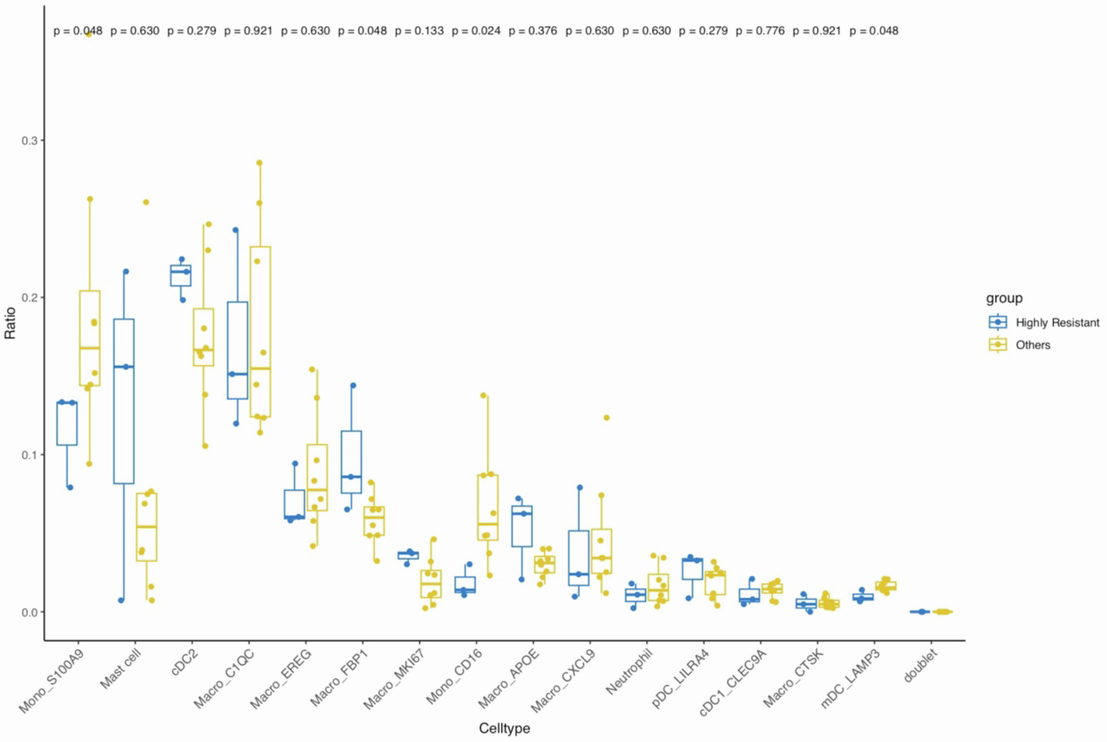

Supplement: Document S2. Article plus supplemental information [file mmc17.pdf]
